# Reference Values of the Inventory of Callous-Unemotional Traits in Youth Populations: A Systematic Literature Review

**DOI:** 10.1177/13591045261430966

**Published:** 2026-03-09

**Authors:** Patrícia Figueiredo, Beatriz Lopes, Melhyssa Gomes, Eduarda Ramião, Fernando Barbosa

**Affiliations:** 1 Department of Social and Behavioural Sciences, 112130 University of Maia, Maia, Portugal; 2 Laboratory of Neuropsychophysiology, Faculty of Psychology and Educational Sciences, 26706 University of Porto, Porto, Portugal; 3 School of Psychology and Life Science, Lusófona University, Lisbon, Portugal; 4 School of Health, Polytechnic Institute of Porto, Porto, Portugal

**Keywords:** inventory of callous-unemotional traits, ICU, callous-unemotional traits, reference values

## Abstract

Callous-unemotional (CU) traits are directly related to psychopathic traits, as they are based on features such as emotional insensitivity, lack of empathy, and lack of remorse. These traits typically develop during childhood and adolescence. Identifying them is extremely important, as they are predictors of antisocial behavior and the potential development of psychopathic traits in adulthood. The Inventory of Callous-Unemotional Traits (ICU) has been widely used to measure callous-unemotional traits in children and adolescents. A systematic literature review was conducted to establish reference values for the ICU in these age groups. Using specific inclusion and exclusion criteria, relevant studies were identified through databases and manual searches. The review included articles with empirical and quantitative methodologies. A total of 297 studies were included. Across regions, ICU scores were consistently higher in clinical, Disruptive Behavior Disorder-diagnosed, and forensic samples than in community samples, supporting the role of CU traits as markers of elevated psychopathological risk. Subscale analyses showed that Callousness and Uncaring more reliably differentiated high-risk groups than the Unemotional subscale. Substantial regional variation was observed, with particularly elevated community scores in Asian samples and broadly comparable forensic scores across Western regions. ICU scores vary systematically by sample type, region, and subscale, underscoring the importance of context-sensitive interpretation. The pooled reference values provided in this review offer an empirical framework for comparative and clinical interpretation of ICU scores in the absence of standardized norms or cutoffs.

## Plain Language Summary

Callous unemotional traits describe patterns such as low empathy, reduced emotional sensitivity, and little feeling of guilt or remorse. These traits usually begin to appear during childhood and adolescence and are important to identify because they are linked to higher risks of serious behavior problems and antisocial behavior later in life. In some cases, they may also be associated with the development of psychopathic traits in adulthood. The Inventory of Callous–Unemotional Traits (ICU) is one of the most commonly used questionnaires to measure these traits in children and adolescents. However, understanding what an ICU score means can be difficult without clear reference values showing what scores are typical for different groups. This study reviewed nearly 300 scientific studies from different regions of the world to summarize reference values for ICU scores in young people. The results show that ICU scores are generally higher in clinical groups, in youth diagnosed with disruptive behavior disorders, and in forensic samples than in children and adolescents from the general community. This supports the idea that higher CU traits are linked to increased psychological and behavioral risk. The review also found that some parts of the ICU, especially those measuring lack of care for others and lack of concern about performance, were better at distinguishing high-risk groups than items focused only on emotional expression. ICU scores also varied across regions, with higher average scores in some Asian community samples and similar patterns in forensic samples across Western countries. Overall, this review highlights the importance of considering context, sample type, and cultural background when interpreting ICU scores and provides useful reference values to support research and clinical decision-making.

Research has shown that psychopathic traits are essential for understanding antisocial behavior in adults, as individuals with pronounced psychopathic traits tend to display a particularly persistent, severe, and aggressive pattern of antisocial behavior (Anderson & Kiehl, 2014; Frick et al., 2003; Gendreau et al., 2002; Hare, 2003; Vitacco & Vincent, 2006). While psychopathic traits were once considered a significant specifier for severe antisocial and aggressive behaviors in adults, they are now recognized as a relevant factor in the development of conduct disorder in youth (Frick et al., 2000; Pisano et al., 2017; Ručević & Andershed, 2022). Over the past two decades, these traits in child and adolescent samples have been consistently linked to more severe and persistent patterns of conduct disorder and aggression, lower levels of social competence and prosocial behavior, delinquency and adult antisocial behavior, and adult psychopathy (Colins et al., 2024; Frick et al., 2014; Lynam et al., 2009; Salekin, 2017; Salekin et al., 2022, 2025). Several of these findings have even been replicated in early childhood research (e.g., Colins et al., 2021; Ezpeleta et al., 2013; López-Romero et al., 2022). Expanding the psychopathy construct to youth has thus become an important focus of research (Byrd et al., 2013; Harris et al., 2024; Ngo et al., 2024; Salekin & Lynam, 2011), with a particular emphasis on callous-unemotional (CU) traits (Frick & Ellis, 1999; Frick & Hare, 2001).

Callous-unemotional traits refer to specific deficits in affective experience and interpersonal style, characterized by a lack of guilt, lack of empathy, disregard for others for personal gain, and diminished emotional expression (Cooke et al., 2006; Fanti et al., 2009; Frick & White, 2008; Kimonis et al., 2015; Muñoz et al., 2011). Evidence from previous studies suggests that these traits are relatively stable from childhood through adolescence (Frick et al., 2003; Obradović et al., 2007), at least when compared to other measures of childhood personality and psychopathology (Frick et al., 2003). While these traits do not necessarily result in adverse outcomes (Kimonis, Frick, Munoz, & Aucoin, 2008), they may serve as significant predictors of distinct patterns of aggressive and violent behavior in youth populations.

Compared to other antisocial youth, those with CU traits are more likely to exhibit impairments in processing negative emotional stimuli (Blair, 1999; Blair et al., 2001; Kimonis, Frick, Munoz, & Aucoin, 2008; Kohls et al., 2025; Loney et al., 2003), show lower levels of fear and anxiety (Frick et al., 2003; Frick & Ellis, 1999; Lynam et al., 2005; Michielsen et al., 2022), and display diminished sensitivity to punishment cues - particularly in contexts where behavior is motivated by potential rewards (Barry et al., 2000; Fisher & Blair, 1998; Paz et al., 2024; Sakki et al., 2024).

It is worth noting, however, that Cleckley’s original conceptualization of psychopathy emphasized that its core traits - comparable to CU traits - can be present in individuals who do not display antisocial behavior (Cleckley, 1976). Indeed, later longitudinal studies suggest that CU traits can appear independently of antisocial behavior (e.g., Barker et al., 2011; Fontaine et al., 2011; Frick et al., 2003; Rowe et al., 2010), though they are often associated with later antisocial outcomes (e.g, Squillaci & Benoit, 2021). Moreover, youth exhibiting CU traits typically demonstrate other disruptive characteristics, such as poor peer relationships, low prosocial behavior, and increased hyperactivity (e.g., Fontaine et al., 2011; Matlasz et al., 2022; Pueyo et al., 2024; Zych et al., 2019). Thus, CU traits may serve as a potential clinical marker of psychiatric vulnerability and psychosocial maladjustment, and may also aid in the subtyping of children and adolescents with disruptive behavior disorders (Donohue et al., 2023; Ezpeleta et al., 2017; Johnson et al., 2014; Wright et al., 2019).

Several tools have been developed to assist in the assessment of both general psychopathy levels and specific psychopathic traits in adolescence and even childhood, including the Psychopathy Checklist: Youth Version (PCL: YV; Forth et al., 2003), the Child Psychopathy Scale (CPS; Lynam, 1997), and the Youth Psychopathic Traits Inventory (YPI; Andershed et al., 2002). Although several instruments include subscales for assessing CU traits, the Inventory of Callous-Unemotional Traits (ICU; Frick, 2004) was explicitly developed as a comprehensive and independent measure, enabling a more detailed and focused assessment of the affective dimensions of psychopathy (Essau et al., 2006).

## Inventory of Callous-Unemotional Traits

The ICU (Frick, 2004) was developed to assess CU traits in children and adolescents. It evolved from the Antisocial Process Screening Device (APSD; Frick & Hare, 2001), which in turn was based on the Psychopathy Checklist–Revised (PCL-R; Hare, 2003). Since its development in 2004, the ICU has been translated into more than 28 languages and validated across a wide range of developmental stages, cultural contexts, and assessment settings. Validation studies have supported its use in child samples (e.g., Benesch et al., 2014; Ezpeleta et al., 2013; Figueiredo et al., 2022, 2023; Houghton et al., 2013; Kimonis et al., 2015), as well as in adolescent samples drawn from community settings (e.g., Byrd et al., 2013; Carvalho et al., 2018; Essau et al., 2006) and forensic settings (Pechorro et al., 2016, 2017a, 2017b, 2018; Kimonis, Frick, Skeem, et al., 2008). Although the ICU is also widely used in clinical contexts, no validation study has been conducted specifically in clinically referred samples, meaning that its psychometric properties in these populations are typically inferred from community- and forensic-based research. Today, the ICU is widely recognized and used internationally as a key tool for assessing CU traits in children and adolescents.

The ICU consists of 24 items rated on a 4-point Likert scale, ranging from 0 (= *Not at all true*) to 3 (= *Definitely true*). There are five versions of the ICU with identical content but slight wording differences: (a) parent and teacher versions for preschool-aged children; (b) parent and teacher versions for school-aged children; and self-report versions for (c) school-aged children, (d) adolescents, and (e) adults. The first validation study of the ICU was conducted by Essau et al. (2006) in a sample of adolescents and proposed a three-factor model: (a) *Unemotional* (lack of emotional expression); (b) *Uncaring* (lack of concern for performance and others’ feelings); and (c) *Callousness* (lack of empathy, remorse, and guilt). Over the years, many other studies have examined the psychometric properties of the ICU across different countries, languages (e.g., English, Chinese, German, Norwegian, Portuguese, and Spanish), age groups, and settings, with findings suggesting variability in its factor structure - such as two-factor models (*Uncaring* and *Callousness*; Carvalho et al., 2018; Figueiredo et al., 2022, 2023; Kimonis et al., 2015; Willoughby et al., 2015) and three-factor models (*Uncaring*, *Unemotional*, and *Callousness*; Benesch et al., 2014; Essau et al., 2006; Ezpeleta et al., 2013; Pechorro et al., 2016, 2017a, 2017b, 2018). This heterogeneity suggests that the factor structure of the ICU may vary across developmental stage, language, culture, and assessment context, underscoring the importance of accounting for these sources of variability when interpreting ICU scores.

Despite its widespread use in research and clinical settings, the ICU lacks empirically established normative values or clinically validated cutoff scores for children and adolescents. To date, no study has defined thresholds that reliably distinguish between typical and clinically significant levels of callous–unemotional traits across age groups, cultures, or assessment settings. As a result, ICU scores are generally interpreted relatively or descriptively, often by comparing individuals or groups within a given sample rather than against standardized reference values. This lack of agreed-upon norms and cutoffs represents an important limitation for both research and clinical practice. It underscores the need for studies that provide reference values across different populations and contexts.

An essential issue in the literature concerns the compatibility and comparability of CU trait assessment measures in children and adolescents. Establishing equivalence across measures is necessary for scientific purposes, as inconsistencies between instruments can obscure findings and potentially lead to inaccurate or misleading conclusions (Borsboom, 2006). More specifically, if CU trait scores systematically vary due to factors such as instrument factor structure or individual characteristics like gender or age, there is a risk of misinterpreting, misclassifying, or underestimating the presence of CU traits in certain youth groups.

For the above reasons, establishing reference values for the ICU can be especially valuable for its practical application and the interpretation of results, both in clinical practice and in scientific research related to CU traits in youth populations. Thus, the present study aims to systematically review the use of the ICU and identify reference values for this inventory.

## Method

The systematic literature review was conducted according to the Preferred Reporting Items for Systematic Reviews and Meta-Analyses (PRISMA; Page et al., 2021). These guidelines are designed to ensure the quality, transparency, and consistency.

### Search and Study Selection Strategy

The research for the studies was conducted until June 2025 across multiple databases, namely EBSCO, PubMed, and Web of Science. The EBSCOhost platform provides integrated access to several key databases in psychology and health sciences, including APA PsycNet, PsycINFO, Scopus, and the Cochrane Library, ensuring broad coverage of the relevant literature. The following search expression was applied across all platforms: FX (“Inventory of Callous-Unemotional Traits” OR ICU) AND AB (juvenile* OR adolescen* OR youth) NOT AB (“intensive care unit”). This database search was supplemented by a manual search through the reference lists of the selected studies.

The SPIDER criteria (Sample, Phenomenon of Interest, Design, Evaluation, and Research type; Cooke et al., 2012) were employed to define the inclusion and exclusion criteria (Camilo & Garrido, 2019). Accordingly, the inclusion criteria were as follows: (1) Type of publication – the study must present empirical and quantitative data; (2) Population – the sample had to consist of children and adolescents up to 20 years of age. Although contemporary developmental frameworks conceptualize adolescence and youth as extending into the mid-twenties (up to 26 years; see Sawyer et al., 2018), the present review adopted a more conservative upper age limit to ensure that the included samples reflected individuals primarily with childhood and adolescence, minimizing developmental heterogeneity associated with the transition to adulthood; (3) Outcome – the study must report scores for the ICU total and/or its subscales, providing means (*M*) and standard deviations (*SD*) (or another statistical measure that allows for variance calculation). When such data were not reported, the primary authors of the studies were contacted and asked to provide them.

The exclusion criteria were as follows: (1) Type of publication – unpublished articles, reviews, theoretical papers, commentaries, case reports, theses, dissertations, and editorials were excluded; (2) Population – studies including adult participants were excluded unless separate data were provided for participants aged 20 years or younger; Samples consisting of children or adolescents with primary diagnoses of autism spectrum disorder, anxiety disorders, or depressive disorders were excluded; (3) Outcome – (a) studies that did not report means and standard deviations, even after attempts to obtain them by contacting the corresponding authors via email, were excluded; (b) studies were excluded if they used modified versions of the ICU, defined as versions in which items were added, removed, or assigned to factor structures that differed from the original 24-item instrument, unless sufficient psychometric information was provided to support their equivalence. Sample type (community, forensic, clinical, community with DBD, or clinical with DBD) was determined based on the recruitment setting and diagnostic information explicitly reported in each study. A diagnosis of disruptive behavior disorder (DBD) was considered present only when established using standardized diagnostic criteria (e.g., DSM or ICD) or validated diagnostic instruments. It should be noted that when the same sample was used across multiple studies, results were considered only once to avoid duplication.

Two independent reviewers selected the studies based on abstract screening, as recommended by the PRISMA guidelines (Page et al., 2021). Inter-rater agreement for the study selection process was assessed using Cohen’s Kappa (Landis & Koch, 1977), revealing substantial agreement, *K* = .86, *p* < .001. Discrepancies between reviewers were discussed and resolved by consensus.

### Quality and Risk Bias of Quantitative Studies

The Quantitative Research Assessment Tool (QRAT; Child Care & Early Education Research Connections, 2019) was used to assess the methodological quality of the studies included in this review. The QRAT comprises 12 items regarding the methodological features of the studies. Items can be rated −1, 0, 1, or NA (not applicable), except for the 12th question, where NA is not an option. According to the QRAT specifications, studies with lower scores should be regarded with more caution compared with studies that have higher scores, which are methodologically more robust. Most of the studies included in this review (89%) obtained a score higher than seven (see Supplemental Table 1).

### Identification and Screening

A total of 998 articles, published between 2006 and 2025, were identified across all databases as mentioned above. A total of 206 studies were removed as duplicates, leaving 792 for screening. Based on the abstract review, 195 articles were excluded due to: (a) type of publication (*n* = 37 studies); (b) population type (*n* = 24); and (c) failure to include an assessment of CU traits and/or use of the ICU (*n* = 133). In total, 600 articles were retained for full-text review, from which 312 were excluded for the following reasons: (a) incorrect publication type (*n* = 17); (b) inclusion of individuals over the age of 20 in the sample (*n* = 25); (c) failure to assess CU traits using the ICU (*n* = 54); (d) absence of reported means and/or standard deviations (or other statistics allowing for variance calculation) (*n* = 201); (e) studies using repeated samples (*n* = 4); (f) the full text of the study was not accessible, and the authors did not respond to the request for access (*n* = 7); and (g) composite scores combining different ICU versions (e.g., parent + teacher) (*n* = 4). After full-text screening, 288 studies were included in this systematic review, supplemented by 11 additional records identified from other sources, for a total of 299 studies (see Figure 1).

**Figure 1.**
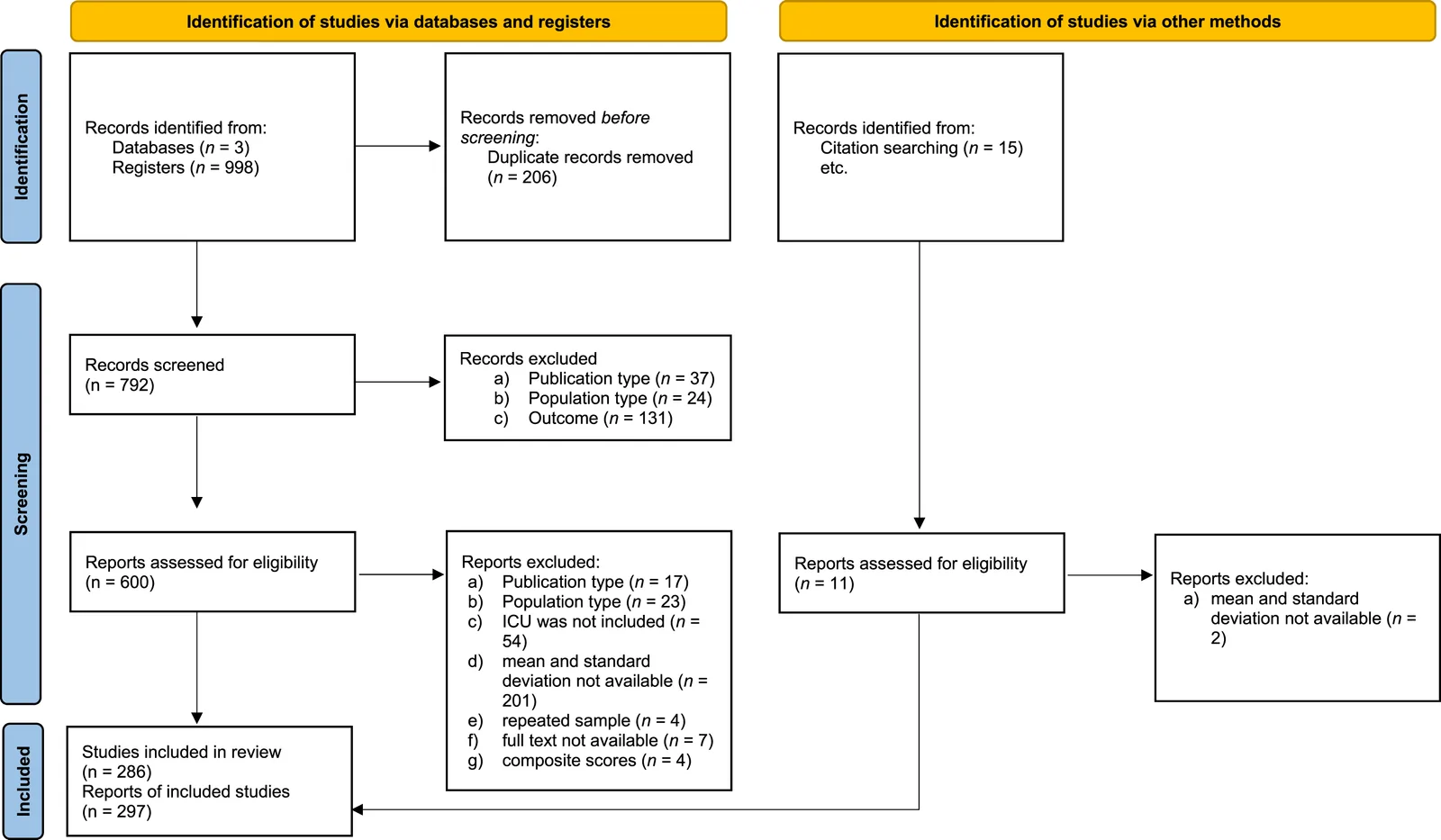
Flowchart of the literature review process

### Statistical Analysis

To provide reference values for the total ICU scores and their respective subscales, means and standard deviations from all studies included in this systematic review were compiled. The pooled mean was calculated (by summing the means multiplied by their respective sample sizes and dividing the result by the sum of the sample sizes), along with the pooled variance - which estimates the variance across different populations with differing means - and the pooled standard deviation (the square root of the pooled variance) for the total scores of the several available ICU versions, as well as for each subscale (i.e., *Callous*/*Callousness*, *Uncaring*, and/or *Unemotional*). It is important to note that only results from the same Likert scale and factorial structure were pooled. Given prior evidence that ICU scores and factor structures may vary systematically as a function of developmental stage, language, culture, and assessment context, all pooled estimates were stratified by sample type, sex, geographic region, and ICU version to preserve comparability and to avoid conflating meaningfully different populations. Thus, this procedure was conducted separately for each *sample-type*, namely: (a) forensic samples, referring to participants recruited from institutions within the justice system (e.g., juvenile justice facilities, detention centers, or court-mandated services); (b) community samples, consisting of participants drawn from the general population in non-clinical settings; (c) community samples with a diagnosis of Disruptive Behavior Disorders (DBD), which include participants recruited from community settings who met formal diagnostic criteria for a DBD; (d) clinical samples, comprising participants recruited from mental health or clinical services due to psychological or psychiatric concerns; and (e) clinical samples with a diagnosis of DBD, referring to participants assessed in clinical settings who met diagnostic criteria for a DBD. These analyses were further stratified by *sex* (male, female, and mixed samples) and *geographic region* (North America, Europe, Asia, and Oceania), accounting for the different versions of the ICU available.

## Results

A summary of the characteristics of the studies included in this systematic review is presented in the Supplemental Table 2. The included studies (*n* = 297) were published between 2006 and 2025. Most articles used samples from the general community (*n* = 159), while 64 studies used samples from individuals involved in the criminal justice system/juvenile courts. The remaining studies (*n* = 50) included clinical samples and community and clinical samples with DBD diagnosis (*n* = 24). All participants were between 3 and 20 years old.

Table 1 to 5 present the reference values for the various available versions of the ICU and their respective subscales, namely pooled means (*M*_*p*_), standard deviations (*SD*_*p*_,) and variances (*S*^2^_*p*_), by *sample-type*, *geographic region*, and *sex*.

**Table 1.** Meta-Analytic Analysis for Community Samples: Pooled Means, Standard Deviations and Variances for the Inventory of Callous-Unemotional Traits

Statistical parameter	Europe			North America			Asia			Oceania		
	Sex			Sex			Sex			Sex		
	Both	Male	Female	Both	Male	Female	Both	Male	Female	Both	Male	Female
ICU – Self-report, total (24 items), Likert 0-3												
N	19,479	3,377	4,349	14,989	3,784	826	6,238	---	---	---	---	---
Pooled mean	22.90	25.15	18.54	25.99	25.63	18.98	47.00	---	---	---	---	---
Pooled variance	64.27	58.99	56.50	108.88	67.82	65.71	74.21	---	---	---	---	---
Pooled SD	8.02	7.68	7.52	10.43	8.24	8.11	8.61	---	---	---	---	---
ICU – Self-report, total (22 items), Likert 0-3												
N	---	---	---	7,136	3,297	---	---	---	---	---	---	---
Pooled mean	---	---	---	23.11	22.44	---	---	---	---	---	---	---
Pooled variance	---	---	---	63.86	70.66	---	---	---	---	---	---	---
Pooled SD	---	---	---	7.99	8.41	---	---	---	---	---	---	---
ICU – Self-report, total (19 items), Likert 0-3												
N	1,382	---	---	---	---	---	---	---	---	---	---	---
Pooled mean	22.56	---	---	---	---	---	---	---	---	---	---	---
Pooled variance	58.30	---	---	---	---	---	---	---	---	---	---	---
Pooled SD	7.64	---	---	---	---	---	---	---	---	---	---	---
ICU – Self-report, total (12 items), Likert 0-3												
N	1,869	---	---	---	---	---	---	---	---	---	---	---
Pooled mean	20.44	---	---	---	---	---	---	---	---	---	---	---
Pooled variance	50.19	---	---	---	---	---	---	---	---	---	---	---
Pooled SD	7.08	---	---	---	---	---	---	---	---	---	---	---
ICU – Self-report, unemotional subscale (5 items), Likert 0-3												
N	6,647	1,074	979	3,226	749	---	---	---	---	---	---	---
Pooled mean	10.65	7.69	6.21	8.12	7.87	---	---	---	---	---	---	---
Pooled variance	31.12	7.63	9.06	9.42	7.47	---	---	---	---	---	---	---
Pooled SD	5.58	2.76	3.01	3.07	2.73	---	---	---	---	---	---	---
ICU – Self-report, callousness subscale (11 items), Likert 0-3												
N	9,386	1,074	979	2670	---	---	3,074	816	---	---	---	---
Pooled mean	10.48	7.77	5.30	14.17	---	---	3.06	10.21	---	---	---	---
Pooled variance	31.05	18.55	12.49	41.47	---	---	10.08	30.62	---	---	---	---
Pooled SD	5.57	4.31	3.53	6.44			3.18	5.53	---	---	---	---
ICU – Self-report, callousness subscale (9 items), Likert 0-3												
N	917	---	---	799	633	---	---	---	---	---	---	---
Pooled mean	5.41	---	---	7.32	6.43	---	---	---	---	---	---	---
Pooled variance	16.43	---	---	22.45	18.11	---	---	---	---	---	---	---
Pooled SD	4.05	---	---	4.74	4.26	---	---	---	---	---	---	---
ICU – Self-report, uncaring subscale (8 items), Likert 0-3												
N	10,303	1,226	1,122	3,226	749	---	999	---	---	---	---	---
Pooled mean	10.81	8.28	6.37	12.31	11.11	---	9.39	---	---	---	---	---
Pooled variance	26.12	17.99	12.80	26.01	26.27	---	2317.23	---	---	---	---	---
Pooled SD	5.11	4.24	3.58	5.10	5.13	---	48.14	---	---	---	---	---
ICU – Parent version, total (24 items), likert 0-3												
N	3,531	1,246	---	7,388	1,128	1,228	505	---	---	1,363	---	---
Pooled mean	18.55	18.68	---	22.52	19.85	16.07	27.80	---	---	21.72	---	---
Pooled variance	80.62	71.16	---	83.32	78.27	67.36	31.48	---	---	139.34	---	---
Pooled SD	8.98	8.44	---	9.13	8.45	8.21	5.61	---	---	11.80	---	---
ICU – Parent version, total (22 items), Likert 0-3												
N	---	---	---	339	---	---		---	---	---	---	---
Pooled mean	---	---	---	18.79	---	---	---	---	---	---	---	---
Pooled variance	---	---	---	91.69	---	---	---	---	---	---	---	---
Pooled SD	---	---	---	9.58	---	---	---	---	---	---	---	---
ICU – Parent version, total (11 items), Likert 0-3												
N	---	---	---	---	---	---	2499	---	---	---	---	---
Pooled mean	---	---	---	---	---	---	20.57	---	---	---	---	---
Pooled variance	---	---	---	---	---	---	28.95	---	---	---	---	---
Pooled SD	---	---	---	---	---	---	5.38	---	---	---	---	---
ICU – Parent version, callousness subscale (11 items), Likert 0-3												
N	354	---	---	1,221	---	---	---	---	---	---	---	---
Pooled mean	4.39	---	---	4.54	---	---	---	---	---	---	---	---
Pooled variance	13.54	---	---	14.47	---	---	---	---	---	---	---	---
Pooled SD	3.68	---	---	3.80	---	---	---	---	---	---	---	---
ICU – Parent version, uncaring subscale (8 items), Likert 0-3												
N	353	---	---	1,666	---	---	842	---	---	---	---	---
Pooled mean	11.79	---	---	10.85	---	---	9.67	---	---	---	---	---
Pooled variance	22.43	---	---	24.19	---	---	5.01	---	---	---	---	---
Pooled SD	4.74	---	---	4.92	---	---	2.24	---	---	---	---	---
ICU – Parent version, unemotional subscale (5 items), Likert 0-3												
N	354	---	---	1,221	---	---	---	---	---	---	---	---
Pooled mean	4.15	---	---	4.72	---	---	---	---	---	---	---	---
Pooled variance	5.92	---	---	8.70	---	---	---	---	---	---	---	---
Pooled SD	2.43	---	---	2.95	---	---	---	---	---	---	---	---
ICU – Teacher version, total (24 items), Likert 0-3												
N	2576	1,002	---	1,930	---	---	---	---	---	---	---	---
Pooled mean	22.47	22.37	---	38.99	---	---	---	---	---	---	---	---
Pooled variance	97.94	75.44	---	142.05	---	---	---	---	---	---	---	---
Pooled SD	9.89	8.69	---	11.92	---	---	---	---	---	---	---	---
ICU – Teacher version, callousness subscale (11 items), Likert 0-3												
N	---	---	---	727	---	---	---	---	---	---	---	---
Pooled mean	---	---	---	18.39	---	---	---	---	---	---	---	---
Pooled variance	---	---	---	27.99	---	---	---	---	---	---	---	---
Pooled SD	---	---	---	5.29	---	---	---	---	---	---	---	---
ICU – Teacher version, uncaring subscale (8 items), Likert 0-3												
N	---	---	---	727	---	---	---	---	---	---	---	---
Pooled mean	---	---	---	13.03	---	---	---	---	---	---	---	---
Pooled variance	---	---	---	20.83	---	---	---	---	---	---	---	---
Pooled SD	---	---	---	4.56	---	---	---	---	---	---	---	---
ICU – Teacher version, unemotional subscale (5 items), Likert 0-3												
N	---	---	---	727	---	---	---	---	---	---	---	---
Pooled mean	---	---	---	10.75	---	---	---	---	---	---	---	---
Pooled variance	---	---	---	10.22	---	---	---	---	---	---	---	---
Pooled SD	---	---	---	3.20	---	---	---	---	---	---	---	---

*Note.* ICU = Inventory of Callous-Unemotional Traits; SD = standard deviation.

**Table 2.** Meta-Analytic Analysis for Community Samples With DBD Diagnosis: Pooled Means, Standard Deviations and Variances for the Inventory of Callous-Unemotional Traits

Statistical parameter	Europe			North America			Asia			Oceania		
	Sex			Sex			Sex			Sex		
	Both	Male	Female	Both	Male	Female	Both	Male	Female	Both	Male	Female
ICU – Self-report, total (24 items), Likert 0-3												
N	598	233	---	246	---	---	---	---	---	---	---	---
Pooled mean	33.59	37.90	---	35.37	---	---	---	---	---	---	---	---
Pooled variance	75.27	81.25	---	91.77	---	---	---	---	---	---	---	---
Pooled SD	8.68	9.01	---	9.58	---	---	---	---	---	---	---	---
ICU – Self-report, unemotional subscale (5 items), Likert 0-3												
N	301	---	---	148	---	---	---	---	---	---	---	---
Pooled mean	10.72	---	---	7.79	---	---	---	---	---	---	---	---
Pooled variance	16.69	---	---	9.91	---	---	---	---	---	---	---	---
Pooled SD	4.09	---	---	3.15	---	---	---	---	---	---	---	---
ICU – Self-report, uncaring subscale (8 items), Likert 0-3												
N	301	---	---	148	---	---	---	---	---	---	---	---
Pooled mean	7.61	---	---	17.16	---	---	---	---	---	---	---	---
Pooled variance	12.20	---	---	14.52	---	---	---	---	---	---	---	---
Pooled SD	3.49	---	---	3.81	---	---	---	---	---	---	---	---
ICU – Parent version, total (24 items), likert 0-3												
N	632	---	---	166	92	---	---	---	---	---	---	---
Pooled mean	26.66	---	---	38.80	39.02	---	---	---	---	---	---	---
Pooled variance	8.21	---	---	64.38	61.42	---	---	---	---	---	---	---
Pooled SD	2.86	---	---	8.02	7.84	---	---	---	---	---	---	---
ICU – Teacher version, total (24 items), Likert 0-3												
N	192	---	---	---	---	---	---	---	---	---	---	---
Pooled mean	40.63	---	---	---	---	---	---	---	---	---	---	---
Pooled variance	43.40	---	---	---	---	---	---	---	---	---	---	---
Pooled SD	6.59	---	---	---	---	---	---	---	---	---	---	---

*Note.* ICU = Inventory of Callous-Unemotional Traits; SD = standard deviation.

**Table 3.** Meta-Analytic Analysis for Clinical Samples: Pooled Means, Standard Deviations, and Variances for the Inventory of Callous-Unemotional Traits

Statistical parameter	Europe			North America			Asia			Oceania		
	Sex			Sex			Sex			Sex		
	Both	Male	Female	Both	Male	Female	Both	Male	Female	Both	Male	Female
ICU – Self-report, total (24 items), Likert 0-3												
N	3,084	497	---	2267	---	---	123	---	---	---	---	---
Pooled mean	31.12	23.58	---	27.47	---	---	25.88	---	---	---	---	---
Pooled variance	85.94	51.82	---	81.00	---	---	54.18	---	---	---	---	---
Pooled SD	9.27	7.20	---	9.00	---	---	7.36	---	---	---	---	---
ICU – Parent version, total (24 items), likert 0-3												
N	96	---	---	521	73	---	---	---	---	236	---	---
Pooled mean	29.23	---	---	24.50	18.55	---	---	---	---	26.79	---	---
Pooled variance	45.67	---	---	111.10	63.58	---	---	---	---	185.53	---	---
Pooled SD	6.76	---	---	10.54	7.97	---	---	---	---	13.62	---	---
ICU – Parent version, callousness (11 items), Likert 0-3												
N	---	---	---	1,954	---	---	---	---	---	---	---	---
Pooled mean	---	---	---	4.54	---	---	---	---	---	---	---	---
Pooled variance	---	---	---	10.44	---	---	---	---	---	---	---	---
Pooled SD	---	---	---	3.23	---	---	---	---	---	---	---	---
ICU – Parent version, uncaring (8 items), Likert 0-3												
N	---	---	---	1,954	---	---	---	---	---	---	---	---
Pooled mean	---	---	---	14.91	---	---	---	---	---	---	---	---
Pooled variance	---	---	---	21.57	---	---	---	---	---	---	---	---
Pooled SD	---	---	---	4.64	---	---	---	---	---	---	---	---
ICU – Teacher version, total (24 items), Likert 0-3												
N	---	---	---	236	---	---	---	---	---	---	---	---
Pooled mean	---	---	---	26.79	---	---	---	---	---	---	---	---
Pooled variance	---	---	---	185.53	---	---	---	---	---	---	---	---
Pooled SD	---	---	---	13.62	---	---	---	---	---	---	---	---

*Notes.* ICU = Inventory of Callous-Unemotional Traits; SD = standard deviation.

**Table 4.** Meta-Analytic Analysis for Clinical Samples With DBD Diagnosis: Pooled Means, Standard Deviations, and Variances for the Inventory of Callous-Unemotional Traits

Statistical parameter	Europe			North America			Asia			Oceania		
	Sex			Sex			Sex			Sex		
	Both	Male	Female	Both	Male	Female	Both	Male	Female	Both	Male	Female
ICU – Self-report, total (24 items), Likert 0-3												
N	1,780	141	---	566	---	---	---	---	---	---	---	---
Pooled mean	27.26	30.65	---	27.11	---	---	---	---	---	---	---	---
Pooled variance	83.70	77.22	---	104.79	---	---	---	---	---	---	---	---
Pooled SD	9.15	8.79	---	10.24	---	---	---	---	---	---	---	---
ICU – Parent-report, total (24 items), Likert 0-3												
N	234	---	---	173	---	---	---	---	---	---	---	---
Pooled mean	35.02	---	---	34.85	---	---	---	---	---	---	---	---
Pooled variance	93.34	---	---	111.12	---	---	---	---	---	---	---	---
Pooled SD	9.66	---	---	10.54	---	---	---	---	---	---	---	---

*Notes.* ICU = Inventory of Callous-Unemotional Traits; SD = standard deviation.

**Table 5.** Meta-Analytic Analysis for Forensic Samples: Pooled Means, Standard Deviations and Variances for the Inventory of Callous-Unemotional Traits

Statistical parameter	Europe			North America			Oceania		
	Sex			Sex			Sex		
	Both	Male	Female	Both	Male	Female	Both	Male	Female
ICU – Self-report, total (24 items), Likert 0-3									
N	1,707	1,001	237	13,250	11,033	396	---	821	---
Pooled mean	29.19	29.09	27.65	26.47	26.21	34.29	---	26.92	---
Pooled variance	100.44	96.50	100.05	66.50	65.63	65.03	---	76.06	---
Pooled SD	10.02	9.82	10.00	8.15	8.10	8.06	---	8.72	---
ICU – Self-report, total (22 items), Likert 0-3									
N	---	---	---	4,137	2552	271	---	---	---
Pooled mean	---	---	---	25.27	25.82	26.44	---	---	---
Pooled variance	---	---	---	73.10	69.81	65.54	---	---	---
Pooled SD	---	---	---	8.55	8.36	8.10	---	---	---
ICU – Self-report, unemotional subscale (5 items), Likert 0-3									
N	751	236	---	2199	1,177	---	---	459	---
Pooled mean	10.61	8.11	---	8.01	8.13	---	---	8.60	---
Pooled variance	20.32	9.42	---	8.43	8.24	---	---	7.55	---
Pooled SD	4.51	3.07	---	2.90	2.87	---	---	2.75	---
ICU – Self-report, callousness subscale (11 items), Likert 0-3									
N	751	236	---	1,427	692	---	---	459	---
Pooled mean	11.55	11.37	---	8.81	7.42	---	---	6.71	---
Pooled variance	30.66	26.29	---	34.68	40.12	---	---	18.96	---
Pooled SD	5.54	5.13	---	5.89	6.33	---	---	4.35	---
ICU – Self-report, callousness subscale (9 items), Likert 0-3									
N	---	---	---	772	485	---	---	---	---
Pooled mean	---	---	---	8.55	8.93	---	---	---	---
Pooled variance	---	---	---	20.29	21.84	---	---	---	---
Pooled SD	---	---	---	4.50	4.67	---	---	---	---
ICU – Self-report, uncaring subscale (8 items), Likert 0-3									
N	751	236	---	2199	1,177	---	---	459	---
Pooled mean	9.18	11.30	---	9.73	10.46	---	---	11.85	---
Pooled variance	16.70	18.93	---	23.03	22.94	---	---	23.09	---
Pooled SD	4.09	4.35	---	4.80	4.79	---	---	4.81	---
ICU – Self-report, total (24 items), Likert 0-3									
N	---	---	---	1,066	---	---	---	---	---
Pooled mean	---	---	---	27.08	---	---	---	---	---
Pooled variance	---	---	---	136.57	---	---	---	---	---
Pooled SD	---	---	---	11.69	---	---	---	---	---

*Note.* ICU = Inventory of Callous-Unemotional Traits; SD = standard deviation.

Regarding the self-report version of the ICU (24 items, 0–3 Likert scale), in Europe, community samples of both sexes exhibited lower mean scores (*M*_*p*_ = 22.90, *SD*_*p*_ = 8.02, *n* = 19,479) compared to community samples with DBD diagnosis (*M*_*p*_ = 33.59, *SD*_*p*_ = 8.68, *n* = 598), clinical samples (*M*_*p*_ = 31.12, *SD*_*p*_ = 9.27, *n* = 3,084), clinical samples with DBD diagnosis (*M*_*p*_ = 27.26, *SD*_*p*_ = 9.15, *n* = 1,780) and forensic samples (*M*_*p*_ = 29.19, *SD*_*p*_ = 10.02, *n* = 1,707). Within these groups, male participants tended to show the highest scores. However, this pattern was not observed in North America. That is, community samples showed lower average scores (*M*_*p*_ = 25.99, *SD*_*p*_ = 10.43, *n* = 14,989), followed by forensic samples (*M*_*p*_ = 26.47, *SD*_*p*_ = 8.15, *n* = 13,250), clinical samples (*M*_*p*_ = 27.47, *SD*_*p*_ = 9.00, *n* = 2,267), clinical samples with DBD diagnosis (*M*_*p*_ = 27.11, *SD*_*p*_ = 10.24, *n* = 566), with community samples with DBD diagnosis presenting the highest scores (*M*_*p*_ = 35.37, *SD*_*p*_ = 9.58, *n* = 246).

A more detailed analysis ICU self-report subscales indicated distint patterns across regions and sample types. In Europe, clear diferences emerged across the sample types on all ICU subscales. For Callousness (11 items, 0–3 Likert scale), pooled means were lowest in general community samples (*M*_*p*_ = 10.48, *SD*_*p*_ = 5.57, *n* = 9,386), increased slightly in forensic samples (*M*_*p*_ = 11.55, *SD*_*p*_ = 5.54, *n* = 751), and were highest in community samples with DBD, for which subscale-level data indicate elevated CU traits relative to non-diagnosed community youths. Parent-reported *Callousness* scores in clinical samples were comparatively low (*M*_*p*_ = 4.39–4.54), suggesting informant-related differences. A similar gradient was observed for *Uncaring* (8 items, 0–3 Likert scale), with general community samples showing moderate pooled means (*M*_*p*_ = 10.81, *SD*_*p*_ = 5.11, *n* = 10,303), forensic samples presenting slightly lower values (*M*_*p*_ = 9.18, *SD*_*p*_ = 4.09, *n* = 751), and community samples with DBD displaying clearly elevated scores. For the *Unemotional* subscale (5 items, 0–3 Likert scale), forensic youths showed the highest pooled mean (*M*_*p*_ = 10.61, *SD*_*p*_ = 4.51, *n* = 751), followed closely by community samples with DBD (*M*_*p*_ = 10.72, *SD*_*p*_ = 4.09, *n* = 301), whereas general community samples showed slightly lower but comparable levels (*M*_*p*_ = 10.65, *SD*_*p*_ = 5.58, *n* = 6,647).

In North America, differences across the five samples were more pronounced, particularly for the Uncaring subscale. General community samples showed moderate pooled means for *Callousness* (*M*_*p*_ = 14.17, *SD*_*p*_ = 6.44, *n* = 2,670), *Uncaring* (*M*_*p*_ = 12.31, *SD*_*p*_ = 5.10, *n* = 3,226), and *Unemotional* traits (*M*_*p*_ = 8.12, *SD*_*p*_ = 3.07, *n* = 3,226). In contrast, community samples with DBD presented markedly elevated *Uncaring* scores (*M*_*p*_ = 17.16, *SDp* = 3.81, *n* = 148), exceeding those observed in all other sample types. Forensic samples showed lower *Callousness* pooled means (*M*_*p*_ = 8.81, *M*_*p*_ = 5.89, *n* = 1,427) than community samples, but higher *Uncaring* (*M*_*p*_ = 9.73, *SD*_*p*_ = 4.80, *n* = 2,173) and comparable *Unemotional* scores (*M*_*p*_ = 8.01, *SD*_*p*_ = 2.90, *n* = 2,199). Although subscale-level data were not available for North American clinical samples with DBD, elevated total ICU scores in these groups suggest generally high CU trait levels.

Data from Asia were available primarily for community samples. Asian community youths showed substantially higher pooled ICU total scores than European and North American community samples (*M*_*p*_ = 47.00, *SD*_*p*_ = 8.61, i = 6,238), indicating markedly elevated CU trait levels in this region. At the subscale level, Asian community samples also exhibited comparatively high Callousness (*M*_*p*_ = 10.21, *SD*_*p*_ = 5.53, *n* = 816) and Uncaring scores (*M*_*p*_ = 9.39, *SD*_*p*_ = 48.14, *n* = 999), suggesting a broad elevation across affective and interpersonal CU dimensions relative to Western samples. Parent-reported ICU totals in Asia were likewise higher than those observed in Europe and North America (*M*_*p*_ = 27.80, *SD*_*p*_ = 5.61, *n* = 505) (Table 2). In Oceania, data were available for community and forensic samples. In community samples, parent-reported ICU total scores were relatively high (*M*_*p*_ = 21.72, *SD*_*p*_ = 11.80, *n* = 1,363), comparable to or exceeding those observed in European and North American community samples. In forensic samples, youths from Oceania showed ICU total scores similar to those observed in Europe and North America (*M*_*p*_ = 26.92, *SD*_*p*_ = 8.72, *n* = 821), with elevated Uncaring (*M*_*p*_ = 11.85, *SD*_*p*_ = 4.81) and Unemotional (*M*_*p*_ = 8.60, *SD*_*p*_ = 2.75) subscale scores.

## Discussion

The Inventory of Callous-Unemotional Traits (ICU) has been widely used to identify and assess callous-unemotional traits in children and adolescents for research and clinical or forensic practices. However, the literature analysis reveals considerable differences when scores are based on factors such as the instrument’s factorial structure and sex, which can lead to assessment inconsistencies and inaccurate conclusions, such as misclassifying or underestimating CU traits in specific groups. Therefore, the main goal of this study is to establish and provide reference values for the self- and other-report versions of the ICU in its more common factorial structures, considering different sample types (e.g., community, community with disruptive behavior disorder [DBD] diagnosis, clinical, clinical with DBD diagnosis, or forensic), participants’ sex, and geographic origin.

The ICU has been widely used based on various structural models. The results obtained reveal distinct patterns in the expression of callous-unemotional (CU) traits between European and North American samples, depending on both sample type (community, clinical, forensic) and informant type (self-report, parents, teachers). These variations suggest that cultural, methodological, and contextual factors may play a significant role in the manifestation of, or at least in the perception of, callousness, lack of empathy, and emotional detachment (Gendron, 2017).

Notably, the present review also provides insight into CU trait expression in underrepresented regions, particularly Asia and Oceania. Asian community samples showed substantially higher ICU total and subscale scores than their European and North American counterparts, suggesting that CU-related affective and interpersonal features may be more frequently endorsed or more strongly expressed in these cultural contexts. Cross-cultural research has shown that emotional expression, empathy, and social conformity are shaped by culturally embedded norms and values, which can influence both the development and the self-reporting of socio-affective traits (Markus & Kitayama, 1991; Mesquita & Leu, 2007; Shou et al., 2019). In addition, differences in response styles, such as greater acquiescence or reduced use of extreme response categories in some East Asian cultures, may further affect how ICU items are endorsed (Chen et al., 1995; He & van de Vijver, 2012).

In Oceania, although the number of available studies was smaller, both community and forensic samples showed ICU scores broadly comparable to those observed in Europe and North America, particularly in forensic contexts. This pattern is consistent with previous findings indicating that CU traits are robustly associated with antisocial and justice-involved behavior across Western cultures (Frick et al., 2014; Kimonis, Frick, Skeem, et al., 2008; Salekin et al., 2018). Nevertheless, the relatively limited number of studies from Oceania underscores the need for further research to establish more stable and culturally sensitive reference values for this region.

Regarding the self-report version of the ICU, it was observed that, in Europe, mean scores were, as expected, higher in clinical and forensic samples than in community samples. This pattern aligns well with the literature, which demonstrates a higher prevalence of CU traits among youth with behavioral disorders or contact with the justice system (Frick et al., 2014). The clear distinction between groups suggests that, in Europe, the criteria for inclusion in clinical and forensic samples more directly reflect elevated levels of these traits. Interestingly, this pattern is reversed in North America, where community samples showed the highest mean scores. This finding may reflect methodological differences in selecting community samples, including participants with high but undiagnosed risk factors, or differences in affective socialization. In social contexts where assertiveness, emotional independence, or less empathetic behavior are more accepted or valued, characteristics that may overlap with CU traits, these features may be more evident even in non-clinical populations (Kimonis, Frick, Skeem, et al., 2008). This hypothesis seems to be supported by subscale analyses: although community samples showed higher scores on the Callousness and Unemotional subscales in Europe, the highest Unemotional scores were found in clinical samples. This pattern suggests that the Unemotional dimension, often considered more internalized, may manifest more strongly in individuals with externalizing problems. In North America, all subscales showed surprisingly low scores in community samples, indicating a possible cultural difference in the perception and self-assessment of these traits.

Additionally, data from the ICU’s parent- and teacher-report versions show a more conventional pattern, with higher scores in clinical populations in Europe and North America. However, it is notable that North American teachers assigned significantly higher scores to community children than their European counterparts. This discrepancy may reflect differences in teachers’ perceptions of challenging behaviors, possibly linked to pedagogical practices, school norms, or distinct cultural expectations regarding children’s emotional and behavioral expressions (Achenbach et al., 2008). Moreover, it may reflect cultural diversity, meaning that cultural and social norms can influence how individuals express themselves and their traits are assessed (e.g., Shou et al., 2019). Therefore, it is essential to recognize that conducting research across different cultures does not guarantee measurement equivalence across groups. There may be changes in the instrument’s content, the testing process, or the tool’s underlying structure (Waltz et al., 2005).

Differences between community and clinical samples were pronounced in both Europe and North America, with youths recruited from clinical and DBD-diagnosed groups showing substantially higher ICU total scores than those from the general population. This pattern is consistent with theoretical models positing CU traits as a marker of severe and persistent antisocial behavior, particularly when embedded within clinical presentations of disruptive behavior disorders (Frick et al., 2014; Frick & White, 2008). In both regions, the elevation of ICU scores in clinical samples suggests that CU traits are not merely extreme variants of normative personality but are closely linked to psychopathological risk and functional impairment. Importantly, subscale patterns revealed nuances that were not always captured by total scores alone. In some cases, clinical and forensic samples showed disproportionately elevated Callousness and Uncaring scores, whereas Unemotional scores were less differentiated from those of community samples. This finding is in line with psychometric evidence indicating that the Unemotional subscale is less consistently associated with externalizing pathology and shows weaker reliability and validity across samples (Carvalho et al., 2018; Kimonis et al., 2015; Willoughby et al., 2015). The fact that Callousness and Uncaring more clearly distinguished high-risk groups supports their central role in the CU construct and helps explain why two-factor models often outperform three-factor models in applied settings. These regional patterns should also be interpreted considering cross-national differences in juvenile justice and mental health systems. In several Nordic and other European countries, youths under the age of 18 are rarely processed through the criminal justice system, even when serious antisocial behavior is present, and are instead referred to child welfare or mental health services rather than to juvenile courts (Lappi-Seppälä, 2011, 2018; Pratt & Eriksson, 2013). As a result, “forensic” samples in these contexts may represent a smaller and more severely affected subgroup than in countries such as the United States, where juvenile justice involvement is more common and institutional placement is more frequently used for adolescents with serious conduct problems (Abrams, 2013; Tonry & Farrington, 2005).

Meta-analytic data from clinical, community, and forensic samples reveal sex differences in the expression of CU traits, particularly in European and North American samples. Overall, the data indicate that boys score higher than girls on total ICU scores and the Callousness and Uncaring subscales, with this trend being more pronounced in clinical and community samples. These sex differences support previous literature showing a higher prevalence of CU traits in boys, especially concerning externalizing behaviors associated with conduct disorders and psychopathy risk (Essau et al., 2006; Frick et al., 2014). Gender socialization may contribute to this disparity, as boys are often encouraged to suppress emotions as well as empathy or vulnerability, which may facilitate the development of callous-unemotional traits or disruptive behavior. On the other hand, the pattern observed among girls, with higher scores on the Unemotional subscale in some contexts, raises the possibility of a more internalized profile of female psychopathy, less visible through disruptive behaviors but still clinically and functionally relevant. This profile may be underestimated in traditional assessments, prioritizing behavioral manifestations more typical of males (Fontaine et al., 2010).

In forensic contexts, available data refer primarily to boys, making direct comparisons with girls difficult. Nevertheless, boys in these samples continue showing high scores in Europe and North America. The available subscale data confirm this trend, especially in the callousness and uncaring dimensions. The scarcity of data on girls in this context may be related to their lower representation in the juvenile justice system or to the underestimation of disruptive behaviors in girls. CU traits in girls may manifest in less visible ways (e.g., social manipulation, masked CU traits), suggesting a more internalized or covert female CU profile (e.g., Cardoso et al., 2023).

Despite the consistency and validity of the ICU, it is essential to consider the potential bias of this, and other instruments used to measure CU traits. Like other psychological tools, the ICU may be more sensitive to expressions of these traits that are more typical in boys. This may occur because many assessment scales were originally developed using predominantly male samples, which may limit their sensitivity in detecting how these traits manifest in girls.

Certain limitations should be noted in this study. As is common in systematic reviews, there is a risk of publication bias, as only studies published in identified sources were included. However, this risk was somewhat mitigated by additional manual searches. Another important point is the heterogeneity of the studies in the current review. Although the systematic review followed rigorous guidelines such as the PRISMA method, the diversity of samples and methodological variability across studies hinder the establishment of a solid basis for cross-cultural, age, and sex comparisons. This limitation is further compounded by the fact that some studies did not provide sufficient data for complete statistical analysis, resulting in their exclusion from the review.

Additionally, the study faced challenges in aggregating data across specific ICU versions, further limiting comparability between clinical and community samples. This is a critical issue, as the lack of a cohesive database hampers the establishment of widely applicable reference values, particularly in clinical contexts (e.g., developmental disorders). The lack of standardization across studies limits the generalizability of results and suggests the need for further research to consolidate proposed reference values.

Finally, there is a methodological issue regarding how the ICU is applied across different cultural contexts. Psychological assessment tools do not always effectively translate concepts across cultures, thereby affecting the instruments’ reliability and validity. Some cultural groups may struggle with negatively worded items or exhibit cultural preferences that shape their responses. These variations may compromise the assessment’s accuracy, and researchers must focus on developing appropriate cultural adaptations to ensure the ICU’s cross-cultural validity.

Despite the abovementioned limitations, establishing and providing reference values for the ICU is particularly important for drawing valid conclusions about the characteristics of children and adolescents assessed in clinical, community, and forensic settings, especially given the ICU is widespread use in these evaluative contexts. Moreover, the values provided in this study may serve as useful benchmarks for future research, particularly for comparative purposes.

## Supplemental Material


Supplemental Material - Reference Values of the Inventory of Callous-Unemotional Traits in Youth Populations: A Systematic Literature Review
Supplemental Material for Reference Values of the Inventory of Callous-Unemotional Traits in Youth Populations: A Systematic Literature Review by Patrícia Figueiredo, Beatriz Lopes, Melhyssa Gomes, Eduarda Ramião, and Fernando Barbosa in Clinical Child Psychology and Psychiatry.

Supplemental Material - Reference Values of the Inventory of Callous-Unemotional Traits in Youth Populations: A Systematic Literature Review
Supplemental Material for Reference Values of the Inventory of Callous-Unemotional Traits in Youth Populations: A Systematic Literature Review by Patrícia Figueiredo, Beatriz Lopes, Melhyssa Gomes, Eduarda Ramião, and Fernando Barbosa in Clinical Child Psychology and Psychiatry.

Supplemental Material - Reference Values of the Inventory of Callous-Unemotional Traits in Youth Populations: A Systematic Literature Review
Supplemental Material for Reference Values of the Inventory of Callous-Unemotional Traits in Youth Populations: A Systematic Literature Review by Patrícia Figueiredo, Beatriz Lopes, Melhyssa Gomes, Eduarda Ramião, and Fernando Barbosa in Clinical Child Psychology and Psychiatry.

## References

[bibr1-13591045261430966] Abrams L. S. (2013). Juvenile justice at a crossroads: Science, evidence, and twenty-first century reform. Social Service Review, 87(4), 725–752. 10.1086/674074 PMC12721960 41438479

[bibr2-13591045261430966] Achenbach T. M. Becker A. Döpfner M. Heiervang E. Roessner V. Steinhausen H. C. Rothenberger A. (2008). Multicultural assessment of child and adolescent psychopathology with ASEBA and SDQ instruments: Research findings, applications, and future directions. Journal of Child Psychology and Psychiatry, and Allied Disciplines, 49(3), 251–275. 10.1111/j.1469-7610.2007.01867.x 18333930

[bibr3-13591045261430966] Andershed H. Kerr M. Stattin H. Levander S. (2002). Youth psychopathic traits inventory (YPI). APA PsycTests.

[bibr4-13591045261430966] Anderson N. E. Kiehl K. A. (2014). Psychopathy: Developmental perspectives and their implications for treatment. Restorative Neurology and Neuroscience, 32(1), 103–117. 10.3233/rnn-139001 PMC4321752 23542910

[bibr5-13591045261430966] Barker E. D. Oliver B. R. Viding E. Salekin R. T. Maughan B. (2011). The impact of prenatal maternal risk, fearless temperament and early parenting on adolescent callous-unemotional traits: A 14-year longitudinal investigation. The Journal of Child Psychology and Psychiatry, 52(8), 878–888. 10.1111/j.1469-7610.2011.02397.x 21410472

[bibr6-13591045261430966] Barry C. T. Frick P. J. DeShazo T. M. McCoy M. Ellis M. Loney B. R. (2000). The importance of callous-unemotional traits for extending the concept of psychopathy to children. Journal of Abnormal Psychology, 109(2), 335–340. 10.1037/0021-843X.109.2.335 10895572

[bibr7-13591045261430966] Benesch C. Görtz-Dorten A. Breuer D. Döpfner M. (2014). Assessment of callous-unemotional traits in 6 to 12 year-old children with oppositional defiant disorder/conduct disorder by parent ratings. Journal of Psychopathology and Behavioral Assessment, 36(4), 519–529. 10.1007/s10862-014-9420-7

[bibr8-13591045261430966] Blair R. J. R. (1999). Responsiveness to distress cues in the child with psychopathic tendencies. Personality and Individual Differences, 27(1), 135–145. 10.1016/s0191-8869(98)00231-1

[bibr9-13591045261430966] Blair R. J. R. Colledge E. Murray L. Mitchell D. G. V. (2001). A selective impairment in the processing of sad and fearful expressions in children with psychopathic tendencies. Journal of Abnormal Child Psychology, 29(6), 491–498. 10.1023/a:1012225108281 11761283

[bibr10-13591045261430966] Borsboom D. (2006). When does measurement invariance matter? Medical Care, 44(11), S176–S181. 10.1097/01.mlr.0000245143.08679.cc 17060825

[bibr11-13591045261430966] Byrd A. L. Kahn R. E. Pardini D. A. (2013). A validation of the inventory of callous-unemotional traits in a community sample of young adult males. Journal of Psychopathology and Behavioral Assessment, 35(1), 20–34. 10.1007/s10862-012-9315-4 PMC3864815 24357894

[bibr12-13591045261430966] Camilo C. Garrido M. V. (2019). Systematic review in psychology: Challenges and guidelines. Análise Psicológica, 37(4), 535–552. 10.14417/ap.1546

[bibr13-13591045261430966] Cardoso A. R. Costa M. J. Sani A. I. Moreira D. (2023). Callous and unemotional traits as precursors to the development of female psychopathy. International Journal of Environmental Research and Public Health, 20(18), 6786. 10.3390/ijerph20186786 PMC10530721 37754645

[bibr14-13591045261430966] Carvalho M. Faria M. Conceição A. de Matos M. G. Essau C. (2018). Callous unemotional traits in children and adolescents. Psychometric properties of the Portuguese version of the inventory of callous-unemotional traits. European Journal of Psychological Assessment, 34(2), 101–110. 10.1027/1015-5759/a000449

[bibr15-13591045261430966] Chen C. Lee S.-y. Stevenson H. W. (1995). Response style and cross-cultural comparisons of rating scales among East Asian and North American students. Psychological Science, 6(3), 170–175. 10.1111/j.1467-9280.1995.tb00327.x

[bibr16-13591045261430966] Child Care & Early Education Research Connections . (2019). Quantitative research assessment tool. Retrieved from. https://www.researchconnections.org/childcare/datamethods/downloads/quantitativeresearch.pdf

[bibr17-13591045261430966] Cleckley H. (1976). The mask of sanity. C. V. Mosby. 10.1080/00325481.1951.11694097 14807904

[bibr18-13591045261430966] Colins O. F. Andershed H. Hellfeldt K. Fanti K. (2021). The incremental usefulness of teacher-rated psychopathic traits in 5- to 7-year olds in predicting teacher-, parent-, and child self-reported antisocial behavior at a six-year follow-up. Journal of Criminal Justice, 80, 101771. 10.1016/j.jcrimjus.2020.101771

[bibr19-13591045261430966] Colins O. F. Lopez-Romero L. Romero E. Andershed H. (2024). The prognostic usefulness of multiple specifiers for subtyping conduct problems in early childhood. Journal of the American Academy of Child & Adolescent Psychiatry, 63(4), 443–453. 10.1016/j.jaac.2023.05.022 37442204

[bibr20-13591045261430966] Cooke D. J. Hart S. D. Logan C. Michie C. (2012). Explicating the construct of psychopathy: Development and validation of a conceptual model, the comprehensive assessment of psychopathic personality (CAPP). International Journal of Forensic Mental Health, 11(4), 242–252. 10.1080/14999013.2012.746759

[bibr21-13591045261430966] Cooke R. Bewick B. M. Barkham M. Bradley M. Audin K. (2006). Measuring, monitoring and managing the psychological well-being of first year university students. British Journal of Guidance and Counselling, 34(4), 505–517. 10.1080/03069880600942624

[bibr22-13591045261430966] Donohue M. R. Hoyniak C. P. Tillman R. Barch D. M. Luby J. (2023). Associations of observed callous–unemotional behaviors in early childhood with conduct problems and substance use over 14 years. Development and Psychopathology, 35(1), 421–432. 10.1017/s0954579421000791 PMC10014890 36914291

[bibr23-13591045261430966] Essau C. A. Sasagawa S. Frick P. J. (2006). Callous-unemotional traits in a community sample of adolescents. Assessment, 13(4), 454–469. 10.1177/1073191106287354 17050915

[bibr24-13591045261430966] Ezpeleta L. de la Osa N. Granero R. Penelo E. Domènech J. M. (2013). Inventory of callous-unemotional traits in a community sample of preschoolers. Journal of Clinical Child & Adolescent Psychology, 42(1), 91–105. 10.1080/15374416.2012.734221 23095075

[bibr25-13591045261430966] Ezpeleta L. Navarro J. B. de la Osa N. Penelo E. Trepat E. Martin V. Domènech J. M. (2017). Attention to emotion through a go/no-go task in children with oppositionality and callous–unemotional traits. Comprehensive Psychiatry, 75, 35–45. 10.1016/j.comppsych.2017.02.004 28301801

[bibr26-13591045261430966] Fanti K. A. Frick P. J. Georgiou S. (2009). Linking callous-unemotional traits to instrumental and non-instrumental forms of aggression. Journal of Psychopathology and Behavioral Assessment, 31(4), 285–298. 10.1007/s10862-008-9111-3

[bibr27-13591045261430966] Fisher L. Blair R. J. (1998). Cognitive impairment and its relationship to psychopathic tendencies in children with emotional and behavioral difficulties. Journal of Abnormal Child Psychology, 26(6), 511–519. 10.1023/a:1022655919743 9915656

[bibr102-13591045261430966] Figueiredo, P., Moreira, D., Ramião, E., Barroso, R., & Barbosa, F. (2022). Psychometric properties of the Portuguese teacher-version of the Inventory of Callous-Unemotional Traits. Clinical Child Psyhcology and Psychiatry, 1-18. 10.1177/13591045211070168 35164538

[bibr101-13591045261430966] Figueiredo, P., Ramião, E., Moreira, D., Barroso, R., & Barbosa, F. (2023). Psychometric properties of the Portuguese teacher-version of the Inventory of Callous-Unemotional Traits in Preschoolers. Análise Psicológica, 41(1), 127–142. 10.14417/ap.1991 35164538

[bibr28-13591045261430966] Fontaine N. M. McCrory E. J. Boivin M. Moffitt T. E. Viding E. (2011). Predictors and outcomes of joint trajectories of callous–unemotional traits and conduct problems in childhood. Journal of Abnormal Psychology, 120(3), 730–742. 10.1037/a0022620 21341879

[bibr29-13591045261430966] Fontaine N. M. Rijsdijk F. V. McCrory E. J. Viding E. (2010). Etiology of different developmental trajectories of callous-unemotional traits. Journal of the American Academy of Child and Adolescent Psychiatry, 49(7), 656–664. 10.1016/j.jaac.2010.03.014 20610135

[bibr30-13591045261430966] Forth A. E. Kosson D. S. Hare R. D. (2003). The hare psychopathy checklist: Youth version. Multi-Health Systems.

[bibr100-13591045261430966] Frick, P. J., Cornell, A. H., Barry, C. T., Bodin, S. D., & Dane, H. E. (2003). Callous-unemotional traits and conduct problems in the prediction of conduct problem severity, aggression, and self-report of delinquency. Journal of abnormal child psychology, 31(4), 457–470. 10.1023/a:1023899703866 12831233

[bibr31-13591045261430966] Frick P. J. (2004). Inventory of callous–unemotional traits. PLoS One. 10.1037/t62639-000

[bibr32-13591045261430966] Frick P. J. Bodin S. D. Barry C. T. (2000). Psychopathic traits and conduct problems in community and clinic-referred samples of children: Further development of the psychopathy screening device. Psychological Assessment, 12(4), 382–393. 11147105

[bibr33-13591045261430966] Frick P. J. Ellis M. (1999). Callous-unemotional traits and subtypes of conduct disorder. Clinical Child and Family Psychology Review, 2(3), 149–168. 10.1023/a:1021803005547 11227072

[bibr34-13591045261430966] Frick P. J. Hare R. D. (2001). Antisocial process screening device. European Journal of Psychological Assessment. 10.1037/t00032-000

[bibr35-13591045261430966] Frick P. J. Ray J. V. Thornton L. C. Kahn R. E. (2014). Annual research review: A developmental psychopathology approach to understanding callous‐unemotional traits in children and adolescents with serious conduct problems. Journal of Child Psychology and Psychiatry, 55(6), 532–548. 10.1111/jcpp.12152 24117854

[bibr36-13591045261430966] Frick P. J. White S. F. (2008). Research review: The importance of callous-unemotional traits for developmental models of aggressive and antisocial behavior. Journal of Child Psychology and Psychiatry, and Allied Disciplines, 49(4), 359–375. 10.1111/j.1469-7610.2007.01862.x 18221345

[bibr37-13591045261430966] Gendreau P. Goggin C. Smith P. (2002). Is the PCL-R really the “unparalleled” measure of offender risk? A lesson in knowledge cumulation. Criminal Justice and Behavior, 29(4), 397–426. 10.1177/0093854802029004004

[bibr38-13591045261430966] Gendron M. (2017). Revisiting diversity: Cultural variation reveals the constructed nature of emotion perception. Current Opinion in Psychology, 17, 145–150. 10.1016/j.copsyc.2017.07.014 PMC5624526 28950961

[bibr39-13591045261430966] Hare R. (2003). Hare psychopathy checklist-revised. Multi-Health Systems.

[bibr40-13591045261430966] Harris M. N. Daigle L. E. Reidy D. Chafin T. Thomson N. D. (2024). Psychopathy checklist-youth version scores and recurring victimisation by an intimate partner in a justice-involved sample: A comparison of young men and women using longitudinal data. Criminal Behavior and Mental Health, 34(6), 477–493. 10.1002/cbm.2355 PMC11617798 39396239

[bibr41-13591045261430966] He J. van de Vijver F. (2012). Bias and equivalence in cross-cultural research. Online Readings in Psychology and Culture, 2(2). 10.9707/2307-0919.1111

[bibr42-13591045261430966] Houghton S. Hunter S. C. Crow J. (2013). Assessing callous unemotional traits in children aged 7-to 12-years: A confirmatory factor analysis of the inventory of callous unemotional traits. Journal of Psychopathology and Behavioral Assessment, 35(2), 215–222. 10.1007/s10862-012-9324-3

[bibr43-13591045261430966] Johnson M. M. Dismukes A. R. Vitacco M. J. Breiman C. Fleury D. Shirtcliff E. A. (2014). Psychopathy's influence on the coupling between hypothalamic–pituitary–adrenal and‐gonadal axes among incarcerated adolescents. Developmental Psychobiology, 56(3), 448–458. 10.1002/dev.21111 PMC5429590 23852424

[bibr45-13591045261430966] Kimonis E. R. Fanti K. A. Anastassiou-Hadjicharalambous X. Mertan B. Goulter N. Katsimicha E. (2015). Can callous-unemotional traits be reliably measured in preschoolers? Journal of Abnormal Child Psychology, 44(4), 625–638. 10.1007/s10802-015-0075-y 26344015

[bibr46-13591045261430966] Kimonis E. R. Frick P. J. Munoz L. C. Aucoin K. J. (2008). Callous-unemotional traits and the emotional processing of distress cues in detained boys: Testing the moderating role of aggression, exposure to community violence, and histories of abuse. Development and Psychopathology, 20(2), 569–589. 10.1017/s095457940800028x 18423095

[bibr47-13591045261430966] Kimonis E. R. Frick P. J. Skeem J. L. Marsee M. A. Cruise K. Munoz L. C. Aucoin K. J. Morris A. S. (2008). Assessing callous–unemotional traits in adolescent offenders: Validation of the inventory of callous–unemotional traits. International Journal of Law and Psychiatry, 31(3), 241–252. 10.1016/j.ijlp.2008.04.002 18514315

[bibr48-13591045261430966] Kohls G. Bernhard A. Fairchild G. Stadler C. Jansen L. De Brito S. A. Roessner V. Freitag C. M. Konrad K. Elster E. M. (2025). Psychopathy traits and their link to emotion recognition impairments in conduct disorder. JCPP Advances, e70055. 10.1002/jcv2.70055 PMC13260674 42291684

[bibr49-13591045261430966] Landis J. R. Koch G. G. (1977). The measurement of observer agreement for categorical data. Biometrics, 33(1), 159–174. 843571

[bibr50-13591045261430966] Lappi-Seppälä T. (2011). Explaining imprisonment in Europe. European Journal of Criminology, 8(4), 303–328. 10.1177/1477370811411459

[bibr51-13591045261430966] Lappi-Seppälä T. (2018). Youth justice and youth sanctions in four Nordic states. Denmark, Finland, Norway and Sweden. In Goldson B. (Ed.), Juvenile justice in Europe. Past, present and future (pp. 104–127). Routledge.

[bibr52-13591045261430966] López-Romero L. Salekin R. T. Romero E. Andershed H. Colins O. F. (2022). Psychopathic Personality Configurations in Early Childhood: A Response to Dvoskin et al. (2022). Journal of Personality Disorders, 36(3), 254–263. 10.1521/pedi.2022.36.3.254 35647773

[bibr53-13591045261430966] Loney B. R. Frick P. J. Clements C. B. Ellis M. L. Kerlin K. (2003). Callous-unemotional traits, impulsivity, and emotional processing in adolescents with antisocial behavior problems. Journal of Clinical Child & Adolescent Psychology, 32(1), 66–80. 10.1207/S15374424JCCP3201_07 12573933

[bibr54-13591045261430966] Lynam D. R. (1997). Childhood Psychopathy Scale (CPS). APA PsycTests. 10.1037/t08226-000

[bibr55-13591045261430966] Lynam D. R. Caspi A. Moffitt T. E. Raine A. Loeber R. Stouthamer-Loeber M. (2005). Adolescent psychopathy and the big five: Results from two samples. Journal of Abnormal Child Psychology, 33(4), 431–443. 10.1007/s10648-005-5724-0 16118990

[bibr56-13591045261430966] Lynam D. R. Charnigo R. Moffitt T. E. Raine A. Loeber R. Stouthamer-Loeber M. (2009). The stability of psychopathy across adolescence. Development and Psychopathology, 21(4), 1133–1153. 10.1017/S0954579409990083 PMC3335366 19825261

[bibr57-13591045261430966] Markus H. R. Kitayama S. (1991). Culture and the self: Implications for cognition, emotion, and motivation. Psychological Review, 98(2), 224–253. 10.1037/0033-295X.98.2.224

[bibr58-13591045261430966] Matlasz T. M. Frick P. J. Clark J. E. (2022). Understanding the social relationships of youth with callous-unemotional traits using peer nominations. Journal of Clinical Child & Adolescent Psychology, 51(4), 530–542. 10.1080/15374416.2020.1823847 33125288

[bibr59-13591045261430966] Mesquita B. Leu J. (2007). The cultural psychology of emotion. In Kitayama S. Cohen D. (Eds.), Handbook of cultural psychology (pp. 734–759). The Guilford Press.

[bibr60-13591045261430966] Michielsen P. J. S. Habra M. M. J. Endendijk J. J. Bouter D. C. Grootendorst-van Mil N. H. Hoogendijk W. J. G. Roza S. J. (2022). Callous-unemotional traits and anxiety in adolescents: A latent profile analysis to identify different types of antisocial behavior in a high-risk community sample. Child and Adolescent Psychiatry and Mental Health, 16(1), 58. 10.1186/s13034-022-00493-8 PMC9297635 35854316

[bibr61-13591045261430966] Muñoz L. C. Qualter P. Padgett G. (2011). Empathy and bullying: Exploring the influence of callous-unemotional traits. Child Psychiatry & Human Development, 42(2), 183–196. 10.1007/s10578-010-0206-1 20886285

[bibr62-13591045261430966] Ngo D. A. Neumann C. S. Maurer J. M. Harenski C. Kiehl K. A. (2024). Psychopathic traits in adult versus adolescent males: Measurement invariance across the PCL-R and PCL:YV. Behavioral Sciences, 14(8), 672. 10.3390/bs14080672 PMC11351526 39199068

[bibr63-13591045261430966] Obradović J. Pardini D. A. Long J. D. Loeber R. (2007). Measuring interpersonal callousness in boys from childhood to adolescence: An examination of longitudinal invariance and temporal stability. Journal of Clinical Child & Adolescent Psychology, 36(3), 276–292. 10.1080/15374410701441633 17658974

[bibr64-13591045261430966] Page M. J. McKenzie J. E. Bossuyt P. M. Boutron I. Hoffmann T. C. Mulrow C. D. Shamseer L. Tetzlaff J. M. Akl E. A. Brennan S. E. Chou R. Glanville J. Grimshaw J. M. Hróbjartsson A. Lalu M. M. Li T. Loder E. W. Mayo-Wilson E. McDonald S. Moher D. (2021). The PRISMA 2020 statement: An updated guideline for reporting systematic reviews. Systematic Reviews, 10(1), 89. 10.1186/s13643-021-01626-4 PMC8008539 33781348

[bibr65-13591045261430966] Paz Y. Perkins E. R. Colins O. Perlstein S. Wagner N. J. Hawes S. W. Byrd A. Viding E. Waller R. (2024). Evaluating the sensitivity to threat and affiliative reward (STAR) model in relation to the development of conduct problems and callous-unemotional traits across early adolescence. Journal of Child Psychology and Psychiatry, and Allied Disciplines, 65(10), 1327–1339. 10.1111/jcpp.13976 PMC11393184 38480986

[bibr66-13591045261430966] Pechorro P. Da Silva D. R. Rijo D. Gonçalves R. A. Andershed H. (2017a). Psychometric properties and measurement invariance of the youth psychopathic traits inventory-short version among Portuguese youth. Journal of Psychopathology and Behavioral Assessment, 39(3), 486–497. 10.1007/s10862-017-9597-7

[bibr67-13591045261430966] Pechorro P. Goncalves R. A. Hawes S. W. Ray J. V. (2018). Psychometric properties of two short versions of the inventory of callous–unemotional traits among incarcerated youth. Journal of Emotional and Behavioral Disorders, 26(4), 243–256. 10.1177/1063426617717940

[bibr68-13591045261430966] Pechorro P. Ray J. Barroso R. Maroco J. Gonçalves R. A. (2016). Validation of the inventory of callous-unemotional traits among a Portuguese sample of detained juvenile offenders. International Journal of Offender Therapy and Comparative Criminology, 60(3), 349–365. 10.1177/0306624X14551256 25274746

[bibr69-13591045261430966] Pechorro P. Ray J. Gonçalves R. Jesus S. (2017b). The inventory of callous-unemotional traits: Psychometric properties among referred and non-referred Portuguese female juveniles. International Journal of Law and Psychiatry, 54(0), 67–75. 10.1016/j.ijlp.2017.05.002 28535945

[bibr70-13591045261430966] Pisano S. Muratori P. Gorga C. Levantini V. Iuliano R. Catone G. Coppola G. Milone A. Masi G. (2017). Conduct disorders and psychopathy in children and adolescents: Aetiology, clinical presentation and treatment strategies of callous-unemotional traits. Italian Journal of Pediatrics, 43(1), 84. 10.1186/s13052-017-0404-6 PMC5607565 28931400

[bibr71-13591045261430966] Pratt J. Eriksson A. (2013). Contrasts in punishment: An explanation of Anglophone excess and Nordic exceptionalism. Routledge.

[bibr72-13591045261430966] Pueyo N. Navarro J.-B. de la Osa N. Pechorro P. Ezpeleta L. (2024). Callous-unemotional traits and pubertal development: Sex-specific effects on disruptive behavior in children at ages 11 and 12. Journal of Criminal Justice, 92, 102184. 10.1016/j.jcrimjus.2024.102184

[bibr73-13591045261430966] Rowe R. Maughan B. Moran P. Ford T. Briskman J. Goodman R. (2010). The role of callous and unemotional traits in the diagnosis of conduct disorder. Journal of Child Psychology and Psychiatry, and Allied Disciplines, 51(6), 688–695. 10.1111/j.1469-7610.2009.02199.x 20039995

[bibr74-13591045261430966] Ručević S. Andershed H. (2022). Are psychopathic traits predictive of conduct problems and aggression when other risk factors are considered? A longitudinal test among Croatian children. Journal of Criminal Justice, 80, 101777. 10.1016/j.jcrimjus.2020.101777

[bibr75-13591045261430966] Sakki H. St Clair M. C. Shou Y. Allen J. L. (2024). Punishment and reward sensitivity in risk-taking as potential mechanisms explaining the relationships between childhood callous-unemotional traits and adolescent substance use in a longitudinal cohort study sample. Research on Child and Adolescent Psychopathology, 53(1), 1–15. 10.1007/s10802-024-01255-0 PMC11761481 39441502

[bibr76-13591045261430966] Salekin R. T. (2017). Research review: What do we know about psychopathic traits in children? The Journal of Child Psychology and Psychiatry, 58(11), 1180–1200. 10.1111/jcpp.12738 28542927

[bibr77-13591045261430966] Salekin R. T. Andershed H. Batky B. D. Bontemps A. P. (2018). Are Callous Unemotional (CU) traits enough? Journal of Psychopathology and Behavioral Assessment, 40(1), 1–5. 10.1007/s10862-018-9663-9 PMC5860105 29576679

[bibr78-13591045261430966] Salekin R. T. Andershed H. Colins O. F. (2022). Introduction to the special section: What do we know about the psychophysiology of child psychopathy and conduct problems? Journal of Psychopathology and Behavioral Assessment, 44(1), 1–10. 10.1007/s10862-021-09950-8

[bibr79-13591045261430966] Salekin R. T. Bellamy N. A. DeGroot H. R. Avellan J. J. Butler I. G. Grant J. C. (2025). Future directions for conduct disorder and psychopathic trait specifiers. Journal of Clinical Child & Adolescent Psychology, 54(1), 163–179. 10.1080/15374416.2024.2447240 40036271

[bibr80-13591045261430966] Salekin R. T. Lynam D. R. (Eds.), (2011). Handbook of child and adolescent psychopathy. Guilford Press.

[bibr81-13591045261430966] Sawyer S. M. Azzopardi P. S. Wickremarathne D. Patton G. C. (2018). The age of adolescence. The Lancet Child & Adolescent Health, 2(3), 223–228. 10.1016/s2352-4642(18)30022-1

[bibr82-13591045261430966] Shou Y. Lay S. E. De Silva H. S. Xyrakis N. Sellbom M. (2019). Sociocultural influences on psychopathy traits: A cross-national investigation. Journal of Personality Disorders, 35(2), 194–216. 10.1521/pedi_2019_33_428 30920941

[bibr83-13591045261430966] Squillaci M. Benoit V. (2021). Role of callous and unemotional (CU) traits on the development of youth with behavioral disorders: A systematic review. International Journal of Environmental Research and Public Health, 18(9), 4712. 10.3390/ijerph18094712 PMC8125599 33925165

[bibr84-13591045261430966] Tonry M. Farrington D. P. (2005). Punishment and crime across space and time. University of Chicago Press.

[bibr85-13591045261430966] Vitacco M. J. Vincent G. M. (2006). Understanding the downward extension of psychopathy to youth: Implications for risk assessment and juvenile justice. International Journal of Forensic Mental Health, 5(1), 29–38. 10.1080/14999013.2006.10471228

[bibr86-13591045261430966] Waltz C. F. Strickland O. L. Lenz E. R. (2005). Measurement in nursing and health research (3rd ed.). Springer Publishing Company.

[bibr87-13591045261430966] Willoughby M. T. Mills-Koonce W. R. Waschbusch D. A. Gottfredson N. C. Family Life Project Investigators . (2015). An examination of the parent report version of the inventory of callous-unemotional traits in a community sample of first-grade children. Assessment, 22(1), 76–85. 10.1177/1073191114534886 PMC8011843 24820529

[bibr88-13591045261430966] Wright N. Hill J. Pickles A. Sharp H. (2019). Callous-Unemotional traits, low cortisol reactivity and physical aggression in children: Findings from the wirral child health and development study. Translational Psychiatry, 9(1), 79. 10.1038/s41398-019-0406-9 PMC6370839 30741941

[bibr89-13591045261430966] Zych I. Ttofi M. M. Farrington D. P. (2019). Empathy and callous–unemotional traits in different bullying roles: A systematic review and meta-analysis. Trauma, Violence, & Abuse, 20(1), 3–21. 10.1177/1524838016683456 30803395

