# Supplementary material for: Reference Values of the Inventory of Callous-Unemotional Traits in Youth Populations: A Systematic Literature Review
Source: Clin Child Psychol Psychiatry. 2026 Mar 9;31(4):1927–50. doi: 10.1177/13591045261430966 (PMC13620238; doi:10.1177/13591045261430966)
Supplement: Supplementary file 1 [file sj-pdf-1-ccp-10.1177_13591045261430966.pdf]

Table 1

Quality and risk bias of quantitative studies

Study ID	Item 1	Item 2	Item 3	Item 4	Item 5	Item 6	Item 7	Item 8	Item 9	Item 10	Item 11	Item 12	Total score
Ackermann et al. (2019)	1	0	1	1	1	1	1	1	1	1	1	1	11
Aggensteiner et al. (2022)	1	0	1	1	0	1	1	1	1	0	1	1	9
Aggensteiner et al. (2024)	1	0	1	1	1	1	1	1	1	1	1	1	11
Allen et al. (2016)	1	0	1	1	-1	1	1	1	1	0	1	1	7
Andershed et al. (2018)	1	0	1	1	1	1	1	1	1	0	1	1	10
Anderson et al. (2018)	1	0	1	1	1	1	1	1	1	0	1	1	10
Ansel et al. (2015)	1	0	1	1	1	1	1	1	1	0	1	1	10
Azzam et al. (2022)	1	0	1	1	-1	1	1	1	1	0	1	1	8
Bakker-Huvenaars et al. (2020)	1	0	1	1	1	1	1	1	1	0	1	1	10
Bansal et al. (2020)	1	0	1	NA	1	1	1	1	1	0	1	NA	9
Bansal et al. (2022)	1	0	1	NA	1	1	1	1	1	0	1	NA	9
Barhight et al. (2017)	1	0	1	1	0	1	1	1	1	0	1	1	9
Baroncelli et al. (2017)	1	0	1	1	1	1	1	1	1	0	1	1	10
Barroso et al. (2021)	1	0	1	1	1	1	1	1	1	1	1	1	11
Bell et al. (2018)	1	0	1	1	1	1	1	1	1	1	1	1	11
Benesch et al. (2014)	1	0	1	1	1	1	1	1	1	0	1	1	10
Bennett & Kering (2014)	1	0	1	1	1	1	1	1	1	0	1	1	10
Berg et al. (2013)	1	0	1	NA	0	1	1	1	1	0	1	1	8
Bhanwer et al. (2019)	1	0	1	1	0	1	1	1	1	0	1	1	9
Bird et al. (2019)	1	0	1	1	1	1	1	1	1	1	1	1	11
Bisby et al. (2017)	1	0	1	NA	1	1	1	1	1	0	1	1	9
Blair et al. (2020)	1	0	1	1	1	1	1	1	1	1	1	1	11
Blair et al. (2021)	1	0	1	NA	1	1	1	1	1	1	1	1	10
Blanchard et al. (2017)	1	0	1	1	0	1	1	1	1	0	1	1	9

Bonham et al. (2022)	1	1	1	1	0	1	1	1	1	1	1	1	11
Böttinger et al. (2024)	1	1	1	1	0	1	1	1	1	0	1	1	10
Bours et al. (2018)	1	1	1	1	1	1	1	1	1	0	1	1	11
Breaux et al. (2020)	1	1	1	1	1	1	1	1	1	1	1	1	12
Cao et al. (2024)	1	1	1	1	0	1	1	1	1	1	1	1	11
Cardinale et al. (2018)	1	1	1	1	1	1	1	1	1	1	0	1	11
Cardinale et al. (2019)	1	1	1	1	1	1	1	1	1	1	1	1	12
Carvalho et al. (2018)	1	1	1	1	1	1	1	1	1	1	1	1	12
Castagna et al. (2021a)	1	1	1	1	1	1	1	1	1	1	1	1	12
Castagna et al. (2021b)	1	1	1	1	1	1	1	1	1	1	1	1	12
Castagna et al. (2025)	1	1	1	1	1	1	1	1	1	1	1	1	12
Cecil et al. (2018)	1	1	1	1	1	1	1	1	1	0	1	1	11
Chang et al. (2021)	1	1	1	1	1	1	1	1	1	1	1	1	12
Charles et al. (2012)	1	1	1	1	0	1	1	1	1	0	1	1	10
Ciucci et al. (2014)	1	1	1	1	1	1	1	1	1	1	1	1	12
Ciucci et al. (2014)	1	1	1	1	1	1	1	1	1	0	1	1	11
Ciucci et al. (2018)	1	1	1	1	1	1	1	1	1	0	1	1	11
Ciucci et al. (2024)	1	1	1	1	1	1	1	1	1	1	1	1	12
Clark & Frick (2018)	1	1	1	1	0	1	1	1	1	1	1	1	11
Coe et al. (2018)	1	1	1	1	1	1	1	1	1	1	1	1	12
Colins et al. (2016)	1	1	1	1	1	1	1	1	1	0	1	1	11
Colins et al. (2018)	1	1	1	1	1	1	1	1	1	1	1	1	12
Corbelli et al. (2024)	1	1	1	1	0	1	1	1	1	1	1	1	11
Craig & Moretti (2019)	1	1	1	1	1	1	1	1	1	0	1	1	11
Craig et al. (2019)	1	1	1	1	0	1	1	1	1	0	1	1	10
Dandreaux & Frick (2009)	1	1	1	1	0	1	1	1	1	0	1	1	10
Dapprich et al. (2022)	1	1	1	1	1	1	1	1	1	1	1	1	12
Dapprich et al. (2024)	1	1	1	1	1	1	1	1	1	1	1	1	12
Dargis & Li (2020)	1	1	1	1	1	1	1	1	1	1	1	1	12
Darvis et al. (2015)	1	1	1	0	0	1	1	1	1	0	0	1	8
De Brito et al. (2025)	1	1	1	0	1	1	1	1	1	1	1	1	11
DeLisi et al. (2011)	1	1	1	0	1	1	1	1	1	0	1	1	10
Derks et al. (2024a)	1	1	1	1	1	1	1	1	1	1	1	1	12
Derks et al. (2024a)	1	1	1	0	1	1	1	1	1	1	1	1	11
Docherty et al. (2016)	1	1	1	0	0	1	1	1	1	0	1	1	9

Drent et al. (2010)	1	1	1	1	1	1	1	1	1	1	1	1	12
Dugré et al. (2023)	1	1	1	1	1	1	1	1	1	1	1	1	12
Duindam et al. (2021)	1	1	1	1	0	1	1	1	1	1	1	1	11
Eisenbarth et al. (2016)	1	1	1	1	1	1	1	1	1	1	1	1	12
Elizur et al. (2017)	1	1	1	1	1	1	1	1	1	1	1	1	12
Elowsky et al. (2022)	1	1	1	1	1	1	1	1	1	1	1	1	12
Essau (2006)	1	1	1	0	1	1	1	1	1	0	1	1	10
Euler et al. (2014)	1	1	1	1	0	1	1	1	1	0	1	1	10
Everett et al. (2023)	1	1	1	1	0	1	1	1	1	1	1	1	11
Ezpeleta et al. (2013)	1	1	1	1	1	1	1	1	1	1	1	1	12
Ezpeleta et al. (2015)	1	1	1	1	1	1	1	1	1	1	1	1	12
Ezpeleta et al. (2017)	1	1	1	1	1	1	1	1	1	1	1	1	12
Ezpeleta et al. (2019)	1	1	1	1	0	1	1	1	1	1	1	1	11
Facci et al. (2019)	1	1	1	1	0	1	1	1	1	0	1	1	10
Fagan et al. (2017)	1	1	1	1	0	1	1	1	1	0	1	1	10
Fairchild et al. (2014)	1	1	1	1	0	1	1	1	1	1	1	1	11
Fang et al. (2020)	1	1	1	1	1	1	1	1	1	1	1	1	12
Fang et al. (2022)	1	1	1	1	1	1	1	1	1	1	1	1	12
Fanniff & Kimonis (2014)	1	1	1	1	0	1	1	1	1	0	1	1	10
Fanti & Kimonis (2012)	1	1	1	1	0	1	1	1	1	0	1	1	10
Fanti & Kimonis (2013)	1	1	1	1	1	1	1	1	1	1	1	1	12
Fanti et al. (2009)	1	1	1	1	0	1	1	1	1	0	1	1	11
Fanti et al. (2013a)	1	1	1	1	1	1	1	1	1	1	1	1	12
Fanti et al. (2013b)	1	1	1	1	1	1	1	1	1	1	1	1	12
Fanti et al. (2017)	1	1	1	1	1	1	1	1	1	1	1	1	12
Fanti et al. (2018)	1	1	1	1	1	1	1	1	1	1	1	1	12
Fanti et al (2019)	1	1	1	1	1	1	1	1	1	0	1	1	11
Feilhauer et al. (2012)	1	1	1	1	0	1	1	1	1	0	1	1	10
Feilhauer et al. (2013a)	1	1	1	1	0	1	1	1	1	0	1	1	10
Feilhauer et al. (2013b)	1	1	1	1	0	1	1	1	1	0	1	1	10
Figueiredo et al. (2022)	1	1	1	1	1	1	1	1	1	1	1	1	12
Figueiredo et al.	1	1	1	1	1	1	1	1	1	1	1	1	12

(2023)													
Fine et al. (2017)	1	1	1	1	1	1	1	1	1	1	1	1	12
Fink et al. (2012)	1	1	1	1	1	1	0	1	1	0	1	1	10
Fisher & Brown (2018)	1	1	1	1	0	1	1	1	1	0	1	1	10
Fite et al. (2018)	1	1	1	1	1	1	1	1	1	1	1	1	12
Fite et al. (2020)	1	1	1	1	1	1	1	1	1	1	1	1	12
Fite et al. (2019)	1	1	1	1	1	1	1	1	1	1	1	1	12
Fleming et al. (2022)	1	1	1	1	0	1	1	1	1	1	1	1	11
Fragkaki et al. (2016)	1	1	1	1	1	1	1	1	1	0	1	1	11
Fragkaki et al. (2019a)	1	1	1	1	0	1	1	1	1	1	1	1	11
Fragkaki et al. (2019b)	1	1	1	1	0	1	1	1	1	1	1	1	11
Fragkaki & Cima (2019)	1	1	1	1	0	1	1	1	1	0	1	1	10
Gao & Raine (2024)													
Gao & Zhang (2016)	1	1	1	1	1	1	0	1	1	0	1	1	10
Gao & Zhang (2021)													
Gao et al. (2017)	1	1	1	1	0	1	1	1	1	0	1	1	10
Gaule et al. (2024a)	1	1	1	1	0	1	1	1	1	1	0	1	10
Gaule et al. (2024b)	1	1	1	1	0	1	1	1	1	1	0	1	10
Georgiou et al. (2019)	1	1	1	1	0	1	1	1	1	0	1	1	10
Gillen et al. (2019)	1	1	1	1	0	1	1	1	1	0	1	1	10
Gluckman et al (2016)	1	1	1	1	0	1	1	1	1	0	1	1	10
Goagoses & Koglin (2021)	1	1	1	1	0	1	1	1	1	1	0	1	10
Goagoses et al. (2022)	1	1	1	1	0	1	1	1	1	1	0	1	10
Goffin et al (2017)													
Goffin et al. (2018)	1	1	1	1	0	1	1	1	1	0	1	1	10
Gong et al. (2022)	1	1	1	1	0	1	1	1	1	0	1	1	10
González-Madruga et al (2019)	1	1	1	1	1	1	1	1	1	1	0	1	11
Grassetti et al. (2023)	1	1	1	1	0	1	1	1	1	1	0	1	10
Graziano et al. (2022a)	1	1	1	1	0	1	1	1	1	0	1	1	10
Grotzinger et al. (2018)	1	1	1	1	1	1	1	1	1	1	1	1	11
Guelker et al. (2014)	1	1	1	1	0	1	1	1	1	0	1	1	10
Harenski et al. (2014)	1	1	1	1	0	1	1	1	1	0	1	1	10

Hawes et al. (2014)	1	1	1	1	1	1	1	1	1	0	0	1	10
He et al. (2024)	1	1	1	1	0	1	1	1	1	1	0	1	10
Herpers et al. (2016)	1	1	1	1	0	1	1	1	1	1	0	1	10
Herpers et al (2019)	1	1	1	1	0	1	1	1	1	0	1	1	10
Heynen et al. (2017)	1	1	1	1	1	1	1	1	1	1	0	1	11
Hitti et al. (2021)	1	1	1	1	1	1	1	1	1	1	0	1	11
Hodsoll et al. (2014)	1	1	1	1	0	1	1	1	1	0	1	1	10
Hostetler et al. (2024)	1	1	1	1	1	1	1	1	1	1	1	1	11
Howard et al. (2012)	1	1	1	1	0	1	1	1	1	0	1	1	10
Hoyniak et al. (2018)	1	1	1	1	1	1	1	1	1	1	0	1	11
Hulvershorn et al. (2013)	1	1	1	1	0	1	1	1	1	0	1	1	10
Hwang et al. (2016)	1	1	1	1	0	1	1	1	1	0	1	1	10
Hwang et al. (2021)	1	1	1	1	1	1	1	1	1	1	0	1	11
Johander et al. (2022)	1	1	1	1	0	1	1	1	1	1	0	1	10
Johnson et al. (2014)	1	1	1	1	0	1	1	1	1	0	1	1	10
Kahn et al. (2016)	1	1	1	1	0	1	1	1	1	0	1	1	10
Kahn et al. (2017)	1	1	1	1	1	1	1	1	1	0	1	1	11
Kaouar et al. (2024)	1	1	1	1	0	1	1	1	1	1	0	1	10
Kauten et al. (2015)	1	1	1	1	0	1	1	1	1	0	1	1	10
Kavish et al. (2017)	1	1	1	1	0	1	1	1	1	0	1	1	10
Kemp et al. (2022)													
Kemp et al. (2023)	1	1	1	1	1	1	1	1	1	1	0	1	11
Kemp et al. (2024)	1	1	1	1	1	1	1	1	1	1	0	1	11
Kerig et al. (2012)	1	1	1	1	0	1	1	1	1	0	1	1	10
Kerig & Modrowski (2018)	1	1	1	1	1	1	1	1	1	1	0	1	11
Kilshaw et al. (2024)	1	1	1	1	0	1	1	1	1	1	0	1	10
Kimonis et al. (2007)	1	1	1	1	1	1	1	1	1	1	0	1	11
Al et al. (2008)	1	1	1	1	0	1	1	1	1	0	1	1	10
Kimonis et al. (2011)	1	1	1	1	0	1	1	1	1	0	1	1	10
Kimonis et al. (2013)	1	1	1	1	0	1	1	1	1	0	1	1	10
Kimonis et al. (2016)	1	1	1	1	0	1	1	1	1	1	0	1	10
Kimonis et al. (2017)	1	1	1	1	0	1	1	1	1	1	0	1	10
Kimonis et al. (2018)	1	1	1	1	1	1	1	1	1	1	0	1	11

Kimonis et al. (2019a)	1	1	1	1	0	1	1	1	1	1	0	1	10
Kimonis et al. (2019b)	1	1	1	1	0	1	1	1	1	0	1	1	10
Kimonis et al. (2023)	1	1	1	1	1	1	1	1	1	1	0	1	11
Kimonis et al. (2025)	1	1	1	1	1	1	1	1	1	1	0	1	11
Kleberg et al. (2021)	1	1	1	1	0	1	1	1	1	1	0	1	10
Kochanska et al. (2013)	1	1	1	1	1	1	1	1	1	1	1	1	12
Kofler et al. (2022)	1	1	1	1	0	1	1	1	1	0	1	1	10
Koh et al. (2024)	1	1	1	1	0	1	1	1	1	0	0	1	9
Kongerslev et al (2015)	1	1	1	1	0	1	1	1	1	0	1	1	10
Kumsta et al. (2012)	1	1	1	1	1	1	1	1	1	1	1	1	12
Kurek et al. (2019)	1	1	1	1	1	1	1	1	1	0	0	1	10
Kyranides et al. (2018)	1	1	1	1	1	1	1	1	1	1	1	1	12
Lam et al. (2021)	1	1	1	1	-1	1	1	1	1	0	0	1	8
Latzman et al. (2016)	1	1	1	1	0	1	1	1	1	0	1	1	10
Lau & Marsee (2013)	1	1	1	1	0	1	1	1	1	0	1	1	10
Lee-Rowland et al. (2020)	1	1	1	1	0	1	1	1	1	0	0	1	9
Levy et al. (2022)	1	1	1	1	0	1	1	1	1	0	0	1	9
Liu & Guo (2024)	1	1	1	1	0	1	1	1	1	0	1	1	10
Lopez-Romero et al. (2015a)	1	1	1	1	1	1	1	1	1	1	1	1	12
Lopez-Romero et al. (2015b)	1	1	1	1	0	1	1	1	1	1	0	1	10
Lorber et al. (2011)	1	0	1	NA	0	1	1	1	1	0	0	1	7
Lozier et al. (2014)	1	1	1	1	-1	1	1	1	1	1	0	1	9
Lui et al. (2016)													
Lui et al. (2017)	1	1	1	1	0	1	1	1	1	0	0	1	9
Lui et al. (2019)													
Lynch et al. (2024)	1	1	1	0	1	NA	1	1	0	NA	1	0	7
Ma et al. (2024)	1	1	0	0	1	NA	1	1	0	1	1	0	7
Maneiro et al. (2019)	1	1	1	1	1	0	1	1	0	1	1	1	10
Mann et al. (2018)	1	1	1	1	1	1	1	1	1	0	1	1	11
Marsee et al. (2014b)	1	1	1	1	1	0	1	1	0	0	1	1	7
Marsee et al. (2014a)													

Martin-Key et al. (2020)	1	1	1	1	1	1	1	1	1	NA	1	0	10
Martin-Key et al. (2021)	1	1	1	1	1	1	1	1	1	NA	1	0	10
Masi et al. (2011)	1	1	1	1	1	1	1	1	0	1	1	1	11
Masi et al. (2018)													
Matlasz et al. (2020)	1	1	1	1	0	0	1	1	1	0	1	1	9
Matlasz et al. (2023)	1	1	1	1	0	0	1	1	1	0	1	1	9
Mattos et al. (2017)	1	1	1	1	0	0	1	1	0	0	1	0	7
McLoughlin et al. (2010)	1	1	1	0	0	0	1	1	0	0	0	0	5
Mendez et al. (2020)	1	1	1	1	1	0	1	1	1	1	1	1	11
Milledge et al. (2019)	1	1	1	1	0	0	1	1	1	0	1	1	9
Miller et al. (2013)	1	0	1	1	-1	0	1	1	0	0	1	1	6
Miller & Marsee (2019)	1	1	1	1	0	0	1	1	1	1	1	1	10
Mozley et al. (2018)	1	1	1	1	1	0	1	1	1	1	1	1	11
Muñoz et al. (2008a)	1	1	1	1	0	0	1	1	1	0	1	1	9
Muñoz et al. (2008b)	1	1	1	1	0	0	1	1	1	0	1	1	9
Muñoz (2009)	1	1	1	1	0	0	1	1	1	0	1	1	9
Muñoz et al. (2011)	1	1	1	1	1	0	1	1	1	0	1	1	10
Muratori et al. (2016)	1	1	1	1	1	0	1	1	0	0	1	0	8
Muratori et al. (2019)	1	1	1	1	1	NA	1	1	1	NA	1	0	9
Muratori et al. (2021)	1	1	1	1	1	NA	1	1	1	NA	1	0	9
Myers et al. (2018)	1	1	1	1	1	1	1	1	1	1	1	1	12
Nichols et al. (2015)	1	1	1	1	1	1	1	1	1	1	1	1	12
Normand et al (2023)	1	1	1	1	1	1	1	1	1	0	1	1	11
Nwafor et al. (2015)	1	1	1	0	0	NA	1	1	0	NA	1	0	6
Paciello et al. (2017)	1	1	1	1	1	NA	1	1	1	NA	1	0	9
Payot et al. (2025)	1	1	1	1	1	1	1	1	1	0	1	1	11
Pechorro et al. (2018)	1	1	1	1	1	NA	1	1	1	NA	1	1	10
Pechorro et al. (2019)	1	1	1	1	1	NA	1	1	1	NA	1	1	10
Pederson et al. (2020)	1	1	1	1	1	NA	1	1	0	NA	1	0	8
Perlstein et al. (2021)	1	1	1	1	1	1	1	1	1	1	1	1	12
Pihet et al. (2015)	1	1	1	1	1	1	1	1	1	1	1	1	12
Piscitello et al. (2025)	1	1	1	1	0	0	1	1	0	0	1	0	7

Platje et al. (2018)	1	1	1	1	0	NA	1	1	1	0	1	1	9
Portengen et al. (2021)	1	1	1	1	1	NA	1	1	1	1	0	1	10
Powell et al. (2024)	1	1	1	1	0	1	1	1	1	1	1	1	11
Preston et al. (2021)	1	1	1	1	1	1	1	1	1	0	1	1	
Priddis et al. (2014) Australia	1	1	1	1	0	0	1	1	0	0	1	0	
Puzzo et al. (2018)	1	1	1	1	0	NA	1	1	1	NA	1	1	11
Rehder et al. (2017)	1	1	1	1	1	NA	1	1	1	NA	1	1	7
Rehder et al. (2021)	1	1	1	1	1	NA	1	1	1	NA	1	1	9
Rhoads et al. (2020)	1	1	1	1	0	NA	1	1	1	NA	1	1	10
Rizeq et al. (2020)	1	1	1	1	0	NA	1	1	1	NA	1	1	10
Roberts et al. (2018)	1	1	1	1	0	NA	1	1	0	NA	1	1	9
Roberts et al. (2020)	1	1	1	1	0	NA	1	1	1	NA	1	1	9
Robertson et al. (2020)													8
Robertson et al. (2021)	1	1	1	1	1	NA	1	1	1	1	1	1	11
Romero & Alonso (2017)	1	1	1	1	0	NA	1	1	1	NA	1	1	9
Roose et al. (2013)	1	1	1	1	1	0	1	1	1	1	1	1	11
Sanchez & Cooley (2024)	1	1	1	1	1	1	1	1	1	1	1	1	12
Scavenius et al. (2020)	1	1	1	1	0	NA	1	1	0	NA	1	0	7
Schwenck et al (2017)	1	1	1	1	0	NA	1	1	1	NA	1	1	9
Sebastian et al. (2014)	1	1	1	1	1	NA	1	1	1	NA	1	1	10
Sebastian et al. (2016)	1	1	1	1	1	NA	1	1	1	NA	1	1	10
Sharf et al. (2014)	1	1	1	1	0	NA	1	1	1	NA	1	0	8
Sharp & Vanwoerden (2014)	1	1	1	1	1	0	1	1	1	1	1	1	11
Simpson et al. (2013)	1	1	1	1	0	NA	1	1	0	NA	1	0	7
Sourander et al. (2016)	1	1	1	1	1	1	1	1	1	1	1	1	12
Speck et al. (2024)	1	1	1	1	1	NA	1	1	1	NA	1	1	10
Stickle et al. (2009)	1	1	1	1	0	NA	1	1	0	NA	1	0	7
Stoppelbein et al. (2021)	1	1	1	1	1	1	1	1	1	1	1	1	12
Sukhodolsky et al. (2022)	1	1	1	1	1	NA	1	1	1	NA	1	1	10
Szabó et al. (2020)	1	1	1	1	0	NA	1	1	1	NA	1	0	8

Tabares et al. (2025)	1	1	1	1	1	1	1	1	1	1	1	1	12
Tan et al. (2025)	1	1	1	1	1	1	1	1	1	1	1	1	12
Thogersen et al. (2021)	1	1	1	1	1	0	1	1	1	0	1	1	10
Thomson et al. (2024)													
Thornberg et al. (2017)	1	1	1	1	0	NA	1	1	1	NA	1	1	9
Thornberg et al. (2025)	1	1	1	1	1	NA	1	1	1	NA	1	1	10
Thornton et al. (2019)	1	1	1	1	1	1	1	1	1	1	1	1	12
Truedsson et al. (2019)	1	1	1	1	0	NA	1	1	1	NA	1	1	9
Urben et al. (2018)	1	1	1	1	1	NA	1	1	1	NA	1	1	10
Vaughn et al. (2011)	1	1	1	1	0	NA	1	1	0	NA	1	0	7
Vaughan et al. (2025)	1	1	1	1	1	1	1	1	1	1	1	1	12
Veroude et al (2016)	1	1	1	1	1	1	1	1	1	1	1	1	12
Viding et al. (2012)	1	1	1	1	1	NA	1	1	1	NA	1	1	10
Wade et al. (2022)	1	1	1	1	1	1	1	1	1	1	1	1	12
Wagner et al. (2015)	1	1	1	1	1	NA	1	1	1	NA	1	1	10
Walker et al. (2020)	1	1	1	0	0	NA	1	1	1	0	1	1	8
Wallace et al. (2014)	1	1	1	0	−1	NA	1	1	0	0	1	1	6
Waller et al. (2016)													
Walsh et al. (2019)	1	1	1	1	0	NA	1	1	1	1	1	1	10
Wang & Zhou (2023)	1	1	1	1	0	NA	1	1	1	0	1	1	9
Waschbusch et al. (2020)	1	1	1	1	1	1	1	1	1	1	1	1	12
Waschbusch et al. (2024a)	1	1	1	1	1	1	1	1	1	1	1	1	12
Waschbusch et al. (2024b)	1	1	1	1	1	1	1	1	1	1	1	1	12
Waxmonsky et al. (2022)	1	1	1	1	0	1	1	1	1	0	1	1	10
Werhahn et al. (2021)	1	1	1	1	0	NA	1	1	1	0	1	1	9
White et al. (2013)	1	1	1	1	0	NA	1	1	1	0	1	1	9
White et al. (2013a)	1	1	1	1	0	NA	1	1	1	0	1	1	9
White et al. (2014)	1	1	1	1	0	NA	1	1	1	0	1	1	9
White et al. (2016)	1	1	1	1	1	NA	1	1	1	NA	1	1	10
Wilke & Goagoses	1	1	1	1	1	NA	1	1	1	NA	1	1	10

(2023)													
Wilkinson & Jones (2021)	1	1	1	1	0	NA	1	1	1	0	1	1	9
Winebrake et al. (2024)	1	1	1	1	1	0	1	1	1	1	1	1	11
Winstanley et al. (2021)	1	1	1	1	1	1	1	1	1	1	1	1	12
Winters et al. (2023)	1	1	1	1	1	1	1	1	1	1	1	1	12
Winters et al. (2025)	1	1	1	1	1	1	1	1	1	1	1	1	12
Winters & Sakai (2023)	1	1	1	1	1	1	1	1	1	1	1	1	12
Wright et al. (2019)	1	1	1	1	0	NA	1	1	1	0	1	1	9
Xie et al. (2020)	1	1	1	1	0	NA	1	1	1	0	1	1	9
Xu et al. (2022a)	1	1	1	1	1	NA	1	1	1	NA	1	1	10
Xu et al. (2022b)	1	1	1	1	1	NA	1	1	1	NA	1	1	10
Yang et al. (2021)	1	1	1	1	1	NA	1	1	1	NA	1	1	10
Yang et al. (2024)													
Zhang et al. (2021a)	1	1	1	1	0	NA	1	1	1	0	1	1	9
Zhang et al. (2021b)	1	1	1	1	1	1	1	1	1	1	1	1	12
Zhong et al. (2020)	1	1	1	1	1	1	1	1	1	1	1	1	12
Zumbach et al. (2021)	1	1	1	1	1	1	1	1	1	1	1	1	12

Item 1 – Population; Item 2 Sample size; Item 3 - Randomized Selection of Participants; Item 4 - Response and Attrition Rate; Item 5 - Main Variables or Concepts; Item 6 - Operationalization of Concepts; Item 7 -Numeric Tables; Item 8 – Missing Data; Item 9 – Appropriatness of Statistical Tecnhiques; Item 10 – Omitted Variable Bias; Item 11 – Analysis of main Effect Variables; Item 12 – Ethical Approval.
