# Supplementary material for: Reference Values of the Inventory of Callous-Unemotional Traits in Youth Populations: A Systematic Literature Review
Source: Clin Child Psychol Psychiatry. 2026 Mar 9;31(4):1927–50. doi: 10.1177/13591045261430966 (PMC13620238; doi:10.1177/13591045261430966)
Supplement: Supplementary file 2 [file sj-pdf-2-ccp-10.1177_13591045261430966.pdf]

Supplementary Table

Summary of studies characteristics and main results

		Factor structure			Experimental Group				Control Group			
**Study, Country**	**Population**	**ICU version**	**Likert scale**	**Items by factors**	**% male**	**N**	**Age**	**Results**	**% male**	**n**	**Age**	**Results**
Ackermann et al. (2019) Germany	Clinic	ICU - self-report	0-3	Total: 24 items	45.9%	*n* = 115	9-18 years old	DBD Girls Total ICU: *M* = 25.98, *SD* = 9.68; DBD boys Total ICU: *M* = 28.67, *SD* = 3.99;	43.8%	*n* = 146	9-18 years old	No DBD girls Total ICU: *M* = 18.01, *SD* = 6.85; No DBD boys Total ICU: *M* = 21.30, *SD* = 6.19;
Aggenstein er et al. (2022) Netherlands	Clinic	ICU – parent version	0-3	Total: 24 items; Callous: 11 items; Unemotional: 5 items; Uncaring: 8 items	CD = 66.7%	CD: *n* = 47	8-18 years old	Total ICU: *M* = 34.19, *SD* = 9.52; Callous: *M* = 9.89, *SD* = 5.29; Uncaring: *M* = 16.57, *SD* = 4.91; Unemotional: *M* = 6.32, *SD* = 3.38	85.4%	*n* = 15	8-18 years old	Total ICU: *M* = 23.87, *SD* = 7.86; Callous: *M* = 3.8, *SD* = 3.70; Uncaring: *M* = 13.47, *SD* = 4.22; Unemotional: *M* = 5.87, *SD* = 2.85

Aggenstein er et al. (2024) Germany	Clinic	ICU – parent version	0-3	Total: 24 items; Callous: 11 items; Unemotional: 5 items; Uncaring: 8 items	CD and ODD = 94%	CD and ODD: *n* = 37	8-14 years old	Treatment 1 Total ICU: *M* = 33.20, *SD* = 9.80; Treatment 2 Total ICU: *M* = 33.11, *SD* = 8.85;	---	---	---	---
Allen et al. (2016) London	Communit y (without diagnosis)	ICU - self-report and teacher version	0-3	Total: 24 items; Callous: 11 items; Unemotional: 5 items; Uncaring: 8 items	100%	*n* = 39	12-13 years old	Self-report Total ICU: *M* = 25.44, *SD* = 8.67; Teacher version Total ICU: *M* = 26.15, *SD* = 12.96	---	---	---	---
Andershed et al. (2018) Cyprus	Communit y (without diagnosis)	ICU - self-report	0-3	Total: 24 items	48.0%	*n* = 996	12.12 years old	Boys Total ICU: *M* = 22.77, *SD* = 8.54; Girls Total ICU: *M* = 19.12, *SD* = 7.86	---	---	---	---
Anderson et al. (2024) Australia	Forensic	ICU - self-report	0-3	Total: 22 items;	100%	*n* = 130	13-20 years old	Total ICU: *M* = 23.55, *SD* = 8.06;	---	---	---	---
Ansel et al. (2015) USA	Communit y	ICU - self-report	0-3	Total: 24 items; Callous: 11 items; Unemotional: 5 items; Uncaring: 8 items	88.2%	*n* = 279	16-18 years old	Total ICU: *M* = 26.56, *SD* = 9.00; Callous: *M* = 8.58, *SD* = 4.32; Uncaring: *M* = 10.19, SD = 4.65; Unemotional: *M* = 7.96, *SD* = 3.08	---	---	---	---
Azzam et al. (2022) Egypt	Clinic	ICU-self-report and parent version	0-3	Total: 24 items;	75.0%	CD: *n* = 20	12-18 years old	Parent report CD Total ICU: *M* = 34.70, *SD* = 8.10; Self-report CD	65.0%	*n* = 20	12-18 years old	Parent report Total ICU: *M* = 26.70, *SD* = 1.20; Self-report Total ICU: *M* = 26.60, *SD* = 2.40

								Total ICU: *M* = 36.40, *SD* = 4.60				
Bakker-Huvenaars et al (2020) Netherlands	Clinic	ICU - self-report and parent version	0-3	Total: 24 items	100%	ODD/CD: *n* = 37	12-19 years old	Self-report<br>Total ICU: *M* = 31.20, *SD* = 9.40;<br>Parent report<br>Total ICU: *M* = 37.90, *SD* = 9.70	---	---	---	---
Bansal et al. (2020) USA	Community	ICU - self-report	0-3	Total: 24 items; Callous: 11 items Unemotional: 5 items Uncaring: 8 items	58.0%	*n* = 104	3-6 years	Total ICU: *M* = 19.79, *SD* = 10.67;<br>Callous: *M* = 6.38, *SD* = 4.93;<br>Unemotional: *M* = 3.20, *SD* = 2.53;<br>Uncaring: *M* = 10.22, *SD* = 5.16	---	---	---	---
Bansal et al. (2022) USA	Community	ICU - self-report	0-3	Total: 24 items; Callous: 11 items; Unemotional: 5 items; Uncaring: 8 items	51.7%	*n* = 1064	5-12 years	Total ICU: *M* = 14.16, *SD* = 17.28	---	---	---	---
Barhight et al. (2017a) USA	Community	ICU - self-report	0-3	Total: 22 items	46.0%	*n* = 771	*Mean* = 9.50-12.50 years	Total ICU: *M* = 39.82, *SD* = 8.14	---	---	---	---
Barhight et al. (2017b) USA	Community	ICU - self-report, parente and teacher versions	0-3	Total: 24 items	46.2%	*n* = 771	*Mean* = 10.71 years	Self-report<br>Total ICU: *M* = 26.16, *SD* = 6.00;<br>Parent report<br>Total ICU: *M* = 38.16, *SD* = 7.44<br>Teacher report<br>Total ICU: *M* = 42.72, *SD* = 12.48	---	---	---	---
Baroncelli et al (2018) Italy	Community	ICU - self-report	0-3	Total: 24 items	33.5%	*n* = 668	*Mean* = 12.89 years	Total ICU: *M* = 18.96, *SD* = 8.40;<br>Callous: *M* = 11.76, *SD* = 10.56;<br>Uncaring: *M* = 16.32, SD = 10.32;<br>Unemotional: *M* = 35.76,	---	---	---	---

								SD = 15.12				
Barroso et al. (2021) Portugal	Community	ICU - self-report	0-3	Total: 19 items; Callous: 11 items; Uncaring: 8 items	65.4%	*n* = 4281	12-20 years	Non-abusive sexting behaviors Callous: *M* = 11.56, *SD* = 5.28; Uncaring: *M* = 15.85, *SD* = 5.28; Abusive Sexting Behaviors Callous: *M* = 14.13, *SD* = 5.63; Uncaring: *M* = 14.16, *SD* = 5.23; Non-Abusive Sexting Victimizatio Callous: *M* = 11.64, *SD* = 5.29; Uncaring: *M* = 15.80, *SD* = 5.52; Abusive sexting victimization Callous: *M* = 12.72, *SD* = 5.89; Uncaring: *M* = 15.00, *SD* = 5.59	---	---	---	---
Bell et al. (2018) USA	Community (with diagnosis)	ICU - self-report	0-3	Total: 24 items	74.0%	DBD: *n* = 81	13-18 years old	Total ICU: *M* = 30.23, *SD* = 10.43	---	---	---	---
Benesch et al. (2014) Germany	Clinic	ICU - self-report	0-3	Original ICU Total: 24 items; Callous: 11 items; Uncaring: 8 items New ICU Total ICU: 21 items; Callousness/Lack of Guilt or Remorse: 11 items; Unconcerned about performance: 5 items;	100%	*n* = 135	6-12 years	Original ICU Total ICU: *M* = 28.80, *SD* = 7.92; Callous: *M* = 9.57, *SD* = 8.88; Uncaring: *M* = 13.12, *SD* = 3.76; New ICU Total ICU: *M* = 26.46, *SD* = 7.54; Callousness: *M* = 14.41, *SD* = 4.95; Unconcerned about performance: *M* = 5.90, *SD* = 2.80;	---	---	---	---

Bennett & Kering (2014) USA	Forensic	ICU - self-report	0-3	Total: 24 items	73.4%	*n* = 417	12-18 years old	ICU Total: *M* = 22.33, *SD* = 9.71	---	---	---	---
Berg et al. (2013) USA	Community	ICU-self-report and caregiver version	0-3	Total: 24 items	55.7%	*n* = 117	13-17 years old	Self-report Total ICU: *M* = 24.06, *SD* = 8.57 Caregiver report Total ICU: *M* = 30.92, *SD* = 10.16	---	---	---	---
Bhanwer et al. (2019) Canada	Forensic	ICU - self-report	0-3	Total: 22 items; Callous: 9 items; Unemotional: 5 items; Uncaring: 8 items	62.9%	*n* = 70	13-18 years old	Total ICU: *M* = 26.42, SD = 9.50; Callous: *M* = 5.79, *SD* = 4.08; Uncaring: *M* = 10.41, *SD* = 5.30; Unemotional: *M* = 7.92, *SD* = 2.83	---	---	---	---
Bird et al. (2019) UK	Community	ICU - self-report	0-3	Total: 24 items	50.6%	*n* = 437	7-9 years	Total ICU: *M* = 21.28, *SD* = 7.88	---	---	---	---
Bisby et al. (2017) Australia	Forensic	ICU - self-report	0-3	Total: 22 items Callous: 9 items; Unemotional: 5 items; Uncaring: 8 items	100%	*n* = 227	12-19 years old	Total ICU: *M* = 25.74, *SD* = 10.12; Uncaring: *M* = 11.20, *SD* = 5.28; Unemotional: *M* = 8.25, *SD* = 2.90; Callous: *M* = 6.30, *SD* = 4.59	---	---	---	---
Blair et al (2020) USA	Clinic (with and without DBD diagnosis)	ICU - self-report and parent version	0-3	Total: 24 items	CD: 62.7% Psychiatric: 59.7%	*CD: n* = 67 *psychiatric: n* = 77	14-18 years old	CD Total ICU: *M* = 26.11, *SD* = 8.39; Psychiatric Total ICU: *M* = 23.83, *SD* = 8.36	63.6%	*n* = 51	14-18 years old	Total ICU: *M* = 15.85, *SD* = 7.08
Blair et al. (2021) USA	Community	ICU - self-report	0-3	Total: 24 items	69.4%	*n* = 98	*Mean* = 15.96 years	Total ICU: *M* = 23.93, *SD* = 7.89	---	---	---	---
Blanchard et al. (2017) UK	Community	ICU - parent version	0-3	Total: 23 items	39.2%	*n* = 79	5-6 years	Total ICU: *M* = 16.34, *SD* = 4.64	---	---	---	---
Bonham et	Communit	ICU -	0-3	Total: 24	60.1%	*n* = 148	6-11	Total ICU: *M* = 22.59, *SD*	---	---	---	---

al. (2022) Australia	y	parent version		items			years	= 9.77				
Bours et al. (2018) USA	Forensic	ICU - self-report and parent version	0-3	Total: 24 items	100%	CD: *n* = 44	12-19 years old	Self-report<br>Total ICU: *M* = 26.80, *SD* = 8.80;<br>Parent report<br>Total ICU: *M* = 28.80, *SD* = 11.30	100%	*n* = 28	12-19 years old	Self-report<br>Total ICU: *M* = 23.60, *SD* = 6.30;<br>Parent report<br>Total ICU: *M* = 17.00, *SD* = 17.10
Böttinger et al. (2024) Germany	Clinic	ICU – self-report and parent version	0-3	Total: 24 ietns	78%	*n = 25*	12-18 years old	Self-report<br>Treatment group 1<br>Total ICU: *M* = 31.82, *SD* = 9.75;<br>Treatment group 2<br>Total ICU: *M* = 30.08, *SD* = 7.63;<br>Parent report<br>Treatment group 1<br>Total ICU: *M* = 37.67, *SD* = 10.42;<br>Treatment group 2<br>Total ICU: *M* = 38.25, *SD* = 8.11	---	---	---	---
Breaux et al. (2020) USA	Clinic	ICU – parent and teacher versions	0-3	Total: 24 items	80.2%	*n* = 222	5-12 years	Parent report<br>Total ICU: *M* = 26.1, *SD* = 11.6;<br>Teacher report<br>Total ICU: *M* = 32.3, *SD* = 13.8	---	---	---	---
Cao et al. (2024) China	Communit y	ICU – teacher version	0-3	Total: 24 items;	48%	*n* = 116	4-6 years old	Total ICU: *M* = 23.41, *SD* = 10.54;	---	---	---	---
Cardinale et al. (2017) USA	Communit y (with and without DBD diagnosis)	ICU - self-report	0-3	Total: 22 items;<br>Callous: 9 items;<br>Unemotional: 5 items;<br>Uncaring: 8 items	50.0%	DBD: *n* = 30	10-17 years old	Low CU Group<br>Total ICU: *M* = 37.81, *SD* = 5.63;<br>Unemotional: *M* = 9.07, *SD* = 2.49;<br>Callous: *M* = 11.55, *SD* = 3.15;<br>Uncaring: *M* = 15.80, *SD* = 3.59;<br>High CU Group<br>Total ICU: *M* = 52.73, *SD* = 4.74;<br>Unemotional: *M* = 11.40, *SD* = 2.20<br>Callous: *M* = 20.00, *SD* =	50.0 %	*n* = 18	10-17 years old	Total ICU: *M* = 25.39, *SD* = 6.17;<br>Unemotional: *M* = 8.11, *SD* = 2.03;<br>Callous: *M* = 5.67, *SD* = 2.50;<br>Uncaring: *M* = 10.22, *SD* = 4.58

								4.58 Uncaring: *M* = 19.80, *SD* = 2.91				
Cardinale et al. (2019) USA	Community	ICU - self-report	0-3	Total: 22 items; Callous: 9 items; Unemotional: 5 items; Uncaring: 8 items;	59.5 %	*n* = 148	9-18 years old	Total ICU: *M* = 36.64, *SD* = 12.11; Callous: *M* = 11.33, *SD* = 6.55; Uncaring: *M* = 14.83, *SD* = 5.25; Unemotional: *M* = 8.98, *SD* = 2.54	---	---	---	---
Carvalho et al (2018) Portugal	Community	ICU - self-report	1-5	Total: 24 items	48.9%	*n* = 1011	7-17 years old	Boys Total ICU: *M* = 28.26, *SD* = 7.6; Uncaring: *M* = 21.13, *SD* = 5.50; Callous: *M* = 8.39, *SD* = 5.30; Girls Total ICU: *M* = 27.31, *SD* = 8.6; Uncaring: *M* = 19.06, *SD* = 6.20; Callous: *M* = 7.30, *SD* = 5.50	---	---	---	---
Castagna et al. (2021a) USA	Community	ICU - self-report	0-3	Total: 24 items	51.5%	*n* = 1050	5-12 years	Total ICU: *M* = 22.80, *SD* = 12.00	---	---	---	---
Castagna et al. (2021b) USA	Community	ICU - self-report	0-3	Total: 24 items	67.9%	*n* = 265	4-14 years	Total ICU: *M* = 25.68, *SD* = 11.04	---	---	---	---
Castagna et al. (2025) USA	Community	ICU – parent version	0-3	Total: 24 items	58.5%	*n* = 200	8-15 years old	Total ICU: *M* = 24.30, *SD* = 11.69	---	---	---	---
Cecil et al. (2018) USA	Community	ICU - teacher version	0-3	Total: 24 items; Callous: 11 items; Unemotional: 5 items; Uncaring: 8 items	46.0%	*n* = 204	*Mean* = 18.48, *SD* = 2.11	Total ICU: *M* = 23.21, *SD* = 9.53; Callous: *M* = 4.76, *SD* = 3.71; Uncaring: *M* = 11.78, *SD* = 5.35; Unemotional: *M* = 6.72, *SD* = 2.67	---	---	---	---
Chang et al. (2021) China	Forensic	ICU - self-report	0-3	Total: 22 items	100%	*n* = 441	*Mean* = 18.40, *SD* =	ICU Total: *M* = 16.95, *SD* = 4.94	100%	*n* = 543	*Mean* = 18.40, *SD* = 1.40 years old	Total ICU: *M* = 15.64, *SD* = 3.84

							1.40 years old					
Charles et al. (2012) USA	Community	ICU - parent version	0-3	Total: 24 items; Callous: 11 items; Unemotional: 5 items; Uncaring: 8 items	49.6%	*n* = 234	10-12 years	Girls<br>Total ICU: *M* = 18.08, *SD* = 8.94;<br>Callous: *M* = 4.86, *SD* = 3.90;<br>Unemotional: *M* = 4.34, *SD* = 2.97;<br>Uncaring: *M* = 8.88, *SD* = 4.32;<br>Boys<br>Total ICU: *M* = 21.29, *SD* = 9.45;<br>Callous: *M* = 5.29, *SD* = 3.68;<br>Unemotional: *M* = 5.13, *SD* = 2.96<br>Uncaring: *M* = 10.87, *SD* = 5.11	---	---	---	---
Chow et al. (2017) UK	Community	ICU - self-report	0-3	Total: 24 items	48.4%	*n* = 95	14-15 years old	Total ICU: *M* = 20.73, *SD* = 1.05	---	---	---	---
Ciucci & Baroncelli (2014) Italy	Community	ICU - self-report	0-3	Total: 22 items; Callous: 9 items; Unemotional: 5 items; Uncaring: 8 items	46.7%	*n* = 529	10-15 years	Callous: *M* = 4.77, *SD* = 3.60;<br>Uncaring: *M* = 6.00, *SD* = 3.76;<br>Unemotional: *M* = 7.00, *SD* = 3.10	---	---	---	---
Ciucci et al. (2014) Italy	Community	ICU - self-report	0-3	Total: 24 items; Callous: 11 items; Unemotional: 5 items; Uncaring: 8 items	47.4%	*n* = 540	11-14 years old	Total ICU: *M* = 0.82, *SD* = 0.37;<br>Callous: *M* = 0.54, *SD* = 0.40;<br>Uncaring: *M* = 0.76, *SD* = 0.47;<br>Unemotional: *M* = 1.41, *SD* = 0.62	---	---	---	---
Ciucci et al. (2018) Italy	Community	ICU - self-report	0-3	Total: 24 items	47.0%	*n* = 251	11-15 years	Total ICU: *M* = 19.44, *SD* = 8.64	---	---	---	---
Clark & Frick (2018)	Community	ICU - self-report	0-3	Total: 21 items	39.0%	*n* = 92	*Mean* = 4.6-7.2	Total ICU: *M* = 19.66, *SD* = 8.27	---	---	---	---

USA							years					
Coe et al. (2018) USA	Community	ICU - self-report	0-3	Callousness: 11 items	44.0%	*n* = 243	*Mean* = 5.75 years	Callousness: *M* = 4.18, *SD* = 3.19	---	---	---	---
Colins et al. (2016) Belgium	Forensic	ICU - self-report	0-3	Total: 24 items;<br>Callous: 11 items;<br>Unemotional: 5 items;<br>Uncaring: 8 items	0%	*n* = 191	12-17 years old	Total ICU: *M* = 27.73, *SD* = 10.25;<br>Callous: *M* = 9.42, *SD* = 6.09;<br>Uncaring: *M* = 10.23, SD = 5.22;<br>Unemotional: *M* = 8.07, *SD* = 3.18	---	---	---	---
Colins et al. (2018) Cyprus	Community (with and without DBD)	ICU - parent version	0-3	Total: 24 items	46.5%	*n* = 690	7-12 years	CP boys<br>Total ICU: *M* = 20.83, *SD* = 6.19;<br>CU boys<br>Total ICU: *M* = 33.09, *SD* = 3.97;<br>PP boys<br>Total ICU: *M* = 36.36, *SD* = 5.40;<br>CP+CU boys<br>Total ICU: *M* = 35.40, *SD* = 4.69;<br>CP+PP boys<br>Total ICU: *M* = 36.17, *SD* = 7.63;<br>CD girls<br>Total ICU: *M* = 15.00, *SD* = 6.26;<br>CU girls<br>Total ICU: *M* = 31.38, *SD* = 4.84<br>PP girls<br>Total ICU: *M* = 34.78, *SD* = 5.22;<br>CP+CU girls<br>Total ICU: *M* = 33.08, *SD* = 4.48;<br>CP+PP girls<br>Total ICU: *M* = 43.71, *SD* = 5.97	46.5%	*n* = 690	7-12 years	Boys<br>Total ICU: *M* =14.99, *SD* = 7.12<br>Girls<br>Total ICU: *M* = 14.03, *SD* = 6.41
Corbelli et al. (2024) Italy	Community	ICU – self-report	0-3	Total: 11 items;<br>Callous: 6	49.8%	Boys<br>*n* = 368<br>Girls	NA	Boys<br>Callous: *M* = 2.92, *SD* = 2.91;	---	---	---	---

				items; Uncaring: 5 items		*n* = 371		Uncaring: *M* = 4.85, *SD* = 3.28; Girls Callous: *M* = 2.32, *SD* = 2.43; Uncaring: *M* = 3.96, *SD* = 2.93;				
Craig et al. (2019) Netherlands	Community (with and without DBD)	ICU - parent version	0-3	Total: 22 items; Callous: 9 items; Unemotional: 5 items; Uncaring: 8 items	83.6%	DBD: *n* = 140	8-18 years old	Total ICU: *M* = 21.2, *SD* = 8.1; Callous: *M* = 4.00, *SD* = 2.80; Unemotional: *M* = 6.50, *SD* = 2.90 Uncaring: *M* = 7.80, *SD* = 3.50	59.14%	*n* = 93	8-18 years old	Total ICU: *M* = 32.40, *SD* = 9.80 Callous: *M* = 7.50, *SD* = 4.60; Unemotional: *M* = 7.60, *SD* = 2.50 Uncaring: *M* = 11.00, *SD* = 4.40
Craig & Moretti (2019) UK	Clinic	ICU - self-report	0-3	Total: 22 items	43.3%	*n* = 418	12-19 years old	Total ICU: *M* = 10.21, *SD* = 5.82	---	---	---	---
Dandreaux & Frick (2009) USA	Forensic	ICU-self-report version	0-3	Total: 24 items	100%	*n* = 98	11-18 years old	Total ICU: *M* = 25.99, *SD* = 9.52	---	---	---	---
Dapprich et al. (2022) Netherlands	Community	ICU - self-report and parent version	0-3	Total: 24 items	51.9%	*n* = 129	14 and 17 years old	Self-report 17 years – Total ICU: *M* = 21.21, *SD* = 6.60; Parent report 14 years Total ICU: *M* = 20.70, *SD* = 9.10 17 years Total ICU: *M* = 21.90, *SD* = 8.40;	---	---	---	---
Dapprich et al. (2024) Netherlands	Community	ICU – self-report	0-3	Total: 24 items;	36%	Boys: *n* = 390 Girls: *n* = 247	*Mean* = 14.6 years old	Boys Total ICU: *M* = 28.00, *SD* = 9.00; Girls Total ICU: *M* = 24.00, *SD* = 8.00;	---	---	---	---
Dargis & Li (2020) USA	Community	ICU - parent version	0-3	Total: 24 items; Callous: 11 items;	100%	*n* = 201	5-6 years	Total ICU: *M* = 16.98, *SD* = 6.96; ICU – Callous: *M* = 3.66,	---	---	---	---

				Unemotional: 5 items; Uncaring: 8 items				*SD* = 2.78; Unemotional: *M* =2.95, *SD* = 2.09; Uncaring: *M* =10.40, *SD* = 4.06				
Davis et al. (2015) USA	Community	ICU - self-report	0-3	Total: 24 items	57.0%	*n* = 236	11-17 years old	Total ICU: *M* = 0.70, *SD* = 0.28	---	---	---	---
De Brito et al. (2025) UK	Community (with and without DBD)	ICU – parent version	0-3	Total: 24 items; Callous: 11 items; Unemotional: 5 items; Uncaring: 8 items	54%	CD: *n* = 161	9-18 years old	Boys Total ICU: *M* = 34.70, *SD* = 12.20; Callous: *M* = 12.40, *SD* = 6.30; Uncaring: *M* = 7.50, *SD* = 3.20; Unemotional: *M* = 14.80, *SD* = 5.20; Girls Total ICU: *M* = 33.10, *SD* = 11.50; Callous: *M* = 11.80, *SD* = 6.20; Uncaring: *M* = 7.40, *SD* = 3.80; Unemotional: *M* = 13.90, *SD* = 4.50;	57.7%	*n* = 241	9-18 years old	Boys Total ICU: *M* = 18.60, *SD* = 6.10; Callous: *M* = 4.30, *SD* = 2.10; Uncaring: *M* = 5.40, *SD* = 2.70; Unemotional: *M* = 8.90, *SD* = 3.50; Girls Total ICU: *M* = 15.40, *SD* = 6.40; Callous: *M* = 3.80, *SD* = 2.60; Uncaring: *M* = 4.20, *SD* = 2.50; Unemotional: *M* = 7.30, *SD* = 3.90;
DeLisi et al. (2011) USA	Community (with and without ADHD)	ICU - teacher version	0-3	Total: 24 items; Callous: 11 items; Unemotional: 5 items; Uncaring: 8 items	60.8%	ADHD: *n* = 382	12-14 years old	Attentive/non-hyperactive Total ICU: *M* = 51.44, *SD* = 6.61; Callous: *M* = 24.86, *SD* = 3.79; Uncaring: *M* = 12.82, *SD* = 2.90; Unemotional: *M* = 13.75, *SD* = 2.40; Attentive/hyperactive Total ICU: *M* = 56.86, *SD* = 7.51; Callous: *M* = 29.14, *SD* = 4.82; Uncaring: *M* = 15.68, *SD* = 3.16; Unemotional: *M* = 12.51, *SD* = 3.83;	72.0%	*n* = 50	12-14 years old	Total ICU: *M* = 59.64, *SD* = 9.25; Callous: *M* = 27.95, *SD* = 4.96; Uncaring: *M* = 16.83, *SD* = 3.35; Unemotional: *M* = 14.86, *SD* = 3.57;

								Inattentive/hyperactive Total ICU: *M* = 62.98, *SD* = 10.97; Callous: *M* = 32.54, *SD* = 6.87; Uncaring: *M* = 17.93, *SD* = 3.56; Unemotional: *M* = 11.61, *SD* = 3.04				
Derks et al. (2024a) Germany	Clinical	ICU – self-report	0-3	Total: 24 items	67.3%	CD: *n* = 978	12-18 years old	Total ICU: *M* = 26.67, *SD* = 9.43;	---	---	---	---
Derks et al. (2024b) Germany	Clinical and Community	ICU – parent version	0-3	Total: 24 items	100%	CD: *n* = 40	10-14 years old	Total ICU: *M* = 35.36, *SD* = 10.13;	100%	*n* = 30	10-14 years old	Total ICU: *M* = 17.70, *SD* = 6.38;
Docherty et al. (2016) USA	Forensic	ICU - self-report version, parent and teachers version	0-3	Total: 22 items	47.5 %	*n* = 779	*Mean* = 15.57-16.84 years old	Self-report Total ICU: *M* = 22.22, *SD* = 8.58; Parent report Total ICU: *M* = 26.18, *SD* = 12.98 Teacher report Total ICU: *M* = 27.72, *SD* = 10.56	---	---	---	---
Dotterer et al. (2021) USA	Community	ICU - self-report	0-3	Total: 22 items	55.9%	*n* = 474	6-10 years old	ICU Total: *M* =17.72, *SD* = 6.68	---	---	---	---
Drent et al. (2020) Netherlands	Clinical	ICU - parent version	0-3	Total: 24 items	60.3%	*n* = 521	18 years old	Total ICU: *M* = 54.58, *SD* = 10.32	---	---	---	---
Dugré & Potvin (2023) USA	Community	ICU - parent version	0-3	Total: 24 items; Callous: 11 items; Unemotional: 5 items; Uncaring: 8 items	Anx: 58% CU/Anx: 53%	Anx: *n* = 231; CU/Anx: *n* = 45	5-21 years old	Anx Total ICU: *M* = 25.20, *SD* = 11.01; Callous: *M* = 5.75, *SD* = 4.35; Uncaring: *M* = 11.76, *SD* = 5.23; Unemotional: *M* = 5.81, *SD* = 3.37; CU/Anx Total ICU: *M* = 31.15, *SD* = 10.70; Callous: *M* = 8.23, *SD* = 5.26;	63.3%	*n* = 1039	5-21 years old	Total ICU: *M* = 23.66, *SD* = 10.39; Callous: *M* = 4.93, *SD* = 3.97; Uncaring: *M* = 11.81, *SD* = 4.92; Unemotional: *M* = 4.84, *SD* = 3.05;

								Uncaring: *M* = 13.85, *SD* = 5.42;<br>Unemotional: *M* = 7.54, *SD* = 3.60;				
Eisenbarth et al. (2016) Cyprus	Community	ICU - self-report	0-3	Total: 24 items	52.9%	*n* = 2023	15-18 years old	Low - time 1<br>Total ICU: *M* = 19.99, *SD* = 6.65;<br>CU- time 1<br>Total ICU: *M* = 34.15, *SD* = 4.13;<br>Increasing - time 1<br>Total ICU: *M* = 25.25, *SD* = 7.97;<br>CD+CU - time 1<br>Total ICU: *M* = 35.86, *SD* = 6.49;<br>CD - time 1<br>Total ICU: *M* = 23.61, *SD* = 5.32	---	---	---	---
Elizur et al. (2017) Israel	Community	ICU – parent report	0-3	Total: 24 items	77.9%	*n* = 209	3-6 years	Pretreatment<br>Total ICU: *M* = 29.43, *SD* = 5.76;<br>Post treatment<br>Total ICU: *M* = 27.88, *SD* = 5.41	77.9%	*n* = 209	3-6 years	Pretreatment<br>Total ICU: *M* = 28.84, *SD* = 4.49;<br>Post treatment<br>Total ICU: *M* = 30.59, *SD* = 4.71
Elowsky et al. (2022) USA	Clinical and Community	ICU - self-report	0-3	Total: 24 items	58.0%	CD: *n* = 193	10-18 years	Total ICU: *M* = 25.46, *SD* = 8.03	58.0%	*n* = 193	10-18 years	Total ICU: *M* = 15.51, *SD* = 6.61
Essau et al. (2006) Germany	Community	ICU - self-report	0-3	Total: 24 items;<br>Callous: 11 items;<br>Unemotional: 5 items;<br>Uncaring: 8 items	53.6%	*n* = 1443	13-18 years old	Boys<br>Total ICU: *M* = 27.12, *SD* = 7.70;<br>Callous: *M* = 8.57, *SD* = 4.60;<br>Uncaring: *M* = 8.93, *SD* = 4.20;<br>Unemotional: *M* = 7.65, *SD* = 2.60;<br>Girls<br>ICU Total: *M* = 21.64, *SD* = 6.00;	---	---	---	---

								Callous: *M* = 5.80, *SD* = 3.60;<br>Uncaring: *M* = 7.22, *SD* = 3.5;<br>Unemotional: *M* = 5.92, *SD* = 2.70				
Euler et al. (2014) Sweden	Clinical and Community	ICU - self-report	0-3	Total: 24 items;<br>Callous: 11 items;<br>Unemotional: 5 items;<br>Uncaring: 8 items	100%	CD: *n* = 22	11-17 years old	Total ICU: *M* = 25.20, *SD* = 9.07;<br>Callous: *M* = 8.85, *SD* = 5.10;<br>Uncaring: *M* = 9.85, *SD* = 4.13;<br>Unemotional: *M* = 6.50, *SD* = 2.69	100%	*n* = 22	11-17 years old	Total ICU: *M* = 22.20, *SD* = 5.71<br>Callous: *M* = 6.20, *SD* = 2.40;<br>Uncaring: *M* = 8.90, *SD* = 4.18;<br>Unemotional: *M* = 7.10, *SD* = 1.77
Everett & Drabick (2023) USA	Community	ICU - parent and teacher version	0-4	Total: 24 items	50%	*n* = 104	*Mean* = 9.93 years	Parent report<br>Total ICU: *M* = 23.60, *SD* = 10.48;<br>Teacher report<br>Total ICU: *M* = 32.09, *SD* = 10.88	---	---	---	---
Ezpeleta et al. (2012) Spain	Community	ICU-Teacher version	0-3	Total: 24 items;<br>Callous: 11 items;<br>Unemotional: 5 items;<br>Uncaring: 8 items	50.9%	*n* = 620	3-4 years	Boys 3 years old<br>Total ICU: *M* = 51.2, *SD* = 4.04;<br>Callous: *M* = 50.5, *SD* = 4.56;<br>Uncaring: *M* = 50.5, *SD* = 2.96;<br>Unemotional: *M* = 50.7, *SD* = 3.60;<br>Girls 3 years old<br>Total ICU: *M* = 48.3, *SD* = 1.67;<br>Callous: *M* = 47.3, *SD* = 2.28;<br>Uncaring: *M* = 48.4, *SD* = 0.75;<br>Unemotional: *M* = 48.0, *SD* = 1.83	---	---	---	---
Ezpeleta et al. (2015) Spain	Community	ICU - teacher version	0-3	Total: 24 items;<br>Callous: 11 items;<br>Unemotional: 5 items;<br>Uncaring: 8 items	50.9%	3 years: *n* = 622<br><br>5 years: *n* = 574	3-5 years	3 years<br>Total ICU: *M* = 21.19, *SD* = 10.02;<br>Callous: *M* = 4.92, *SD* = 4.23;<br>Uncaring: *M* = 11.44, *SD* = 5.22;<br>Unemotional: *M* = 4.76,	---	---	---	---

								SD = 3.30;<br>5 years<br>Total ICU: *M* = 19.75, *SD* = 9.60;<br>Callous: *M* = 3.92, *SD* = 3.47;<br>Uncaring: *M* = 10.59, *SD* = 5.18<br>Unemotional: *M* = 5.22, *SD* = 3.38				
Ezpeleta et al. (2017)<br>Spain	Communit y	ICU - teacher version	0-3	Total: 24 items	50.0%	*n* = 320	3-8 years	Total ICU: *M* = 21.1, *SD* = 12.1	---	---	---	---
Ezpeleta et al. (2019)<br>Spain	Communit y	ICU – teacher version	0-3	Total: 24 items	53.3%	*n* = 377	3-9 years	3 years<br>Total ICU: *M* = 20.45, *SD* = 9.28;<br>4 years<br>Total ICU: *M* = 20.45, *SD* = 9.67;<br>5 years<br>Total ICU: *M* = 19.81, *SD* = 9.38;<br>6 years<br>Total ICU: *M* = 20.72, *SD* = 9.91;<br>7 years<br>Total ICU: *M* = 20.49, *SD* = 9.71;<br>8 years<br>Total ICU: *M* = 20.23, *SD* = 11.23;<br>9 years<br>Total ICU: *M* = 20.38, *SD* = 10.35	---	---	---	---
Facci et al. (2024)<br>Italy	Communit y	ICU - parent version	0-3	Total: 24 Items	NA	*n* = 163	3-5 years old	Total ICU: *M* = 15.60, *SD* = 7.92;	---	---	---	---
Fagan et al. (2017)<br>USA	Communit y	ICU - parent version	0-3	Total: 19 items;<br>Callous: 7 items;<br>Unemotional: 4 items;<br>Uncaring: 8	48.2%	*n* = 445	7-10 years	Boys<br>Total ICU: *M* = 15.75, *SD* = 7.65;<br>Callous: *M* = 2.04, *SD* = 2.36;<br>Uncaring: *M* = 9.69, *SD* = 4.90;	---	---	---	---

				items				Unemotional: *M* = 4.02, *SD* = 2.25; Girls Total ICU: *M* = 14.61, *SD* = 7.95; Callous: *M* = 1.84, *SD* = 2.56; Uncaring: *M* = 9.09, *SD* = 5.05; Unemotional: *M* = 3.68, *SD* = 2.41				
Fairchild et al. (2014) USA	Clinical and Community	ICU - parent version	0-3	Total: 24 items	0%	CD: *n* = 20	*Mean* = 16.97 years	Total ICU: *M* = 28.78, *SD* = 4.11	0%	*n* = 20	*Mean* = 17.6 years	Total ICU: *M* = 17.81, *SD* = 8.04
Fang et al. (2020) China	Community	ICU - self-report	0-3	Total: 24 items	30.3%	*n* = 839	17-24 years old	Total ICU: *M* = 48.00, *SD* = 6.00	---	---	---	---
Fang et al. (2022) China	Community	ICU - self-report	0-3	Total: 24 items	49.5%	*n* = 2407	12-15 years	Total ICU: *M* = 48.68, *SD* = 8.64	---	---	---	---
Fanniff & Kimonis (2014) USA	Forensic	ICU - self-report	0-3	Total: 24 items; Callous: 11 items; Unemotional: 5 items; Uncaring: 8 items;	100%	*n* = 227	12-19 years old	Juvenile violent delinquentes ICU – Uncaring: *M* = 1.17, *SD* = 0.60; Unemotional: *M* = 1.52, *SD* = 0.55; Callous: *M* =0.58, *SD* = 0.45; Offenders Uncaring: *M* = 1.60, *SD* = 0.65; Unemotional: *M* = 1.77, *SD* = 0.58; Callous: *M* = 0.81, *SD* = 0.53	---	---	---	---
Fanti & Kimonis (2012) USA	Community	ICU - self-report	0-3	Total: 24 items	49.9%	*n* = 1416	12-14 years old	Boys ICU Total: *M* = 22.87, *SD* = 8.57; Girls ICU Total: *M* =19.13, *SD* = 7.96	---	---	---	---
Fanti &	Community	ICU - self-	0-3	Total: 24	49.9%	*n* = 1674	12-14	Low CP and CU	---	---	---	---

Kimonis (2013) Greece	y	report		items;			years old	Total ICU: *M* = 16.64, *SD* = 0.22; Moderate CP and CU Total ICU: *M* = 24.85, *SD* = 0.26; Low CP and high CU Total ICU: *M* = 35.18, *SD* = 0.58; High CP and low CU Total ICU: *M* = 22.02, *SD* = 0.67; High CP and CU ICU Total: *M* = 29.54, *SD* = 0.80				
Fanti et al. (2009) Greece	Community	ICU - self-report	0-3	Total: 24 items	50.7%	*n* = 347	12-18 years old	Boys Total ICU: *M* = 21.63, *SD* = 8.86; Callous: *M* = 6.44, *SD* = 4.09; Uncaring: *M* = 7.98, *SD* = 4.86; Unemotional: *M* = 7.22, *SD* = 3.06; Girls Total ICU: *M* = 16.52, *SD* = 8.14; Callous: *M* = 4.57, *SD* = 3.91; Uncaring: *M* = 5.51, *SD* = 4.34; Unemotional: *M* = 6.44, *SD* = 3.32	---	---	---	---
Fanti et al. (2013a) USA	Forensic	ICU - self-report	0-3	Total: 24 items; Callous: 11 items; Unemotional: 5 items; Uncaring: 8 items;	100%	*n* = 227	12-19 years old	Uncaring: *M* = 11.12, *SD* = 5.28; Unemotional: *M* = 8.25, *SD* = 2.85; Callous: *M* = 7.70, *SD* = 5.50	---	---	---	---
Fanti et al. (2013b) Cyprus	Community	ICU - self-report	0-3	Total: 24 items; Callous: 11 items; Unemotional: 5 items;	50.3%	*n* = 2306	15-18 years old	Total ICU: *M* = 23.65, *SD* = 8.72	---	---	---	---

				Uncaring: 8 items								
Fanti & Kimonis (2013) Greece	Community	ICU - self-report	0-4	Total: 24 items	49.9%	*n* = 1416	11-14 years old	Total ICU: *M* = 20.34, *SD* = 8.74	---	---	---	---
Fanti et al. (2017) USA	Community	ICU - self-report	0-3	Total: 24 items	46.6%	*n* = 1311	*Mean* = 9.38 years	Low CU<br>Total ICU: *M* =14.51, *SD* = 0.33;<br>Aumented CU<br>Total ICU: *M* = 26.42, *SD* = 0.63;<br>CU Decreased<br>Total ICU: *M* = 16.22, *SD* = 0.51;<br>CU High Stable<br>Total ICU: *M* = 28.52, *SD* = 0.55	---	---	---	---
Fanti et al. (2018) Cyprus	Community	ICU-self-report and parent version	0-3	Total: 24 items	Childhood: 48.0%<br><br>Adolescence: 51.0%	Childhood: *n* = 1599<br><br>Adolescence: *n* = 2719	*Mean* = 9.46 years	Total ICU: *M* = 23.33, *SD* = 8.94	---	---	---	---
Fanti et al (2019) Greece	Community	ICU - self-report	0-4	Total: 24 items	48.5%	*n* = 1887	10-12 years	ICU Total: *M* = 7.16, *SD* = 4.61	---	---	---	---
Feilhauer et al. (2012) Netherlands	Forensic	ICU - self-report	0-3	Total: 24 items;<br>Callous: 11 items;<br>Unemotional: 5 items;<br>Uncaring: 8 items	100%	*n* = 383	8-20 years	Clinical Offenders<br>Total ICU: *M* = 27.80, *SD* = 8.85;<br>Uncaring: *M* = 2.24, *SD* = 2.15;<br>Unemotional: *M* = 4.99, *SD* = 2.79;<br>Callous: *M* =5.18, *SD* = 2.59;<br>Clinical Non-Offenders<br>Total ICU: *M* = 29.45, *SD* = 8.99;<br>Uncaring: *M* = 3.23, *SD* = 2.76;<br>Unemotional: *M* = 5.45, *SD* = 2.70;<br>Callous: *M* = 4.98, *SD* = 2.97	100%	*n* = 383	8-20 years	Total ICU: *M* = 23.45, *SD* = 8.06<br>Uncaring: *M* = 2.43, *SD* = 2.78<br>Unemotional: *M* = 3.94, *SD* = 2.28<br>Callous: *M* = 4.50, *SD* = 2.52

								Externalizing Non-Offenders<br>Total ICU: M = 28.52, SD = 6.38;<br>Uncaring: *M* = 5.88, *SD* = 2.83;<br>Unemotional: *M* = 4.17, *SD* = 2.08;<br>Callous: *M* = 3.88, *SD* = 2.74				
Feilhauer et al. (2013a) Netherlands	Community	ICU - self-report	0-3	Total: 24 items	100%	*n* = 46	8-12 years old	ICU Total: *M* = 20.30, *SD* = 7.23	---	---	---	---
Feilhauer et al. (2013b) Netherlands	Forensic and Community	ICU - self-report	0-3	Total: 24 items	100%	*n* = 63	15-17 years old	ICU Total: *M* = 28.10, *SD* = 9.38	100%	*n* = 62	15-17 years old	Total ICU: *M* = 22.31, *SD* = 7.70
Figueiredo et al. (2022) Portugal	Community	ICU - teacher version	0-3	Total: 11 items;<br>Callous: 6 items;<br>Uncaring: 5 items	49.4%	*n* = 178	6-10 years	Total ICU: *M* = 7.92, *SD* = 4.79;<br>Callous: *M* = 1.75, *SD* = 2.22;<br>Uncaring: *M* = 6.16, *SD* = 6.16	---	---	---	---
Figueiredo et al. (2023) Portugal	Community	ICU - teacher version	0-3	Total: 11 items;<br>Callous: 6 items;<br>Uncaring: 5 items	48%	*n* = 236	3-10 years old	Total ICU: *M* = 8.76, *SD* = 5.37;<br>Callous: *M* = 2.10, *SD* = 2.89;<br>Uncaring: *M* = 6.65, *SD* = 3.31	---	---	---	---
Fine et al. (2017) USA	Forensic	ICU - self-report	0-3	Total: 24 items	100%	*n* = 325	15.49 years old	Total ICU: *M* = 26.90, *SD* = 7.75	---	---	---	---
Fink et al. (2012) USA	Forensic	ICU - self-report	0-3	Total: 22 items	77.5%	*n* = 190	17 years old	Total ICU: *M* = 26.68, *SD* = 8.40	---	---	---	---
Fisher & Brown (2018) USA	Community	ICU - parent version	0-3	Total: 24 items	49.5%	*n* = 390	8.06 years	Total ICU: *M* = 22.56, *SD* = 7.62	---	---	---	---
Fite et al. (2018) USA	Forensic	ICU - self-report	0-3	Total: 24 items	72.9%	*n* = 129	11-18 years old	Total ICU: *M* = 27.84, *SD* = 9.36	---	---	---	---
Fite et al. (2021) USA	Forensic	ICU - self-report	0-3	Total: 24 items	75.0%	*n* = 269	11-17 years old	Total ICU: *M* = 25.68, *SD* = 9.60	---	---	---	---

Fite et al. (2020) USA	Forensic	ICU - self-report	0-3	Total: 24 items; Callous: 11 items; Unemotional: 5 items; Uncaring: 8 items	70.3%	*n* = 111	11-17 years old	Total ICU: *M* = 25.92, *SD* = 7.92; Callous: *M* = 6.82, *SD* = 3.63; Uncaring: *M* = 9.92, *SD* = 4.16; Unemotional: *M* = 9.10, *SD* = 3.35	---	---	---	---
Fleming et al. (2022) USA	Communit y	ICU - parent version	0-3	Total: 24 items	84.0%	DBD: *n* = 43	3-7 years	Total ICU: *M* = 36.14, *SD* = 9.82	---	---	---	---
Fragkaki et al. (2016) Netherlands	Communit y	ICU - self-report	0-3	Total: 24 items	35.7%	*n* = 277	12-18 years old	Total ICU: *M* = 22.90, *SD* = 7.29	---	---	---	---
Fragkaki et al. (2019a) Netherlands	Forensic	ICU - self-report	0-3	Total: 24 items	100	*n* = 57	*Mean* = 17.95 years old	Total ICU: *M* = 29.95, *SD* = 9.98	---	---	---	---
Fragkaki et al. (2019b) Netherlands	Forensic	ICU - self-report	0-3	Total: 24 items	100%	*n* = 100	16.51 years old	Total ICU: *M* = 29.44, *SD* = 10.05	---	---	---	---
Fragkaki & Cima (2019) Netherlands	Forensic	ICU - parent version	0-3	Total: 24 items	100	*n* = 91	15-18 years old	Total ICU: *M* = 29.33, *SD* = 10.28	---	---	---	---
Gao & Raine (2024) USA	Communit y	ICU – parent version	0-3	Total: 24 items	56.5%	*n* = 92	13-16 years	Total ICU: *M* = 30.25, *SD* = 9.44	---	---	---	---
Gao & Zhang (2016) USA	Communit y	ICU - self-report and parent version	0-3	Self-report: Total: 13 items; Callous: 7 items; Uncaring: 6 items; Parent version: Total: 24 items; Callous: 11 items; Unemotional:	48.2%	*n* = 164	8-10 years	Parent report Total ICU: *M* = 15.10, *SD* = 7.72; Callous: *M* = 1.93, *SD* = 2.44; Uncaring: *M* = 9.34, *SD* = 4.92; Unemotional: *M* = 3.83, *SD* = 2.31; Self-report Total ICU: *M* = 5.42, *SD* = 4.78; Callous: *M* = 2.52, *SD* = 3.21;	---	---	---	---

				5 items; Uncaring: 8 items				Uncaring: *M* = 2.89, *SD* = 3.16				
Gao & Zhang (2021) USA	Community	ICU-self-report version	0-3	Total: 24 items	48.2%	*n* = 340	*Mean* = 9.06 years	Total ICU: *M* = 15.14, *SD* = 7.75	---	---	---	---
Gao et al. (2017) USA	Community	ICU - self-report and parent version	0-3	Total: 24 items	48.1%	*n* = 295	8-10 years	Self-report Total ICU: *M* = 18.89, *SD* = 7.03; Parent report Total ICU: *M* = 17.71, *SD* = 8.81	---	---	---	---
Gaule et al. (2024a) UK	Community	ICU – self-report	0-3	Total: 24 items	100%	CP/HCU: *n* = 46 CP/LCU: *n* = 46	11-16 years old	CP/HCU Total ICU: *M* = 47.15, *SD* = 6.69; CP/LCU Total ICU: *M* = 30.28, *SD* = 6.14;	100	*n* = 59	11-16 years old	Total ICU: *M* = 19.47, *SD* = 6.95;
Gaule et al. (2024b) UK	Community	ICU – teacher version	0-3	Total: 24 items	100%	CP/HCU: *n* = 30 CP/LCU: *n* = 36	11-16 years old	CP/HCU Total ICU: *M* = 45.24, *SD* = 5.66; CP/LCU Total ICU: *M* = 31.00, *SD* = 4.98;	100%	*n* = 33	11-16 years old	Total ICU: *M* = 19.48, *SD* = 6.06;
Georgiou et al. (2019) Cyprus	Community	ICU - parent version	0-3	Total: 24 items	55.8%	*n* = 163	3-8 years	Total ICU: *M* = 21.81, *SD* = 9.89	---	---	---	---
Gillen et al. (2019) USA	Forensic	ICU - self-report	0-3	Total: 24 items	80.5%	*n* = 160	16-19 years old	Total ICU: *M* = 26.92, *SD* = 7.52	---	---	---	---
Gluckman et al (2016) USA	Community	ICU - self-report version	0-3	Total: 24 items	57.0%	*n* = 158	9-12 years old	Total ICU: *M* = 19.46, *SD* = 7.41; Callous: *M* = 7.46, *SD* = 4.34; Uncaring: *M* = 5.08, *SD* = 2.96; Unemotional: *M* = 6.93, *SD* = 2.78	---	---	---	---
Goagoses & koglin (2021) Germany	Community	ICU - self-report	0-3	Total: 24 items	44.3%	*n* = 296	13-18 years old	Total ICU: *M* = 34.08, *SD* = 7.68	---	---	---	---
Goagoses et al. (2022) Germany	Community	ICU - self-report	0-3	Total: 24 items; Callous: 11	44.1%	*n* = 435	*Mean* =14.12 years	Callous: *M* = 6.71, *SD* = 4.62; Uncaring: *M* = 7.28, *SD* =	---	---	---	---

				items; Unemotional: 5 items; Uncaring: 8 items				4.16; Unemotional: *M* = 7.55, *SD* = 3.10				
Goffin et al (2017) USA	Community	ICU - parent version	0-4	Total: 24 items	50%	*n* = 87	8-12 years old	Total ICU: *M* = 17.28, *SD* = 6.24	---	---	---	---
Goffin et al. (2018) USA	Community	ICU - parent version	0-3	Total: 24 items	100%	*n* = 729	2-12 years	Total ICU: *M* = 17.28, *SD* = 6.24	---	---	---	---
Gong et al. (2022) China	Community	ICU - self-report	0-3	Total: 11 items	51.7%	*n* = 484	7-13 years old	Total ICU: *M* = 19.91, *SD* = 5.74; Uncaring: *M* = 8.06, *SD* = 2.28; Callous: *M* = 11.85, *SD* = 3.32	---	---	---	---
González-Madruga et al (2020) USA	Clinical and community	ICU - parent version	0-4	Total: 24 items	48.5%	CD: *n* = 101	13-18 years old	Girls Total ICU: *M* = 33.87, *SD* = 12.61; Callous: *M* = 10.56, *SD* = 5.31; Uncaring: *M* = 14.52, *SD* = 4.98; Unemotional: *M* = 7.54, *SD* = 4.09; Boys Total ICU: *M* = 34.10, *SD* = 10.92; Callous: *M* = 10.43, *SD* = 5.78; Uncaring: *M* = 15.27, *SD* = 4.52; Unemotional: *M* = 7.43, *SD* = 2.76	49.5%	*n* = 99	13-18 years old	Girls Total ICU: *M* = 14.66, *SD* = 5.32; Callous: *M* = 5.06, *SD* = 3.06; Uncaring: *M* = 6.60, *SD* = 3.32; Unemotional: *M* = 4.36, *SD* = 2.45 Boys Total ICU: *M* = 19.80, *SD* = 8.40 Callous: *M* = 5.82, *SD* = 3.06 Uncaring: *M* = 9.29, *SD* = 5.50 Unemotional: *M* = 6.10, *SD* = 3.06
Grassetti et al. (2023) USA	Clinical	ICU - self-report	0-3	Total: 24 items	82.9%	*n* = 292	16-19 years old	Total ICU: *M* = 26.04, *SD* = 9.58	---	---	---	---
Graziano et al. (2022) USA	Community	ICU - parent version	0-3	Total: 12 items	69.0%	DBD: *n* = 102	*Mean* = 5.66 years	Total ICU: *M* = 15.96, *SD* = 0.62	69.0%	*n* = 96	*Mean* = 5.66 years	Total ICU: *M* = 8.04, *SD* = 0.34
Grotzinger et al. (2018)	Community	ICU - self-report	0-3	Total: 22 items	52.0%	*n* = 891	*Mean* = 13.5-	ICU Total: *M* = 41.58, *SD* = 7.04	---	---	---	---

USA							20.1 years					
Guelker et al. (2014) USA	Forensic	ICU - self-report	0-3	Total: 22 items; Callous: 9 items; Unemotional: 5 items; Uncaring: 8 items	83.2%	*n* = 149	16-19 years old	Callous: *M* = 7.91, *SD* = 4.06; Uncaring: *M* = 10.68, *SD* = 4.94; Unemotional: *M* = 8.56, *SD* = 2.78	---	---	---	---
Harenski et al. (2014) USA	Forensic	ICU - self-report	0-3	Total: 24 items	100%	*n* = 119	14-19 years old	Total ICU: *M* = 28.40, *SD* = 8.89	---	---	---	---
Hawes et al. (2014) Australia	Community	ICU – parent version	0-3	Total: 12 items; Callous: 7 items; Uncaring: 5 items	100%	*n* = 250	6-12 years old	Total ICU: *M* = 15.42, *SD* = 6.05; Callous: *M* = 5.68, *SD* = 4.10; Uncaring: *M* = 9.74, *SD* = 2.84;	---	---	---	---
He et al. (2023) China	Community	ICU – parent version	0-3	Total: 12 items; Callous: 7 items; Uncaring: 5 items	46.2%	*n* = 168	7-12 years old	Total ICU: *M* = 22.18, *SD* = 5.07; Callous: *M* = 10.64, *SD* = 2.98; Uncaring: *M* = 9.78, *SD* = 2.25;	---	---	---	---
Herpers et al. (2016) Netherlands	Clinical	ICU - self-report	0-3	Total: 24 items	63.9%	*n* = 1018	6 years	Total ICU: *M* = 28.80, *SD* = 11.20	---	---	---	---
Herpers et al (2019) USA	Clinical and community	ICU - self-report and parent version	0-3	Total: 24 items	100%	CD: *n* = 52<br>ASD: *n* = 52	12-19 years old	Self-report<br>CD<br>Total ICU: *M* = 30.70, *SD* = 10.00;<br>ASD<br>Total ICU: *M* = 24.80, *SD* = 7.50;<br>Parent report<br>CD<br>Total ICU: *M* = 38.90, *SD* = 9.40;<br>ASD<br>Total ICU: *M* = 28.00, *SD* = 8.40	100%	*n* = 24	12-19 years old	Self-report<br>Total ICU: *M* = 23.20, *SD* = 6.30;<br>Parent report<br>Total ICU: *M* = 16.00, *SD* = 7.00
Heynen et al. (2017) Germany	Forensic	ICU - self-report	0-3	Total: 24 items; Callous: 11 items;	100%	*n* = 156	15-20 years	Callous: *M* = 11.22, *SD* = 4.84; Uncaring: *M* = 9.36, *SD* = 4.32;	---	---	---	---

				Unemotional: 5 items; Uncaring: 8 items				Unemotional: *M* = 8.60, *SD* = 3.00				
Hitti et al. (2021) USA	Community	ICU - self-report	0-3	Total: 18 items	48.0%	*n* = 265	11-15 years	Total ICU: *M* = 10.26, *SD* = 7.92	---	---	---	---
Hodsoll et al. (2014) UK	Community (with and without DBD)	ICU - teacher version	0-3	Total: 24 items	100%	*n* = 32	8-16 years old	CP/CU+ Total ICU: *M* = 49.50, *SD* = 7.70; CP/CU- Total ICU: *M* = 27.80, *SD* = 9.20	100%	*n* = 17	8-16 years old	Total ICU: *M* = 16.70, *SD* = 7.10
Hostetler et al. (2024) Canada	Community (with and without DBD)	ICU – parent report	0-3	Total: 24 items	87%	ODD/CD: *n* = 24; ADHD: *n* = 6	10-19 years old	ODD/CD Total ICU: *M* = 35.67, *SD* = 9.15; ADHD Total ICU: *M* = 21.67, *SD* = 6.80;	87%	*n* = 24	10-19 years old	Total ICU: *M* = 19.88, *SD* = 9.63
Howard et al. (2012) USA	Forensic	ICU - self-report	0-3	Total: 22 items	100%	*n* = 88	13-18 years old	Total ICU: *M* = 23.23, *SD* = 7.85	---	---	---	---
Hoyniak et al. (2018) USA	Community	ICU - parent version	0-3	Total: 22 items	14.8%	*n* = 27	2-5 years	Total ICU: *M* = 16.46, *SD* = 5.48	---	---	---	---
Hulvershorn et al. (2013) USA	Community	ICU - self-report	0-3	Total: 24 items; Callous: 11 items; Unemotional: 5 items; Uncaring: 8 items	74.0%	Psychopathology diagnosis: *n* = 19	10-14 years	Callous: *M* = 9.20, *SD* = 4.80; Uncaring: *M* = 11.0, *DP* = 5.10; Unemotional: *M* = 7.20, *SD* = 2.10	61.0%	*n* = 18	10-14 years	Callous: *M* = 5.80, *SD* = 4.20; Uncaring: *M* = 5.10, *DP* = 2.80; Unemotional: *M* = 6.00, *SD* = 2.10
Hwang et al. (2016) USA	Community (with and without DBD)	ICU - parent version	0-3	Total: 24 items	62.9%	DBD: *n* = 35	*Mean* = 13.88-14.81 years	Disruptive behavior and low CU Total ICU: *M* = 33.12, *SD* = 5.68; Disruptive behavior and high CU Total ICU: *M* = 48.11, *SD* = 5.67	53.6%	*n* = 28	*Mean* = 13.88-14.81 years	Total ICU: *M* =. 15.58, *SD* = 6.76
Hwang et al. (2021) UK	Community	ICU - self-report	0-3	Total: 22 items	50.6%	*n* = 437	11-14 years old	Total ICU: *M* = 21.28, *SD* = 7.88	---	---	---	---
Johander et	Community	ICU - self-	0-3	Total: 24	53.1%	*n* = 277	*Mean*	Total ICU: *M* = 36.48, *SD*	---	---	---	---

al. (2022) Finland	y	report		items;			= 12.93 years	= 15.12				
Johnson et al. (2014) USA	Forensic	ICU - self-report	0-3	Total: 22 items; Callous: 9 items; Unemotional: 5 items; Uncaring: 8 items	100%	*n* = 50	16.08 years old	Total ICU: *M* = 29.27, *SD* = 9.85; Callous: *M* = 7.54, *SD* = 4.38; Uncaring: *M* = 10.97, *SD* = 4.98; Unemotional: *M* = 8.08, *SD* = 3.22	---	---	---	---
Kahn et al. (2016) USA	Community	ICU - self-report and parent version	0-3	Total: 10 items;	52.0%	*n* = 115	*Mean* = 13.97 years	Low chaos - Self-report Total ICU: *M* = 5.43, *SD* = 3.68; Parent report Total ICU: *M* = 5.45, *SD* = 3.80; High chaos - Self-report Total ICU: *M* = 7.39, *SD* = 4.56; Parents Total ICU: *M* = 6.13, *SD* = 4.05	---	---	---	---
Kahn et al. (2017) USA	Community	ICU - self-report	0-3	Total: 24 items	100%	*n* = 112	12-20 years	Total ICU: *M* = 28.50, *SD* = 7.72	---	---	---	---
Kaouar et al. (2023) Australia	Community	ICU – parent version	0-3	Total: 24 items	77%	*n* = 135	2-7 years old	Total ICU: *M* = 28.85, *SD* = 9.24	---	---	---	---
Kauten et al. (2015) USA	Clinical	ICU - self-report	0-3	Total: 24 items	84.9%	*n* = 153	16-18 years old	Total ICU: *M* = 27.54, *SD* = 8.04	---	---	---	---
Kavish et al. (2017) USA	Community	ICU - self-report	0-3	Callousness: 11 items; Uncaring: 8 items; Unemotional: 5 items;	55.0%	*n* = 387	12 years	Callousness: *M* = 18.48, *SD* = 4.18 Uncaring: *M* = 15.92, *SD* = 4.80 Unemotional: *M* = 12.95, *SD* = 2.75	---	---	---	---
Kemp et al. (2022) Cyprus	Community	ICU - self-report	0-3	Total: 22 items	50.7%	Boys: *n* = 2375 Girls: *n* = 2308	11-17 years old	Boys Total ICU: *M* = 21.90, *SD* = 8.45; Girls Total ICU: *M* = 17.18, *SD* = 7.65	---	---	---	---
Kemp	Forensic	ICU - self-	0-3	Total: 22	100%	*n* = 1216	13-17	Justice Involved	100%	*n* =	13-17	School – self-report

et al. (2023) USA	and Community	report, parent and teacher version		items			years old	Self-Reported Total ICU: *M* = 26.28, *SD* = 8.08;		289	years old	Total ICU: *M* = 18.93, *SD* = 8.04; School – parent report Total ICU: *M* = 17.26, *SD* = 9.92; School – teacher report Total ICU: *M* = 21.31, *SD* = 13.54
Kemp et al. (2024) USA	Community	ICU – self-report	0-3	Total: 22 items	51%	*n* = 4683	11-17 years old	Boys Total ICU: *M* = 21.90, *SD* = 8.45; Girls Total ICU: *M* = 17.18, *SD* = 7.65;	---	---	---	---
Kerig et al. (2012) USA	Forensic	ICU - self-report	0-3	Total: 22 items; Callous: 9 items; Unemotional: 5 items; Uncaring: 8 items	75.4%	*n* = 276	11-18 years old	Boys Total ICU: *M* = 23.44, *SD* = 9.41; Unemotional: *M* = 7.97, *SD* = 3.26; Uncaring: *M* = 6.15, *SD* = 3.93; Callous: *M* = 9.32, *SD* = 4.47; Girls Total ICU: *M* = 24.65, *SD* = 3.10; Unemotional: *M* = 7.38, *SD* = 3.10; Uncaring: *M* = 7.18, *SD* = 4.68; Callous: *M* = 10.09, *SD* = 4.62	---	---	---	---
Kerig & Modrowski (2020) USA	Forensic	ICU - self-report	0-3	Total: 22 items	74.0%	*n* = 782	12-19 years old	Boys Total ICU: *M* = 27.10, *SD* = 8.24 Girls Total ICU: *M* = 27.04, *SD* = 9.17;	---	---	---	---
Kilshaw et al. (2022) USA	Forensic	ICU – self-report	0-3	Total: 24 items	80.6%	*n* = 170	12.52-17.96 years old	Boys Total ICU: *M* = 28.11, *SD* = 8.85: Girls Total ICU: *M* = 25.79, *SD* = 9.37	---	---	---	---
Kimonis et al. (2007)	Forensic	ICU - self-report	0-3	Total: 24 items	100%	*n* = 88	13-18 years	Total ICU: *M* = 23.23, *SD* = 7.85	---	---	---	---

USA							old					
Al et al. (2008) USA	Forensic	ICU - self-report	0-3	Total: 24 items; Unemotional: 5 items; Callous: 11 items; Uncaring: 8 items	75.8%	*n* = 248	12-20 years	Total ICU: *M* = 23.96, *SD* = 9.41; Unemotional: *M* = 7.50, *SD* = 3.03; Callous: *M* = 5.29, *SD* = 4.82; Uncaring: *M* = 8.68, *SD* = 5.16	---	---	---	---
Kimonis et al. (2011) USA	Community	ICU - self-report	0-3	Total: 24 items	100%	*n* = 373	*Mean* = 16.43 years	Total ICU: *M* = 27.60, *SD* = 8.64	---	---	---	---
Kimonis et al. (2013) USA	Forensic	ICU - self-report	0-3	Total: 22 items; Callous: 9 items; Unemotional: 5 items; Uncaring: 8 items	100%	*n* = 227	12-19 years old	Total ICU: *M* = 25.74, *SD* = 10.12; Uncaring: *M* = 11.18, *SD* = 5.30; Unemotional: *M* = 8.26, *SD* = 2.90; Callous: *M* = 6.31, *SD* = 4.55	---	---	---	---
Kimonis et al. (2016) Australia	Forensic	ICU - self-report	0-3	Total: 22 items; Callous: 9 items; Unemotional: 5 items; Uncaring: 8 items	100%	*n* = 232	14-18 years old	Total ICU: *M* = 28.56, *SD* = 8.21; Uncaring: *M* = 12.49, *SD* = 4.29; Unemotional: *M* = 8.95, *SD* =2.59 Callous: *M* = 7.12, *SD* = 4.11	---	---	---	---
Kimonis et al. (2017) USA	Forensic	ICU - self-report	0-3	Total: 22 items; Callous: 9 items; Unemotional: 5 items; Uncaring: 8 items	100%	*n* = 227	12-19 years old	Total ICU: *M* = 24.54, *SD* = 9.23; Uncaring: *M* = 11.13, *SD* = 5.26; Unemotional: *M* = 7.26, *SD* = 2.82; Callous: *M* = 8.87, *SD* = 4.91	---	---	---	---
Kimonis et al. (2018) USA	Community	ICU - self-report	0-3	Total: 22 items; Callous: 9 items; Unemotional: 5 items; Uncaring: 8	100%	*n* = 376	14-17 years old	Total ICU: *M* = 25.45, *SD* = 6.52; Uncaring: *M* = 10.85, *SD* = 4.95; Callous: *M* = 6.03, *SD* = 3.84; Unemotional: *M* = 8.41,	---	---	---	---

				items				*SD* = 2.57				
Kimonis et al. (2019a) Australia	Forensic	ICU - self-report	0-3	Total: 22 items	100%	*n* = 232	14-18 years old	Total ICU: *M* = 28.33, *SD* = 8.07	---	---	---	---
Kimonis et al. (2019b) USA	Community	ICU - parent version	0-3	Total: 22 items	87.0%	*n* = 23	3-6 years	Pretreatment Total ICU: *M* = 40.67, SD = 8.75; Post direct interaction with child Total ICU: *M* =35.59, *SD* = 8.24; Post direct interaction parents Total ICU: *M* = 29.99, *SD* = 8.07; Post treatment Total ICU: *M* = 29.91, *SD* = 10.16; 3 month follow up Total ICU: *M* = 29.74, *SD* = 11.93	---	---	---	---
Kimonis et al. (2023) Australia	Clinical (with and without DBD)	ICU - parent version	0-3	Total: 24 items	66.9%	CD: *n* = 47 CD +CU: *n* = 25	*Mean* = 3.64 years	CD Total ICU: *M* = 23.22, *SD* = 5.86; CD + CU Total ICU: *M* = 37.17, *SD* = 4.83	66.9%	*n* = 28	*Mean* = 3.64 years	Total ICU: *M* = 16.32, *SD* = 4.16
Kimonis et al. (2025) Australia	Community	ICU – parent version	0-3	Total: 24 items	78%	*n* = 87	*Mean* = 4.88 years	Total ICU: *M* = 35.45, *SD* = 9.50;	---	---	---	---
Kleberg et al. (2021) Sweden	Clinical	ICU - parent version	0-3	Total: 24 items	74.7 %	*n* = 71	*Mean* = 10.46 years	Total ICU: *M* = 26.27, *SD* = 4.91	---	---	---	---
Kochanska et al. (2013) USA	Community	ICU - parent version	0-3	Total: 24 items	50.0%	*n* = 102	3-8 years	Total ICU: *M* = 19.20, *SD* = 7.68	---	---	---	---
Kofler et al. (2022) USA	Community	ICU - parent version	0-3	Total: 24 items	47.8%	*n* = 245	8-11 years old	Boys Total ICU: *M* = 28.60, *SD* = 7.24; Girls Total ICU: *M* = 25.80, *SD* = 8.62	---	---	---	---
Koh et al. (2024)	Community	ICU – parent	0-3	Total: 24 items	53.9%	*n* = 899	2-8 years	Total ICU: *M* = 19.14, *SD* = 13.00	---	---	---	---

Australia		report					old					
Kongerslev et al (2015) Denmark	Forensic	ICU - self-report	0-3	Total: 24 items; Callous: 11 items; Unemotional: 5 items; Uncaring: 8 items;	100%	*n* = 80	15-18 years old	ICU Total: *M* = 33.88, *SD* = 11.61; Uncaring: *M* = 15.08, *SD* = 4.41; Callous: *M* = 11.65, *SD* = 5.65; Unemotional: *M* = 7.15, *SD* = 3.20	---	---	---	---
Kumsta et al. (2012) Romania and UK	Clinical	ICU - self-report and parent version	0-3	Total: 24 items; Callous: 11 items; Unemotional: 5 items; Uncaring: 8 items;	100%	*n* = 252	15 years old	Self-report Total ICU: *M* = 20.39, *SD* = 7.50; Callous: *M* = 5.27, *SD* = 3.40; Uncaring: *M* = 8.26, *SD* = 4.00; Unemotional: *M* = 6.57, *SD* = 2.70; Parent report ICU Total: *M* = 26.07, *SD* = 13.2; Callous: *M* = 7.75, *SD* = 6.30; Uncaring: *M* = 12.12, *SD* = 5.90; Unemotional: *M* = 6.20, *SD* = 3.60	---	---	---	---
Kurek et al. (2019) Australia	Community	ICU - self-report	0-3	Total: 12 items	49.5%	*n* = 709	13-17 years old	Total ICU: *M* = 22.92, *SD* = 2.28	---	---	---	---
Kyranides et al. (2018) Cyprus	Community	ICU - parent version	0-3	Total: 24 items	Prevention school = 47.9% School 1 and 2 = 49.5%	*n* = 304	*Mean* = 7.82-7.91 years old	School prevention time 1 Total ICU: *M* = 25.22, *SD* = 8.61 School 1 period 1 Total ICU: *M* = 24.51, *SD* = 7.55; School 2 period 1 Total ICU: *M* = 24.61, *SD* = 8.11	---	---	---	---
Lam et al. (2021) USA	Community	ICU - parent version	0-3	Total: 24 items	49.1%	*n* = 29	10-14 years old	Total ICU: *M* = 4.29, *SD* = 3.46	---	---	---	---
Latzman et al. (2016) USA	Clinical	ICU - self-report	0-3	Total: 22 items	46.0%	*n* = 81	12-20 years old	Refuge camp Total ICU: *M* = 31.28, *SD* = 8.65;	---	---	---	---

								No refugee camp<br>Total ICU: *M* = 25.38, *SD* = 8.77				
Lau & Marsee (2013) USA	Community	ICU - self-report	0-3	Total: 24 items	49.0%	*n* = 139	11-17 years old	Total ICU: *M* = 21.79, *SD* = 7.75	---	---	---	---
Lee-Rowland et al. (2020) USA	Clinical	ICU - self-report	0-3	Total: 24 items	16.9%	*n* = 219	16-19 years old	Total ICU: *M* = 27.32, *SD* = 8.62	---	---	---	---
Levy et al. (2022) Canada	Clinical	ICU - parent version	0-3	Total: 24 items	84.0%	*n* = 31	7-17 years old	Total ICU: *M* = 32.00, *SD* = 9.57	---	---	---	---
Liu & Guo (2024) China	Community	ICU – self-report	1-4	Total: 21 items	49.3%	*n* = 681	*Mean* = 12.29 years old	Total ICU: *M* = 39.69, *SD* = 9.03	---	---	---	---
Lopez-Romero et al. (2015a) Spain	Forensic	ICU - self-report	0-3	Total: 24 items; Callous: 11 items; Unemotional: 5 items; Uncaring: 8 items	72.5%	*n* = 324	*Mean* = 16.13 years old	Total ICU: *M* = 31.37, *SD* = 11.06; Callous: *M* = 12.95, *SD* = 5.48; Uncaring: *M* = 7.01, *SD* = 2.98; Unemotional: *M* = 13.94, *SD* = 5.85	---	---	---	---
Lopez-Romero et al. (2015b) Spain	Community	ICU - self-report and parent version	0-3	Total: 24 items; Callous: 11 items; Unemotional: 5 items; Uncaring: 8 items	68.8%	*n* = 138	12-17 years old	Parents<br>Total ICU: *M* = 24.78, *SD* = 10.00; Callous: *M* = 5.52, *SD* = 4.69; Uncaring: *M* = 13.55, *SD* = 5.62; Unemotional: *M* = 5.71, *SD* = 2.82;<br>Self-report<br>Total ICU: *M* = 24.65, *SD* = 8.36; Callous: *M* = 6.91, *SD* = 4.10; Uncaring: *M* = 10.39, *SD* = 4.80; Unemotional: *M* = 7.35, *SD* = 2.77	---	---	---	---

Lorber et al. (2011) USA	Community	ICU - self-report	0-3	Total: 24 items	75.0%	*n* = 76	10-19 years old	ICU Total: *M* = 31.95, SD = 8.81	---	---	---	---
Lozier et al. (2014) USA	Community (with and without DBD)	ICU - self-report	0-3	Total: 24 items	53.3%	*n* = 30	10-17 years old	CP<br>Total ICU: *M* = 44.28, *SD* = 8.31	62.5%	*n* = 16	10-17 years old	Total ICU: *M* = 25.50, *SD* = 6.66
Lui et al. (2016) China	Clinical	ICU - self-report	0-3	Total: 24 items	68.0%	*n* = 103	16-18 years old	Total ICU: *M* = 25.74, *SD* = 7.95	---	---	---	---
Lui et al. (2017) USA	Clinical	ICU - self-report	0-3	Total: 24 items; Callous: 11 items; Unemotional: 5 items; Uncaring: 8 items	85.2%	*n* = 209	16-19 years old	Total ICU: *M* = 27.83, *SD* = 7.78;<br>Callous: *M* = 7.3, *SD* = 3.89;<br>Uncaring: *M* = 11.75, *SD* = 4.80;<br>Unemotional: *M* = 8.28, *SD* = 2.66	---	---	---	---
Lui et al. (2019) USA	Clinical	ICU - self-report and parent version	0-3	Total: 24 items	83.9%	*n* = 85	16-18 years old	Self-report - T0 – emotion processing skills training<br>Total ICU: *M* = 29.44, *SD* = 7.50;<br>T1 – emotion processing skills training<br>Total ICU: *M* = 26.38, *SD* = 8.53;<br>T2 – emotion processing skills training<br>Total ICU: *M* = 28.10, *SD* = 8.91;<br>T3 – emotion processing skills training Total ICU: *M* = 19.68, *SD* = 4.70<br>Parents - T0 – emotion processing skills training<br>ICU Total: *M* = 33.59, *SD* = 13.03;<br>T3 – emotion processing skills training<br>ICU Total: *M* = 28.62, *SD* = 12.13	83.9%	*n* = 124	16-18 years old	Self-report - T0 – usual treatment<br>Total ICU: *M* = 28.41, *SD* = 9.49;<br>T1 - usual care<br>Total ICU: *M* = 28.72, *SD* = 8.83;<br>T2 - usual care<br>ICU Total: *M* = 34.79, *SD* = 7.59;<br>T3 - usual treatment<br>Total ICU: *M* = 22.48, *SD* = 6.75;<br>Parents - T0 – usual care<br>Total ICU: *M* = 34.71, *SD* = 10.46;<br>T3 - usual treatment<br>Total ICU: *M* = 16.97, *SD* = 5.46
Lynch et al. (2024) USA	Community	ICU – parent version	0-3	Total: 24 items	43.4%	*n* = 55	7-10 years old	Total ICU: *M* = 15.61, *SD* = 8.70	---	---	---	---
Ma et al.	Communit	ICU –	1-4	Total: 24	48.6%	*n* = 284	8-12	Mother report	---	---	---	---

(2024)<br>China	y	parent<br>version		items			years<br>old	Total ICU: *M* = 47.02, *SD* = 6.99;<br>Father report<br>Total ICU: *M* = 47.72, *SD* = 6.87				
Maneiro et al. (2019)<br>Spain	Clinical	ICU - self-report	0-3	Total: 24 items	59.3%	*n* = 145	11-19 years old	Low risk<br>Total ICU: *M* = 20.04, *SD* = 5.19;<br>Medium risk<br>Total ICU: *M* = 21.63, *SD* = 6.13;<br>High risk<br>Total ICU: *M* = 26.26, *SD* = 6.20	---	---	---	---
Mann et al. (2018)<br>USA	Communit<br>y	ICU - self-report	0-3	Total: 24 items	50.0%	*n* = 824	9-12 years old	Total ICU: *M* = 45.12, *SD* = 7.44	---	---	---	---
Marsee et al. (2014b)<br>USA	Communit<br>y	ICU - self-report	0-3	Total: 24 items	49.0%	*n* = 141	11-17 years old	Total ICU: *M* = 21.84, *SD* = 7.84	---	---	---	---
Marsee et al. (2014a)<br>USA	Communit<br>y	ICU - self-report	0-3	Total: 24 items	43.6%	*n* = 303	11-18 years old	Low reactive<br>Total ICU: *M* = 20.31, *SD* = 7.02;<br>High reactive<br>Total ICU: *M* = 24.52, *SD* = 9.10;<br>Combined<br>Total ICU: *M* = 31.70, *SD* = 9.35	---	---	---	---
Martin-Key et al. (2020)<br>UK	Forensic and Communit<br>y	ICU - self-report	0-3	Total: 24 items	0%	CD: *n* = 23	13-18 years old	Total ICU: *M* = 28.00, *SD* = 8.59	0%	*n* = 29	13-18 years old	Total ICU: *M* = 19.19, *SD* = 6.95
Martin-Key et al. (2021)<br>UK	Forensic and Communit<br>y	ICU - self-report	0-3	Total: 24 items	48.9%	CD: *n* = 45	13-18 years old	Boys<br>Total ICU: *M* = 30.23, *SD* = 7.97<br>Girls<br>Total ICU: *M* = 26.65, *SD* = 9.11	51.0%	*n* = 51	13-18 years old	Boys<br>Total ICU: *M* = 22.88, *SD* = 6.08<br>Girls<br>Total ICU: *M* = 17.96, *SD* = 6.47
Masi et al. (2011)<br>USA	Clinical	ICU - self-report and parent version	0-3	Total: 24 items	73.7%	DBD: *n* = 38	6-16 years old	Non-responsive – self-report<br>Total ICU: *M* = 30.00, *SD* = 9.90<br>Responsive – self-report<br>Total ICU: *M* = 23.90, *SD* = 7.10;	---	---	---	---

								Non-responsive – parents Total ICU: *M* = 34.50, *SD* = 8.00; Responsive – parents Total ICU: *M* = 32.70, *SD* = 9.40				
Masi et al. (2018) USA	Clinical	ICU - self-report	0-3	Total: 24 items	88.9%	*n* = 144	8.7 years	CU time 1 Total ICU: *M* = 27.58, *SD* = 5.38; CU time 2 Total ICU: *M* = 23.97, *SD* = 6.11; CU time 3 - Total ICU: *M* = 21.55, *SD* = 6.32; CU time 4 Total ICU: *M* = 23.02, *SD* = 6.58	---	---	---	---
Matlasz et al. (2020) USA	Forensic	ICU - self-report	0-3	Total: 24 items	100%	*n* = 1216	13-17 years old	Total ICU: *M* = 26.27, *SD* = 8.08	---	---	---	---
Matlasz et al. (2023) USA	Community	ICU - self-report, parent and teacher version	0-3	Total: 24 items;	39.4%	*n* = 236	8-15 years	Self-report Total ICU: *M* = 18.99, *SD* = 8.10 Parent report Total ICU: *M* = 16.56, *SD* = 9.46 Teacher report Total ICU: *M* = 20.50, *SD* = 13.04	---	---	---	---
Mattos et al. (2017) USA	Clinical	ICU - parent version	0-3	Total: 24 items	49.0%	*n* = 59	12-18 years old	Total ICU: *M* = 32.87, *SD* = 12.27	---	---	---	---
McLoughlin et al. (2010) New Zealand	Community	ICU – Parent version	0-3	Total: 24 items	Low CU: 36.0% High CU:17.0%	*n* = 94	*Mean* = 10.85 years	Low CU Total ICU: *M* = 17.88, *SD* = 6.67; High CU Total ICU: *M* = 37.40, *SD* = 6.65	---	---	---	---
Mendez et al. (2020) USA	Forensic	ICU - self-report	0-3	Total: 24 items	74.0%	*n* = 829	12-19 years old	Girls Total ICU: *M* = 26.99, *SD* = 9.04; Boys Total ICU: *M* = 27.20, *SD* = 8.22	---	---	---	---
Milledge et	Communit	ICU- parent	0-3	Total: 24	81.6%	*n* = 147	*Mean*	Parents report	---	---	---	---

al. (2019) UK	y	and teacher version		items			= 5.17 years	Total ICU: *M* = 36.15, *SD* = 9.60; Teacher report Total ICU: *M* = 31.26, *SD* = 10.04				
Miller et al. (2013) USA	Clinic	ICU - parent and teacher version	0-3	Total: 24 items	90.9%	*n* = 11	7-11 years	Parent report Total ICU: *M* = 37.50, *SD* = 5.00; Teacher report Total ICU: *M* = 40.90, *SD* = 12.40	---	---	---	---
Miller & Marsee (2019) USA	Forensic	ICU - self-report	0-3	Total: 24 items	100%	*n* = 198	12-19 years old	Total ICU: *M* = 28.76, *SD* = 8.69	---	---	---	---
Mozley et al. (2018) USA	Forensic	ICU - self-report	0-3	Total: 24 items	74.2%	*n* = 380	12-19 years old	Total ICU: *M* = 29.30, *SD* = 6.51	---	---	---	---
Muñoz et al. (2008a) USA	Forensic	ICU - self-report	0-3	Total: 22 items	100%	*n* = 100	13-18 years old	Total ICU: *M* = 23.36, *SD* = 8.05	---	---	---	---
Muñoz et al. (2008b) USA	Forensic	ICU - self-report	0-3	Total: 22 items	100%	*n* = 85	13-18 years old	Total ICU: *M* = 23.02, *SD* = 7.70	---	---	---	---
Muñoz (2009) UK	Communit y	ICU - self-report	0-3	Total: 22 items	100%	*n* = 55	8-16 years old	Total ICU: *M* = 34.16, *SD* = 11.24	---	---	---	---
Muñoz et al. (2011) UK	Communit y	ICU - self-report	0-3	Total: 24 items	49.0%	Boys: *n* = 100 Girls: *n* = 101	11-12 years old	Boys Total ICU: *M* = 22.22, *SD* = 7.96; Girls Total ICU: *M* = 19.19, *SD* = 9.48	---	---	---	---
Muratori et al. (2016) Italy	Clinic	ICU - self-report	0-3	Total: 24 items	90.0%	*n* = 126	9-12 years old	ICU T0 Total ICU: *M* = 27.75, *SD* = 6.89; ICU T1 Total ICU: *M* = 24.47, *SD* = 7.13; ICU T2 ICU Total: *M* = 22.70, *SD* = 7.47	---	---	---	---
Muratori et al. (2019)	Clinical	ICU - self-report	0-3	Total: 24 items	93.0%	DBD: *n* = 55	9-10 years	Total ICU: *M* = 27.98, *SD* = 4.67	93.0%	*n* = 420	9-10 years	Total ICU: *M* = 28.57, *SD* = 7.70

Italy							old					
Muratori et al. (2021) Italy	Community	ICU - self-report	0-3	Total: 24 items	51.8%	*n* = 2959	10-14 years old	Total ICU: *M* = 23.00, *SD* = 8.30	---	---	---	---
Myers et al. (2018) USA	Forensic	ICU - self-report	0-3	Total: 24 items	100%	*n* = 2268	13-17 years old	ICU Total: *M* = 25.44, *SD* = 7.66	---	---	---	---
Nichols et al. (2015) USA	Clinical	ICU - parent version	0-3	Total: 19 items; Callous: 11 items Uncaring: 8 items	48.7%	*n* = 1855	3-5 years old	Callous: *M* = 4.49, *SD* = 3.24; Uncaring: *M* = 15.28, *SD* = 4.65	---	---	---	---
Normand et al (2023) USA	Community	ICU - parent version	0-3	Total: 24 items	67.3%	*n* = 330	*Mean* = 8.59 years old	Target Total ICU: *M* = 30.39, *SD* = 8.50; Friend Total ICU: *M* = 19.28, *SD* = 7.94	---	---	---	---
Nwafor et al. (2015) Spain	Community	ICU - self-report	0-3	Total: 22 items Callous: 9 items; Unemotional: 5 items; Uncaring: 8 items	51.5%	*n* = 295	14-16 years old	Boys Total ICU: *M* =19.56, *SD* = 8.73; Callous: *M* = 5.59, *SD* = 4.53; Uncaring: *M* = 7.19, *SD* = 4.53; Girls Total ICU: *M* = 19.55, *SD* = 8.54; Callous: *M* = 6.22, *SD* = 4.68; Uncaring: *M* = 5.57, *SD* = 3.81	---	---	---	---
Paciello et al. (2017) Italy	Clinical	ICU - self-report	0-3	Total: 24 items	85.6%	DBD: *n* = 90	11-18 years old	Total ICU: *M* = 27.57, *SD* = 10.57	---	---	---	---
Payot et al. (2025) Belgium	Community	ICU – parent version	0-3	Total: 18 items; Lack of conscience: 10 items; Unconcern about performance: 5 items; Lack of	54.6%	*n* = 260	3-9 years old	Total ICU = *M* = 11.62, *SD* = 7.00; Lack of conscience = *M* = 6.89, *SD* = 4.70; Unconcern about performance = *M* = 3.21, *SD* = 2.80; Lack of emotional expression = *M* = 1.51, *SD* = 1.50	---	---	---	---

				emotional expression: 3 items								
Pechorro et al. (2018) Portugal	Forensic	ICU - self-report	0-3	Total: 10 items<br>Total: 12 items	100%	*n* = 221	13-20 years old	ICU-12 (item 4): *M* = 0.92, *SD* = 0.88;<br>ICU-12 (item 6): *M* = 1.80, *SD* = 0.84;<br>ICU-12 (item 9): *M* = 1.34, *SD* = 0.97;<br>ICU-12 (item 11): *M* = 1.13, *SD* = 1.01;<br>ICU-12 (item 12): *M* = 1.28, *SD* = 0.89;<br>ICU-12 (item 18): *M* = 1.28, *SD* = 0.94;<br>ICU-12 (item 21): *M* = 1.00, *SD* = 0.86;<br>ICU-12 (item 5): *M* = 1.45, *SD* = 0.86;<br>ICU-12 (item 8): *M* = 0.96, *SD* = 0.85;<br>ICU-12 (item 16): *M* = 1.27, *SD* = 0.85;<br>ICU-12 (item 17): *M* = 1.33, *SD* = 0.82;<br>ICU-12 (item 24): *M* = 1.17, *SD* = 0.77;<br>ICU-10 (item 3): *M* = 1.36, *SD* = 0.98;<br>ICU-10 (item 5): *M* = 1.45, *SD* = 0.86;<br>ICU-10 (item 15): *M* = 1.02, *SD* = 0.83;<br>ICU-10 (item 16): *M* = 1.27, *SD* = 0.85;<br>ICU-10 (item 17): *M* = 1.33, *SD* = 0.82;<br>ICU-10 (item 23): *M* = 1.19, *SD* = 0.81;<br>ICU-10 (item 24): *M* = 1.17, *SD* = 0.77;<br>ICU-10 (item 7): *M* = 1.17, *SD* = 0.97;<br>ICU-10 (item 8): *M* = 0.96, *SD* = 0.85;<br>ICU-10 (item 11): *M* =	---	---	---	---

								1.13, *SD* = 1.01				
Pechorro et al. (2019) Portugal	Community	ICU - self-report	0-3	Total: 12 items	47.4%	*n* = 782	12-20 years	Total ICU: *M* = 22.20, *SD* = 9.32	---	---	---	---
Pederson et al. (2020) USA	Forensic	ICU - self-report	0-3	Total: 22 items	72.9%	*n* = 129	11-18 years old	Total ICU: *M* = 25.39, *SD* = 8.36	---	---	---	---
Perlstein et al. (2021) USA	Clinical	ICU - parent version	0-3	Total: 24 items	53 %	*n* = 162	*Mean* = 10.60-14.10 years	Total ICU: *M* = 27.10, *SD* = 11.40	---	---	---	---
Pihet et al. (2015) Switzerland	Clinical and Community	ICU - self-report	1-3	Total: 24 items; Callous: 11 items; Unemotional: 5 items Uncaring: 8 items	70.0%	Institutionalized boys: *n* = 111 Institutionalized girls: *n* = 47 Boys without CD: *n* = 55 Girls without CD: *n* = 24 Boys with CD: *n* = 56 Girls with CD: *n* = 23	*Mean* = 15.8 years	Boys Total ICU: *M* = 1.14, *SD* = 0.40; Unemotional: *M* = 1.65, *SD* = 0.70; Uncaring: *M* = 1.36, *SD* = 0.60; Callous: *M* = 0.74, *SD* = 0.40; Girls Total ICU: *M* = 1.07, *SD* = 0.40; Unemotional: *M* = 1.55, *SD* = 0.70; Uncaring: *M* = 1.30, *SD* = 0.60; Callous: *M* =0.68, *SD* = 0.50; Boys without CD Total ICU: *M* = 1.02, *SD* = 0.30; Unemotional: *M* = 1.68, *SD* = 0.50; Uncaring: *M* = 1.18, *SD* = 0.60; Callous: *M* = 0.60, *SD* = 0.30; Girls without CD Total ICU: *M* = 0.90, *SD* = 0.30; Unemotional: *M* =1.42, *SD* = 0.60; Uncaring: *M* = 1.19, *SD* = 0.60;	38%	Boys n = 147 Girls n = 242	*Mity*= 15.8 years	Boys Total ICU: *M* = 0.98, *SD* = 0.30; Unemotional: *M* = 1.51, *SD* = 0.50; Uncaring: *M* 1.21, *SD* = 0.40; Callous: *M* = 0.57, *SD* = 0.30; Girls Total ICU: *M* = 0.82, *SD* = 0.30; Unemotional: *M* = 1.41, *SD* = 0.60; Uncaring: *M* = 1.07, *SD* = 0.50; Callous: *M* = 0.36, *SD* = 0.30

								Callous: *M* = 0.46, *SD* = 0.30;<br>Boys with CD<br>Total ICU: *M* = 1.25, *SD* = 0.40;<br>Unemotional: *M* = 1.62, *SD* = 0.70;<br>Uncaring: *M* = 1.53, *SD* = 0.50;<br>Callous: *M* = 0.89, *SD* = 0.40;<br>Girls with CD<br>Total ICU: *M* = 1.24, *SD* = 0.50;<br>Unemotional: *M* = 1.69, *SD* = 0.60;<br>Uncaring: *M* = 1.42, *SD* = 0.70;<br>Callous: *M* = 0.90, *SD* = 0.60				
Piscitello et al. (2025) USA	Community	ICU – parent version	0-3	Total: 24 items	78.5%	*n* = 33	6-12 years old	Total ICU: *M* = 27.52, *SD* = 5.98;	---	---	---	---
Platje et al. (2018) Netherlands	Community	ICU - self-report	0-3	Total: 24 items	100%	*n* = 346	12-17 years old	Low CU/low CD<br>Total ICU: *M* = 19.56, *SD* = 4.27;<br>High CU/Low CD<br>Total ICU: *M* = 33.73, *SD* = 3.73;<br>Low CU/ High CD<br>Total ICU: *M* = 20.69, *SD* = 3.28;<br>High CU/high CD<br>Total ICU: *M* = 33.77, *SD* = 3.95	---	---	---	---
Portengen et al. (2021) Various country of Europe	Clinical	ICU - self-report	0-3	Total: 24 items;	CD: 82.1%<br><br>ADHD: 56.1%	CD: *n* = 145<br><br>ADHD: *n* = 123	7-18 years old	CD<br>ICU Total: *M* = 29.67, *SD* = 9.52;<br>ADHD<br>ICU Total: *M* = 23.91, *SD* = 7.59	60.0%	*n* = 170	8-26 years old	Total ICU: *M* = 20.97, *SD* = 6.92
Portnoy et al. (2020) China	Community	ICU - self-report	0-3	Total: 19 items;<br>Callous: 11 items;<br>Uncaring: 8	55.7 %	*n* = 414	3-12 years	Girls<br>Callous: *M* = 5.90, *SD* = 5.76<br>Uncaring: *M* = 9.23, *SD* = 5.77;	---	---	---	---

				items				Boys<br>ICU- Callous: *M* = 7.16, *SD* = 6.85;<br>Uncaring: *M* = 11.12, *SD* = 6.22				
Powell et al. (2024) USA	Community	ICU – parent version	0-3	Total: 24 items;	47%	*n* = 45	7-10 years old	Total ICU: *M* = 15.70, *SD* = 9.32;	---	---	---	---
Preston et al. (2021) USA	Clinical	ICU - self-report	0-3	Total: 24 items;<br>Callous: 11 items;<br>Unemotional: 5 items;<br>Uncaring: 8 items	Initial sample = 81.3%<br><br>Replicate sample = 79.6%	Initial sample: *n* = 236<br><br>Replicate sample: *n* = 274	18 years old	Initial sample<br>Total ICU: *M* = 26.54, *SD* = 9.05;<br>Callous: *M* = 7.49, *SD* = 4.18;<br>Uncaring: *M* = 10.02, *SD* = 6.09;<br>Unemotional: *M* = 9.03, *SD* = 2.79;<br>Replicate sample<br>Total ICU: *M* = 27.32, *SD* = 9.25;<br>Callous: *M* = 8.37, *SD* = 4.77;<br>Uncaring: *M* = 9.85, *SD* = 5.68;<br>Unemotional: *M* = 8.96, *SD* = 2.81	---	---	---	---
Priddis et al. (2014) Australia	Clinical	ICU - self-report	0-3	Total: 24 items;<br>Callous: 11 items;<br>Unemotional: 5 items;<br>Uncaring: 8 items	Aggressive: 80.7%<br><br>Non-aggressive: 73.1%	Aggressive: *n* = 31<br><br>Non-aggressive: *n* = 26	*Mean* = 8.97 years	Aggressive<br>Unemotional: *M* = 8.94, *SD* = 4.30;<br>Callous: *M* = 6.38, *SD* = 2.97;<br>Uncaring: *M* = 9.75, *SD* = 2.86;<br>Non-aggressive<br>Unemotional: *M* = 5.47, *SD* = 3.56;<br>Callous: *M* = 8.94, *SD* = 3.26;<br>Uncaring: *M* = 4.29, *SD* = 3.84	---	---	---	---
Puzzo et al. (2018) UK	Forensic and Community	ICU - self-report	0-3	Total: 24 items	100%	CD: *n* = 33	13-18 years old	CD/CU+<br>Total ICU: *M* = 38.60, *SD* = 5.30;<br>CD/CU-<br>Total ICU: *M* = 260.0, *SD* = 5.00	100%	*n* = 29	13-18 years old	Total ICU: *M* = 23.4, *SD* = 8.2

Rehder et al. (2017) USA	Community	ICU - self-report	0-3	Total: 24 items	49.1%	*n* = 893	5-7 years	Total ICU: *M* = 7.92, *SD* = 7.20	---	---	---	---
Rehder et al. (2021) USA	Community	ICU - parent version	0-3	Total: 24 items	49.1 %	*n* = 1166	5 years	Total ICU: *M* = 15.36, *SD* = 9.84	---	---	---	---
Rhoads et al. (2020) USA	Community	ICU - self-report	0-3	Total: 22 items; Callous: 9 items; Unemotional: 5 items; Uncaring: 8 items	100%	*n* = 30	10-17 years old	Total ICU: *M* = 35.82, *SD* = 13.63; Callous: *M* = 12.41, *SD* = 6.44; Unemotional: *M* = 8.73, *SD* = 2.49; Uncaring: *M* = 14.68, *SD* = 5.96	---	---	---	---
Rizeq et al. (2020) USA	Community	ICU - parent version	0-3	Total: 24 items	67.9%	*n* = 81	8-12 years old	Total ICU: *M* = 20.18, *SD* = 7.73	---	---	---	---
Roberts et al. (2018) USA	Community (with and without DBD)	ICU - parent version	0-3	Total: 24 items	100%	*n* = 70	11-16 years old	CP/low CU Total ICU: *M* = 33.61, *SD* = 7.39; CP/high CU Total ICU: *M* = 51.00, *SD* = 6.57	100%	*n* = 31	11-16 years old	Total ICU: *M* = 25.39, *SD* = 7.36
Roberts et al. (2020) UK	Community (with and without DBD)	ICU - self-report	0-3	Total: 24 items	100%	*n* = 81	11-16 years old	CP/low CU Total ICU: *M* = 33.46, *SD* = 6.76; CP/high CU Total ICU: *M* = 49.32, *SD* = 5.68	100%	*n* = 27	11-16 years old	Total ICU: *M* = 25.74, *SD* = 6.04
Robertson et al. (2020) USA	Community	ICU - self-report	0-3	Total: 24 items	100%	*n* = 1215	13-17 years old	Total ICU: *M* = 26.27, *SD* = 8.08	---	---	---	---
Robertson et al. (2021) USA	Forensic	ICU - self-report	0-3	Total: 24 items	100%	*n* = 1216	13-17 years old	Total ICU: *M* = 26.28, *SD* = 8.08	---	---	---	---
Romero & Alonso (2017) Spain	Community	ICU - self-report	0-4	Total: 24 items; Callous: 11 items; Uncaring: 8 items; Unemotional: 5 items	47.7%	*n* = 910	12-19 years old	Total ICU: *M* = 25.15, *SD* = 8.78; Callous: *M* = 8.66, *SD* = 5.31; Uncaring: *M* = 9.15, *SD* = 4.10; Unemotional: *M* = 7.14, *SD* = 3.25	---	---	---	---
Roose et al. (2013) USA	Community	ICU - self-report	0-3	Total: 24 items; Callous: 11	80.0%	*n* = 79	14-18 years old	Total ICU: *M* = 26.47, *SD* = 9.79; Callous: *M* = 9.14, *SD* =	---	---	---	---

				items; Unemotional: 5 items Uncaring: 8 items				4.80; Uncaring: *M* = 9.75, *SD* = 4.78; Unemotional: *M* = 7.58, *SD* = 3.73				
Sanchez & Cooley (2024) USA	Community	ICU – teacher version	0-3	Total: 12 items	51.1%	*n* = 284	7-12 years old	Total ICU: *M* = 7.32, *SD* = 5.96	---	---	---	---
Scavenius et al. (2020) Denmark	Community	ICU-parent version	0-3	Total: 12 items; Callous: 7 items; Uncaring: 5 items	48.8%	*n* = 576	*Mean* = 8.6-18.7 years old	Pretreatment<br>Girls<br>Total ICU: *M* = 12.45, *SD* = 6.93;<br>Uncaring: *M* = 6.90, *SD* = 3.24;<br>Callous: *M* = 5.53, *SD* = 4.28;<br>Boys<br>Total ICU: *M* = 12.71, *SD* = 6.60;<br>Uncaring: *M* = 7.28, *SD* = 3.10;<br>Callous: *M* = 5.46, *SD* = 4.11;<br>Post-treatment<br>Girls<br>Total ICU: *M* = 10.60, *SD* = 6.82;<br>Uncaring: *M* = 6.37, *SD* = 3.29;<br>Callous: *M* = 4.26, *SD* = 4.05;<br>Boys<br>Total ICU: *M* = 10.89, *SD* = 6.19;<br>Uncaring: *M* = 6.49, *SD* = 3.14;<br>Callous: *M* = 4.40, *SD* = 3.73	---	---	---	---
Schwenck et al (2017) Germany	Clinic and community	ICU - self-report and parent version	0-3	Total: 24 items	100%	CP: *n* = 19	*Mean* = 11.07-17.76 years old	Self-report<br>Total ICU: *M* = 25.89, *SD* = 8.38;<br>Parent report<br>Total ICU: *M* = 30.37, *SD* = 13.04	100%	*n* = 24	*Mean* =11.07-17.76 years	Self-report<br>Total ICU: *M* = 21.52, *SD* = 5.87<br>Parent report<br>Total ICU: *M* = 16.33, *SD* = 5.87

Sebastian et al. (2014) UK	Community (with and without DBD)	ICU - teacher version	0-3	Total: 24 items	100%	*n* = 55	10-16 years old	CP/low CU Total ICU: *M* = 35.35, *SD* = 7.87; CP/ high CU Total ICU: *M* = 53.35, *SD* = 5.60	100%	*n* = 55	10-16 years	Total ICU: *M* = 24.00, *SD* = 5.81
Sebastian et al. (2016) UK	Community(with and without DBD)	ICU - teacher version	0-3	Total: 24 items	100%	*n* = 89	10-16 years old	CP/low CU Total ICU: *M* = 34.97, *SD* = 6.60; CP/high CU Total ICU: *M* = 52.45, *SD* = 6.56	100%	*n* = 29	10-16 years	Total ICU: *M* = 25.17, *SD* = 4.94
Sharf et al. (2014) USA	Forensic	ICU - self-report	0-3	Total: 24 items; Callous: 11 items; Unemotional: 5 items; Uncaring: 8 items	100%	*n* = 238	14-19 years old	Total ICU: *M* = 30.79, *SD* = 8.24; Uncaring: *M* = 12.36, *SD* = 4.24; Unemotional: *M* = 8.93, *SD* = 2.51; Callous: *M* = 7.00, *SD* = 4.05	---	---	---	---
Sharp & Vanwoerden (2014) USA	Clinical	ICU - self-report	0-3	Total: 24 items; Callous: 11 items; Unemotional: 5 items; Uncaring: 8 items	38.5%	*n* = 342	12-17 years old	Total ICU: *M* = 24.35, *SD* = 9.12; Unemotional: *M* = 7.78, *SD* = 3.61; Uncaring: *M* = 9.45, *SD* = 4.69; Callous: *M* =7.12, *SD* = 4.18	---	---	---	---
Simpson et al. (2013) USA	Forensic	ICU - self-report	0-3	Total: 24 items	100%	*n* = 58	15-18 years old	Total ICU: *M* = 28.34, *SD* = 9.79	---	---	---	---
Sourander et al. (2016) Finland	Community (with DBD)	ICU - parent version	0-3	Total: 24 items; Callous: 11 items; Unemotional: 5 items; Uncaring: 8 items	61.2%	*n* = 232	4 years old	Baseline Total ICU: *M* = 24.50, *SD* = 0.60; Callous: *M* = 8.20, *SD* = 0.30; Uncaring: *M* = 13.10, *SD* = 0.30; Unemotional: *M* = 3.20, *SD* = 0.20; 6 months Total ICU: *M* = 20.30, *SD* = 0.60; Callous: *M* = 6.20, *SD* = 0.30; Uncaring: *M* = 11.20, *SD* =	37.5%	*n* = 232	4 years	Baseline ICU Total: *M* = 23.90, *SD* = 0.60; Callous: *M* = 8.00, *SD* = 0.30; Uncaring: *M* = 13.0,0 *SD* = 0.30; Unemotional: *M* = 2.90, *SD* = 0.20; 6 months Total ICU: *M* = 21.40, *SD* = 0.60; Callous: *M* = 6.80, *SD* = 0.30; Uncaring: *M* = 11.60, *SD* =

								0.30; Unemotional: *M* = 2.90, *SD* = 0.20; 12 months Total ICU: *M* = 20.10, *SD* = 0.60; Callous: *M* = 6.00, *SD* = 0.30; Uncaring: *M* = 11.10, *SD* = 0.30; Unemotional: *M* = 3.00, *SD* = 0.20				0.30; Unemotional: *M* =3.00, *SD* = 0.20; 12 months Total ICU: *M* = 21.00, *SD* = 0.60; Callous: *M* = 6.80, *SD* = 0.30; Uncaring: *M* = 11.30, *SD* = 0.30; Unemotional: *M* = 2.90, *SD* = 0.20
Speck et al. (2024) USA	Forensic	ICU - self-report	0-3	Total: 24 items	100%	*n* = 766	13-17 years old	Total ICU: *M* = 25.96, *SD* = 8.11	---	---	---	---
Stickle et al. (2009) USA	Forensic	ICU - self-report	0-3	Total: 24 items	0%	*n* = 150	11-17 years old	Total ICU: *M* = 46.50, *SD* = 6.04	---	---	---	---
Stoppelbein et al. (2021) USA	Community	ICU - teacher version	0-3	Total: 24 items; Callous: 11 items; Unemotional: 5 items; Uncaring: 8 items	100%	*n* = 91	6-14 years old	Callous: *M* = 10.03, *SD* = 5.60; Uncaring: *M* = 15.48, *SD* = 4.40; Unemotional: *M* = 6.15, *SD* = 3.90	---	---	---	---
Sukhodolsky et al. (2022) USA	Community	ICU - parent version	0-3	Total: 24 items	69.0%	*n* = 58	8-16 years old	Total ICU: *M* = 32.89, *SD* = 10.39	---	---	---	---
Szabó et al. (2020) Hungary	Forensic	ICU - self-report	0-3	Total: 22 items	100%	*n* = 117	14-19 years old	ICU Total: *M* = 25.30, *SD* = 9.17	---	---	---	---
Tabares et al. (2025) Colombia	Forensic	ICU - self-report	0-3	Total: 24 items; Callous: 11 items; Unemotional: 5 items; Uncaring: 8 items	82.2%	*n* = 376	14-24 years old	ICU Total: *M* = 28.84, *SD* = 8.41; Callous: *M* = 14.29, *SD* = 6.21; Uncaring: *M* = 8.00, *SD* = 4.62; Unemotional: *M* = 7.55, *SD* = 2.81	---	---	---	---
Tan et al. (2025) China	Community	ICU – teacher version	0-3	Total: 11 items	50.8%	*n* = 520	3-6 years old	ICU Total: *M* = 22.66, *SD* = 4.51;	---	---	---	---
Thogersen	Clinical	ICU -	0-3	Total: 12	50.9%	DBD: *n* =	*Mean*	ICU Total: *M* = 12.12, *SD*	50.9%	*n* =	14.4	Total ICU: *M* = 15.86, *SD*

et al. (2021) Denmark		parent version		items		113	= 14.4 years old	= 6.20;		218	years	= 6.80
Thomson et al. (2024) USA	Clinical	ICU - self-report	0-3	Total: 24 items;	70.8%	*n* = 161	12-17 years old	Total ICU: *M* = 23.08, *SD* = 8.67;	---	---	---	---
Thornberg et al. (2017) Sweden	Community	ICU - self-report	0-3	Total: 12 items; Callous: 4 items; Unemotional: 5 items; Uncaring: 3 items	52.0%	*n* = 381	*Mean* = 10.0-13.5 years old	Total ICU: *M* = 10.68, *SD* = 4.56; Callous: *M* = 1.44, *SD* = 2.48; Uncaring: *M* = 2.10, *SD* = 1.77; Unemotional: *M* = 7.10, *SD* = 2.85	---	---	---	---
Thornberg et al. (2025) Sweden	Community	ICU - self-report	0-3	Total: 12 items; Callous: 4 items; Unemotional: 5 items; Uncaring: 3 items	45%	*n* = 706	10-20 years old	Total ICU: *M* = 23.76, *SD* = 5.04; Callous: *M* = 5.68, *SD* = 2.36; Uncaring: *M* = 5.49, *SD* = 2.01; Unemotional: *M* = 12.50, *SD* = 3.00	---	---	---	---
Thornton et al. (2019) USA	Forensic	ICU - self-report	0-3	Total: 24 items	100%	*n* = 1216	13-17 years old	Total ICU: *M* =26.29, *SD* = 8.11	---	---	---	---
Truedsson et al. (2019) Sweden	Community	ICU – parent and teacher version	0-3	Total: 24 items	100%	*n* = 867	4-6 years	Parent report Total ICU: *M* = 19.80, *SD* = 9.00; Teacher report ICU Total: *M* = 22.30, *SD* = 8.70	---	---	---	---
Urben et al. (2018) Switzerland	Forensic	ICU - self-report	0-3	Total: 24 items	100%	*n* = 111	12-18 years old	ICU Total: *M* = 29.28, *SD* = 7.44	---	---	---	---
Vaughn et al. (2011) USA	Community	ICU - Teacher version	0-3	Total: 24 items; Callous: 11 items; Unemotional: 5 items; Uncaring: 8 items	57.0%	*n* = 432	12-14 years old	Total ICU: *M* = 54.30, *SD* = 9.86; Callous: *M* = 26.58, *SD* = 5.83 Uncaring: *M* = 14.10, *SD* = 4.18; Unemotional: *M* = 13.62, *SD* = 3.26	---	---	---	---
Vaughan et al. (2025) USA	Forensic	ICU - self-report	0-3	Total: 24 items	100%	*n* = 1216	13-17 years old	Total ICU: *M* = 26.28, *SD* = 8.08;	---	---	---	---
Veroude et	Clinic	ICU - self-	0-4	Total: 24	56.4%	CD: *n* =	*Mean*	CD	56.4%	*n* =	*Mean* =	Total ICU: *M* = 19.47, *SD*

al (2016)<br>Netherlands		report		items		40<br><br>ADHD*: n* = 101<br><br>SIBS*: n* = 84	= 17.70 years old	Total ICU: *M* = 25.80, *SD* = 8.58;<br>ADHD<br>Total ICU: *M* = 23.25, *SD* = 6.71;<br>SIBS<br>Total ICU: *M* =20.65, *SD* = 5.73		103	17.19 years old	= 6.63
Viding et al. (2012)<br>USA	Community (with and without DBD)	ICU - self-report	0-3	Total: 24 items;	100%	*n* = 30	10-16 years old	CP/low CU<br>Total ICU: *M* = 34.73, *SD* = 8.16;<br>CP/ high CU<br>ICU Total: *M* = 53.47, *SD* = 5.50	100%	*n* = 166	10-16 years	Total ICU: *M* = 24.56, *SD* = 5.50
Wade et al. (2022)<br>Romania	Community	ICU - self-report	0-3	Total: 22 items	48.5%	*n* = 187	12-16 years old	Total ICU: *M* = 22.26, *SD* = 11.01	---	---	---	---
Wagner et al. (2015)<br>USA	Community	ICU - parent version	0-3	Total: 24 items	0%	*n* = 1080	48 months	48 months<br>Total ICU: *M* = 14.88, *SD* = 8.16;	---	---	---	---
Walker et al. (2020)<br>USA	Community	ICU - self-report	0-3	Total: 24 items	100%	*n* = 1216	13-17 years old	Total ICU: *M* = 26.19, *SD* = 8.05	---	---	---	---
Wallace et al. (2014)<br>USA	Community (with and without DBD)	ICU - parent version	0-3	Total: 24 items	100%	DBD: *n* = 22	10-18 years old	Total ICU: *M* = 28.59, *SD* = 10.10	100%	*n* = 27	10-18 years	Total ICU: *M* = 14.85, *SD* = 8.20
Waller et al. (2016)<br>USA	Community	ICU-parent version	0-3	Total: 24 items	100%	Primary caregiver: *n* = 533<br>Alternative caregiver: *n* = 392	*Mean* = 9.5 years old	Primary caregiver<br>Total ICU: *M* = 18.33, *SD* = 8.83;<br>Alternative caregiver<br>Total ICU: *M* = 19.41, *SD* = 8.61	---	---	---	---
Walsh et al. (2019)<br>USA	Forensic	ICU - self-report	0-3	Total: 24 items	100%	*n* = 1216	13-17 years old	Total ICU: *M* =25.17, *SD* = 8.46	---	---	---	---
Wang &	Community	ICU -	0-3	Total: 11	51.6%	*n* = 2523	*Mean*	Total ICU: *M* = 16.94, *SD*	---	---	---	---

Zhou (2023) China	y	teacher version		items			= 13.22 years old	= 4.40				
Waschbusch et al. (2019) USA	Clinical	ICU - self-report	0-3	Total: 24 items	78.3%	*n* = 46	*Mean* = 7-12.6 years old	Total ICU: *M* = 48.30, *SD* = 9.78	---	---	---	---
Waschbusch et al. (2024a) USA	Community	ICU - parent and teacher version	0-3	Total: 24 items	80.1%	*n* = 196	5-12 years old	Parent report Total ICU: *M* = 26.31, *SD* = 11.87; Teacher report Total ICU: *M* = 32.63, *SD* = 13.53	---	---	---	---
Waschbusch et al. (2024b) USA	Community	ICU - parent version	0-3	Total: 24 items	64%	*n* = 508	5-12 years old	Total ICU: *M* = 23.94, *SD* = 11.73	---	---	---	---
Waxmonsky et al. (2022) USA	Clinical	ICU - parent version	0-3	Total: 24 items	68.8 %	*n* = 48	5-12 years old	Total ICU: *M* = 29.06, *SD* = 11.29	---	---	---	---
Werhahn et al. (2021) USA	Community	ICU - self-report	0-3	Total: 24 items;	72.5%	DBD: *n* = 118	8-18 years old	Total ICU: *M* = 33.68, *SD* = 10.16; Callous: *M* = 12.00, *SD* = 6.11; Uncaring: *M* = 17.00, *SD* = 3.93; Unemotional: *M* = 7.17, *SD* = 3.31	72.5%	*n* = 89	8-18 years old	Total ICU: *M* = 21.00, *SD* = 8.70 Callous: *M* = 4.00, *SD* = 3.44 Uncaring: *M* = 10.41, *SD* = 5.07 Unemotional: *M* = 5.22, *SD* = 2.75
White et al. (2013) USA	Community	ICU - self-report	0-3	Total: 24 items	78.0%	DBD: *n* = 32	*Mean* = 14.38-14.90 years old	Total ICU: *M* = 47.78, *SD* = 13.08	59.0%	*n*= 27	*Mean* = 14.38-14.90 years	Total ICU: *M* = 17.88, *SD* = 10.05
White et al. (2013a) USA	Clinical	ICU-self-report	0-3	Total: 24 items	71.6%	*n* = 134	11-17 years old	Total ICU: *M* = 41.64, *SD* = 10.11	---	---	---	---
White et al. (2014) USA	Community (with and without DBD)	ICU - parent version	0-3	Total: 24 items	73.3%	DBD: *n* = 15	10-17 years old	Total ICU: *M* = 37.71, *SD* = 11.28	66.7%	*n*= 15	10-17 years old	Total ICU: *M* =14.53, *SD* = 6.52
White et al. (2016)	Clinical	ICU - self-report	0-3	Total: 24 items	72.2%	DBD: *n* = 72	*Mity*= 13.81	Total ICU: *M* =29.18, *SD* = 14.26	---	---	---	---

USA							years old					
Wilke & Goagoses (2023) Germany	Community	ICU - self-report	0-3	Total: 24 items; Callous: 11 items; Unemotional: 5 items; Uncaring: 8 items	58.8%	*n* = 194	6-11 years old	Callous: *M* = 18.26, *SD* = 4.95; Uncaring: *M* = 14.32, *SD* = 4.00 Unemotional: *M* = 10.35, *SD* = 3.00	---	---	---	---
Wilkinson & Jones (2021) UK	Community	ICU - teacher version	0-3	Total: 24 items	61.1%	*n* = 108	6-11 years old	ICU Total: *M* = 26.50, *SD* = 12.57	---	---	---	---
Winebrake et al. (2021) USA	Community	ICU – parent report	0-3	Total: 24 items	52.2%	*n* = 206	84 months	ICU Total: *M* = 16.80, *SD* = 7.92	---	---	---	---
Winstanley et al. (2021) UK	Forensic	ICU - self-report	0-3	Total: 24 items	77.0%	*n* = 87; with DLD: *n* = 58	*Mean* = 15.6-15.8 years old	Total ICU: *M* = 25.40, *SD* = 8.80; With DLD Total ICU: *M* = 30.80, *SD* = 9.00	---	---	---	---
Winters et al. (2023) USA	Community	ICU - self-report	0-3	Total: 24 items;	51.0%	*n* = 87	12-14 years old	ICU Total: *M* = 29.92, *SD* = 6.44	---	---	---	---
Winters et al. (2025) USA	Community (with and without DBD)	ICU - self-report	0-3	Total: 24 items;	53%	CP: *n* = 36	*Mean* = 13.05 years old	ICU Total: *M* = 15.17, *SD* = 6.06	53%	*n* = 49	*Mean* = 13.02 years old	ICU Total: *M* = 16.93, *SD* = 7.49
Winters & Sakai (2023) USA	Community	ICU - self-report	0-3	Total: 24 items	48.2%	*n* = 81	12-14 years old	ICU Total: *M* = 30.23, *SD* = 6.68	---	---	---	---
Wright et al. (2019) USA	Community	ICU - self-report	0-3	Total: 24 items; Callous: 11 items; Unemotional: 5 items;	50.8%	*n* = 1047	11-17 years old	Callous: *M* = 20.79, *SD* = 8.91; Unemotional: *M* = 7.50, *SD* = 3.50; Uncaring: *M* = 13.76, *SD* = 5.68	---	---	---	---

				Uncaring: 8 items								
Xie et al. (2020) China	Community	ICU - self-report	0-3	Total: 24 items Callous: 11 items; Unemotional: 5 items; Uncaring: 8 items	100%	*n* = 585	12-18 years old	Callous: *M* = 11.41, *SD* = 4.92; Uncaring: *M* = 8.76, *SD* = 3.84; Unemotional: *M* = 7.44, *SD* = 2.44	---	---	---	---
Xu et al. (2022a) China	Community	ICU - parent version	0-3	Total: 11 items	58.6%	*n* = 1216	11-16 years old	Total ICU: *M* = 20.55, *SD* = 6.12	---	---	---	---
Xu et al. (2022b) China	Community	ICU - parent version	0-3	Total: 11 items	52.0%	*n* = 441	8-11 years old	Total ICU: *M* = 20.79, *SD* = 4.51	---	---	---	---
Yang et al. (2021) China	Community	ICU - self-report	1-4	Total: 24 items	49.5%	*n* = 2407	11-16 years old	ICU Total: *M* = 49.68, *SD* = 8.64	---	---	---	---
Yang et al. (2024) China	Forensic	ICU - self-report	1-4	Total: 24 items	NA	*n* = 625	14-18 years old	ICU Total: *M* = 50.01, *SD* = 8.32	---	---	---	---
Zhang et al. (2021a) China	Community (with and without DBD)	ICU - parent version	0-3	Total: 24 items; Callous: 10 items; Uncaring: 9 items; Unemotional: 5 items	86.5%	*n* = 89	4-5 years	ADHD Total ICU: *M* = 29.70, *SD* = 10.4; Callous: *M* = 8.67, *SD* = 5.29; Uncaring: *M* = 15.87, *SD* = 4.03; Unemotional: *M* = 4.83, *SD* = 2.45; ADHD+ODD Total ICU: *M* = 32.04, *SD* = 8.30 Callous: *M* = 10.82, *SD* = 4.83; Uncaring: *M* = 16.82, *SD* = 3.75 Unemotional: *M* = 4.18, *SD* = 2.39	77.0%	*n* = 87	4-5 years	Total ICU: *M* = 21.39, *SD* = 7.40; Callous: *M* = 5.45, *SD* = 3.58; Uncaring: *M* = 10.79, *SD* = 4.07; Unemotional: *M* = 4.23, *SD* = 2.34
Zhang et al. (2021b) China	Community	ICU - parent version	0-3	Total: 11 items; Callous: 6 items; Uncaring: 5 items	46.1%	*n* = 382	7-11 years old	Boys Total ICU: *M* = 21.20, *SD* = 4.56; Uncaring: *M* = 10.01, *SD* = 2.08; Callous: *M* = 11.20, *SD* =	---	---	---	---

								3.02;<br>Girls<br>Total ICU: *M* = 19.67, *SD* = 4.37;<br>Uncaring: *M* = 9.42, *SD* = 2.25;<br>Callous: *M* = 10.25, *SD* = 2.92				
Zhong et al. (2020) China	Community	ICU - self-report and parent version	0-3	Total: 11 items;<br>Callous: 6 items;<br>Uncaring: 5 items	48.0%	*n* = 460	8-11 years old	Parents<br>Total ICU: *M* = 20.57, *SD* = 4.71;<br>Uncaring: *M* = 9.66, *SD* = 2.29;<br>Callous: *M* = 12.93, *SD* = 3.46;<br>Self-report<br>Total ICU: *M* = 17.75, *SD* = 5.00;<br>Uncaring: *M* = 8.43, *SD* = 2.61;<br>Callous: *M* = 9.32, *SD* = 3.31	---	---	---	---
Zumbach et al. (2021) Germany	Community	ICU - self-report	0-3	Total: 19 items;<br>Callous: 11 items;<br>Uncaring: 8 items	50.4%	*n* = 371	*Mean* = 72.44 months	Callous: *M* = 2.32, *SD* = 2.85;<br>Uncaring: *M* = 6.02, *SD* = 3.25	---	---	---	---

Note. ADHD = Attention defict/hyperactivity disorder; ASD = Autism Spectrum Disorder; CD = conduct disorder; CD+CU = conduct disorder with callous-unemotional traits; CP = conduct problems; CU = callous-unemotional traits; DBD = disruptive behavior disorder; ICU = Inventory of Callous-Unemotional Traits; ODD = opposite defiant disorder; SD = standard deviation; SIBS = siblings of probands
