# Supplementary material for: Reference Values of the Inventory of Callous-Unemotional Traits in Youth Populations: A Systematic Literature Review
Source: Clin Child Psychol Psychiatry. 2026 Mar 9;31(4):1927–50. doi: 10.1177/13591045261430966 (PMC13620238; doi:10.1177/13591045261430966)
Supplement: Supplementary file 3 [file sj-pdf-3-ccp-10.1177_13591045261430966.pdf]

References

Ackermann, K., Martinelli, A., Bernhard, A., Freitag, C. M., Büttner, G., & Schwenck, C. (2019). Friendship quality in youth with and without disruptive behavior disorders: The role of empathy, aggression, and callousness. *Child Psychiatry & Human Development*, *50*, 776-788. https://doi.org/10.1007/s10578-019-00880-x

Aggensteiner, P.-M., Böttinger, B., Baumeister, S., Hohmann, S., Heintz, S., Kaiser, A., Häge, A., Werhahn, J., Hofstetter, C., Walitza, S., Franke, B., Buitelaar, J., Banaschewski, T., Brandeis, D., & Holz, N. (2024). Randomized controlled trial of individualized arousal-biofeedback for children and adolescents with disruptive behavior disorders (DBD). *European Child & Adolescent Psychiatry, 33*, 3055-3066. https://doi.org/10.1007/s00787-023-02368-5

Aggensteiner, P.-M., Holz, N. E., Kaiser, A., Pernt, P. M., Böttinger, B., Baumeister, S., Werhahn, J., Walitza, S., Banaschewski, T., & Brandeis, D. (2022). Exploring psychophysiological indices of disruptive behavior disorders and their subtypes of aggression. *International Journal of Psychophysiology, 175*, 24–31. https://doi.org/10.1016/j.ijpsycho.2021.12.010

Allen, J. L., Morris, A., & Chhoa, C. Y. (2016). Callous–unemotional (CU) traits in adolescent boys and response to teacher reward and discipline strategies. *Emotional and Behavioural Difficulties*, *21*(3), 329-342. https://doi.org/10.1080/13632752.2016.1165968

Andershed, H., Colins, O. F., Salekin, R. T., Lordos, A., Kyranides, M. N., & Fanti, K. A. (2018). Callous-unemotional traits only versus the multidimensional psychopathy construct as predictors of various antisocial outcomes during early adolescence. *Journal of Psychopathology and Behavioral Assessment*, *40*, 16-25. https://doi.org/10.1007/s10862-018-9659-5

Anderson, S. A. S., Hawes, D. J., & Snow, P. C. (2024). Cognitive and affective perspective taking amongst adolescent offenders with variants of callous–unemotional traits. *European Child & Adolescent Psychiatry*, *33*(8), 2755–2765. https://doi.org/10.1007/s00787-023-02356-9

Ansel, L. L., Barry, C. T., Gillen, C. T., & Herrington, L. L. (2015). An analysis of four self-report measures of adolescent callous-unemotional traits: Exploring unique prediction of delinquency, aggression, and conduct problems. *Journal of Psychopathology and Behavioral Assessment*, *37*, 207-216. https://doi.org/10.1007/s10862-014-9460-z

Azzam, A. Y., Seleem, M. A., Saada, S. A., Mourad, H. A., & Mubarak, A. A. (2022). Serum oxytocin levels in adolescents with conduct disorder associated with callous-unemotional traits. *Middle East Current Psychiatry*, *29*(1), 52. https://doi.org/10.1186/s43045-022-00218-9

Bakker-Huvenaars, M. J., Greven, C. U., Herpers, P., Wiegers, E., Jansen, A., van der Steen, R., van Herwaarden, A. E., Baanders, A. N., Nijhof, K. S., Scheepers, F., Rommelse, N., Glennon, J. C., & Buitelaar, J. K. (2020). Saliva oxytocin, cortisol, and testosterone levels in adolescent boys with autism spectrum disorder, oppositional defiant disorder/conduct disorder and typically developing individuals. *European Neuropsychopharmacology*, *30*, 87–101. https://doi.org/10.1016/j.euroneuro.2018.07.097

Bansal, P. S., Babinski, D. E., Waxmonsky, J. G., & Waschbusch, D. A. (2022). Psychometric properties of parent ratings on the inventory of callous–unemotional traits in a nationally representative sample of 5-to 12-year-olds. *Assessment*, *29*(2), 242-256. https://doi.org/10.1177/1073191120964562

Bansal, P. S., Goh, P. K., Lee, C. A., & Martel, M. M. (2020). Conceptualizing callous-unemotional traits in preschool through confirmatory factor and network analysis. *Journal of Abnormal Child Psychology*, *48*, 539-550. https://doi.org/10.1007/s10802-019-00611-9

Barhight, L. R., Hubbard, J. A., Grassetti, S. N., & Morrow, M. T. (2017a). Relations between actual group norms, perceived peer behavior, and bystander children's intervention to bullying. *Journal of Clinical Child & Adolescent Psychology*, *46*(3), 394-400. https://doi.org/10.1080/15374416.2015.1046180

Barhight, L. R., Hubbard, J. A., Swift, L. E., & Konold, T. R. (2017b). A multitrait–multimethod approach to assessing childhood aggression and related constructs. *Merrill-Palmer Quarterly*, *63*(3), 367-395. https://doi.org/10.13110/merrpalmquar1982.63.3.0367

Baroncelli, A., Roti, B., & Ciucci, E. (2018). The associations between callous-unemotional traits and emotional awareness in youth. *Personality and Individual Differences*, *120*, 247-252. https://doi.org/10.1016/j.paid.2017.07.036

Barroso, R., Ramião, E., Figueiredo, P., & Araújo, A. M. (2021). Abusive sexting in adolescence: Prevalence and characteristics of abusers and victims. *Frontiers in Psychology*, *12*, 610474. https://doi.org/10.3389/fpsyg.2021.610474

Bell, G. R., Crothers, L. M., Hughes, T. L., Kanyongo, G. Y., Kolbert, J. B., & Parys, K. (2018). Callous-unemotional traits, relational and social aggression, and interpersonal maturity in a sample of behaviorally disordered adolescents. *Journal of Applied School Psychology*, *34*(1), 65-85. https://doi.org/10.1080/15377903.2017.1345814

Benesch, C., Görtz-Dorten, A., Breuer, D., & Döpfner, M. (2014). Assessment of callous-unemotional traits in 6 to 12 year-old children with oppositional defiant

disorder/conduct disorder by parent ratings. *Journal of Psychopathology and Behavioral Assessment*, *36*, 519-529. https://doi.org/10.1007/s10862-014-9420-7

Bennett, D. C., & Kerig, P. K. (2014). Investigating the construct of trauma-related acquired callousness among delinquent youth: Differences in emotion processing. *Journal of Traumatic Stress*, *27*(4), 415-422. https://doi.org/10.1002/jts.21931

Berg, J. M., Lilienfeld, S. O., Reddy, S. D., Latzman, R. D., Roose, A., Craighead, L. W., Pace, T. W. W., & Raison, C. L. (2013). The Inventory of Callous and Unemotional Traits: A Construct-Validational Analysis in an At-Risk Sample. *Assessment*, *20*(5), 532-544. https://doi.org/10.1177/1073191112474338

Bhanwer, A. K., Viljoen, J. L., Shaffer, C. S., & Douglas, K. S. (2019). The Inventory of Callous-Unemotional Traits: Reliability, Convergent Validity, and Predictive Validity for Reoffending in Adolescents on Probation. *International Journal of Forensic Mental Health*, *18*(2), 111-123. https://doi.org/10.1080/14999013.2018.1525779

Bird, E., Chhoa, C. Y., Midouhas, E., & Allen, J. L. (2019). Callous-unemotional traits and academic performance in secondary school students: Examining the moderating effect of gender. *Journal of Abnormal Child Psychology*, *47*, 1639-1650. https://doi.org/10.1007/s10802-019-00545-2

Bisby, M. A., Kimonis, E. R., & Goulter, N. (2017). Low maternal warmth mediates the relationship between emotional neglect and callous-unemotional traits among male juvenile offenders. *Journal of Child and Family Studies*, *26*, 1790-1798. https://doi.org/10.1007/s10826-017-0719-3

Blair, R. J. R., Bashford-Largo, J., Zhang, R., Lukoff, J., Elowsky, J. S., Leibenluft, E., Hwang, S., Dobbertin, M., & Blair, K. S. (2020). Temporal discounting impulsivity and its association with conduct disorder and irritability. *Journal of*

Child and Adolescent Psychopharmacology, 30(9), 542–548. https://doi.org/10.1089/cap.2020.0001

Blair, R. J., Zhang, R. U., Bashford-Largo, J., Bajaj, S., Mathur, A., Ringle, J., Schwartz, A., Elowsky, J., Dobbertin, M., Blair, K. S., & Tyler, P. M. (2021). Reduced neural responsiveness to looming stimuli is associated with increased aggression. *Social Cognitive and Affective Neuroscience*, *16*(10), 1091-1099. https://doi.org/10.1093/scan/nsab058

Blanchard, A., & Centifanti, L. C. M. (2017). Callous-unemotional traits moderate the relation between prenatal testosterone (2D: 4D) and externalising behaviours in children. *Child Psychiatry & Human Development*, *48*, 668-677. https://doi.org/10.1007/s10578-016-0690-z

Bonham, M. D., Hawkins, E., Waters, A. M., & Shanley, D. C. (2022). Can't stop, won't stop? The role of inhibitory control and callous-unemotional traits in childhood conduct problems and aggression. *Developmental Neuropsychology*, *47*(4), 210-225. https://doi.org/10.1080/87565641.2022.2069770

Böttinger, B. W., Aggensteiner, P.-M., Hohmann, S., Heintz, S., Ruf, M., Glennon, J., Holz, N. E., Banaschewski, T., Brandeis, D., & Baumeister, S. (2024). Exploring real-time functional magnetic resonance imaging neurofeedback in adolescents with disruptive behavior disorder and callous unemotional traits. *Journal of Affective Disorders, 345*, 32–42. https://doi.org/10.1016/j.jad.2023.10.036

Breaux, R., Babinski, D. E., Willoughby, M. T., Haas, S. M., Coles, E. K., Pelham, W. E., Waxmonsky, J. G., & Waschbusch, D. A. (2020). Examining psychopathic traits in children using the Child Psychopathy Scale–Revised. *Journal of Abnormal Child Psychology*, *48*, 251-263. https://doi.org/10.1007/s10802-019-00591-w

Cao, X., Somerville, M. P., Shou, Y., Xue, Z., & Allen, J. L. (2024). Callous-unemotional traits and child response to teacher rewards, discipline, and instructional methods in Chinese preschools: A classroom observation study. *Research on Child and Adolescent Psychopathology, 52*(3), 339–352. https://doi.org/10.1007/s10802-023-01137-x

Cardinale, E. M., Breeden, A. L., Robertson, E. L., Lozier, L. M., Vanmeter, J. W., & Marsh, A. A. (2018). Externalizing behavior severity in youths with callous–unemotional traits corresponds to patterns of amygdala activity and connectivity during judgments of causing fear. *Development and Psychopathology*, *30*(1), 191-201. https://doi.org/10.1017/S0954579417000566

Cardinale, E. M., O'Connell, K., Robertson, E. L., Meena, L. B., Breeden, A. L., Lozier, L. M., VanMeter, J. W., & Marsh, A. A. (2019). Callous and uncaring traits are associated with reductions in amygdala volume among youths with varying levels of conduct problems. *Psychological Medicine*, *49*(9), 1449-1458. https://doi.org/10.1017/S0033291718001927

Carvalho, M., Faria, M., Conceição, A., Matos, M., & Essau, C. (2018). Callous unemotional traits in children and adolescents. Psychometric properties of the portuguese version of the inventory of callous-unemotional traits. *European Journal of Psychological Assessment*, *34(*2), 101–110. https://doi.org/10.1027/1015-5759/a000449

Castagna, P. J., Babinski, D. E., Pearl, A. M., Waxmonsky, J. G., & Waschbusch, D. A. (2021a). Initial investigation of the psychometric properties of the limited prosocial emotions questionnaire (LPEQ). *Assessment*, *28*(8), 1882-1896. https://doi.org/10.1177/1073191120927782

Castagna, P. J., Babinski, D. E., & Waschbusch, D. A. (2025). Callous-unemotional traits moderate the association between inhibitory control and disruptive behavior problems. *European Child & Adolescent Psychiatry*, *34*, 1545–1555. https://doi.org/10.1007/s00787-024-02582-9

Castagna, P. J., & Waschbusch, D. A. (2021b). The importance of assessing impairment associated with limited prosocial emotions. Behavior Therapy, 52(5), 1237-1250. https://doi.org/10.1016/j.beth.2021.02.002

Cecil, C. A., McCrory, E. J., Barker, E. D., Guiney, J., & Viding, E. (2018). Characterising youth with callous–unemotional traits and concurrent anxiety: Evidence for a high-risk clinical group. *European Child & Adolescent Psychiatry*, *27*, 885-898. https://doi.org/10.1007/s00787-017-1086-8

Chang, S., Hou, Q., Wang, C., Wang, M., Wang, L., & Zhang, W. (2021). Childhood maltreatment and violent delinquency in Chinese juvenile offenders: Callous-unemotional traits as a mediator. *Child Abuse & Neglect*, *117*, 105085. https://doi.org/10.1016/j.chiabu.2021.105085

Charles, N. E., Acheson, A., Mathias, C. W., Michael Furr, R., & Dougherty, D. M. (2012). Psychopathic traits and their association with adjustment problems in girls. *Behavioral Sciences & the Law*, *30*(5), 631-642. https://doi.org/10.1002/bsl.2029

Chow, C. C., Nolte, T., Cohen, D., Fearon, R. M., & Shmueli-Goetz, Y. (2017). Reflective functioning and adolescent psychological adaptation: The validity of the Reflective Functioning Scale–Adolescent Version. *Psychoanalytic Psychology*, *34*(4), 404. https://doi.org/10.1037/pap0000148

Ciucci, E., & Baroncelli, A. (2014). Emotion-related personality traits and peer social standing: Unique and interactive effects in cyberbullying behaviors.

Cyberpsychology, Behavior, and Social Networking, *17*(9), 584-590. https://doi.org/10.1089/cyber.2014.0020

Ciucci, E., Baroncelli, A., Facci, C., Righi, S., & Frick, P. J. (2024). Callous–unemotional traits and emotion perception accuracy and bias in youths. *Children*, *11*(4), 419. https://doi.org/10.3390/children11040419

Ciucci, E., Baroncelli, A., Franchi, M., Golmaryami, F. N., & Frick, P. J. (2014). The association between callous-unemotional traits and behavioral and academic adjustment in children: Further validation of the Inventory of Callous-Unemotional Traits. *Journal of Psychopathology and Behavioral Assessment*, *36*, 189-200. https://doi.org/10.1007/s10862-013-9384-z

Ciucci, E., Kimonis, E., Frick, P. J., Righi, S., Baroncelli, A., Tambasco, G., & Facci, C. (2018). Attentional orienting to emotional faces moderates the association between callous-unemotional traits and peer-nominated aggression in young adolescent school children. *Journal of Abnormal Child Psychology*, *46*, 1011-1019. https://doi.org/10.1007/s10802-7-0357-7

Clark, J. E., & Frick, P. J. (2018). Positive parenting and callous-unemotional traits: Their association with school behavior problems in young children. *Journal of Clinical Child & Adolescent Psychology*, *47*(sup1), S242-S254. https://doi.org/10.1080/15374416.2016.1253016

Coe, J. L., Davies, P. T., & Sturge-Apple, M. L. (2018). Family instability and young children's school adjustment: Callousness and negative internal representations as mediators. *Child Development*, *89*(4), 1193-1208. https://doi.org/10.1111/cdev.12793

Colins, O. F., Andershed, H., Hawes, S. W., Bijttebier, P., & Pardini, D. A. (2016). Psychometric properties of the original and short form of the Inventory of

Callous-Unemotional Traits in detained female adolescents. *Child Psychiatry & Human Development*, *47*, 679-690. https://doi.org/10.1007/s10578-015-0601-8

Colins, O. F., Andershed, H., Salekin, R. T., & Fanti, K. A. (2018). Comparing different approaches for subtyping children with conduct problems: Callous-unemotional traits only versus the multidimensional psychopathy construct. *Journal of Psychopathology and Behavioral Assessment*, *40*, 6-15. https://doi.org/10.1007/s10862-018-9653-y

Corbelli, G., Levantini, V., Muratori, P., Senese, V. P., Bravaccio, C., Pisano, S., Catone, G., & Paciello, M. (2024). Assessing callous-unemotional traits across early adolescence: Further evaluation of short versions. *Child Psychiatry & Human Development*. Advance online publication. https://doi.org/10.1007/s10578-024-01746-7

Craig, M. C., Mulder, L. M., Zwiers, M. P., Sethi, A., Hoekstra, P. J., Dietrich, A., Baumeister, S., Aggensteiner, P. M., Banaschewski, T., Brandeis, D., Werhahn, J. E., Walitza, S., Castro-Fornieles, J., Arango, C., Schulze, U. M. E., Glennon, J. C., Franke, B., Santosh, P. J., Mastroianni, M., van Asten, J. J. A., & Naaijen, J. (2019). Distinct associations between fronto-striatal glutamate concentrations and callous-unemotional traits and proactive aggression in disruptive behavior. *Cortex*, *121*, 135-146. https://doi.org/10.1016/j.cortex.2019.08.017

Craig, S. G., & Moretti, M. M. (2019). Profiles of primary and secondary callous-unemotional features in youth: The role of emotion regulation. *Development and psychopathology*, *31*(4), 1489-1500. https://doi.org/10.1017/S0954579418001062

Dandreaux, D. M., & Frick, P. J. (2009). Developmental pathways to conduct problems: A further test of the childhood and adolescent-onset distinction.

Journal of Abnormal Child Psychology, *37*, 375-385. https://doi.org/10.1007/s10802-008-9261-5

Dapprich, A. L., Derks, L. M., Holtmann, M., Lange, W.-G., Legenbauer, T., & Becker, E. S. (2024). Hostile and threatening interpretation biases in adolescent inpatients are specific to callous-unemotional traits and social anxiety. *European Child & Adolescent Psychiatry*, *33*, 1143–1150. https://doi.org/10.1007/s00787-023-02227-3

Dapprich, A. L., Tyborowska, A., Niermann, H. C., Becker, E. S., Cillessen, A. H., & Roelofs, K. (2022). Behavioral inhibition as an early life predictor of callous-unemotional traits. *International Journal of Behavioral Development*, *46*(4), 333-345. https://doi.org/10.1177/01650254221100245

Dargis, M., & Li, J. J. (2020). The interplay between positive and negative parenting and children's negative affect on callous-unemotional traits. *Journal of Child and Family Studies*, *29*(9), 2614-2622. https://doi.org/10.1007/s10826-020-01783-5

Davis, T., Ammons, C., Dahl, A., & Kliewer, W. (2015). Community violence exposure and callous–unemotional traits in adolescents: Testing parental support as a promotive versus protective factor. *Personality and Individual Differences*, *77*, 7-12. https://doi.org/10.1016/j.paid.2014.12.024

De Brito, S. A., Rogers, J. C., Pauli, R., Kohls, G., Raschle, N. M., Martinelli, A., Smaragdi, A., Gonzalez-Madruga, K., Cornwell, H., Stadler, C., Konrad, K., Freitag, C. M., & Fairchild, G. (2025). Brain Responses During Face Processing in Conduct Disorder: Considering Sex and Callous-Unemotional Traits. *Biological Psychiatry*. https://doi.org/10.1016/j.biopsych.2025.04.023

DeLisi, M., Vaughn, M., Beaver, K. M., Wexler, J., Barth, A. E., & Fletcher, J. M. (2011). Fledgling psychopathy in the classroom: ADHD subtypes, psychopathy,

and reading comprehension in a community sample of adolescents. *Youth violence and Juvenile Justice*, *9*(1), 43-58. https://doi.org/10.1177/1541204010371932

Derks, L. M., Becker, E. S., Lange, W.-G., Rinck, M., Dapprich, A. L., Holtmann, M., & Legenbauer, T. (2024a). The relationship between callous-unemotional traits and internalizing psychopathology in adolescent psychiatric inpatients: a network analysis. *Child and Adolescent Psychiatry and Mental Health*, *18*(1). https://doi.org/10.1186/s13034-024-00853-6

Derks, L. M., Becker, E. S., Rinck, M., Holtmann, M., Legenbauer, T., & Lange, W.-G. (2024b). I can't feel your face: callous-unemotional traits, social anxiety, and approach-avoidance behaviour in conduct disorder. *Child and Adolescent Psychiatry and Mental Health, 18*(1). https://doi.org/10.1186/s13034-024-00831-y

Docherty, M., Boxer, P., Huesmann, L. R., O'Brien, M., & Bushman, B. J. (2016). Exploring primary and secondary variants of psychopathy in adolescents in detention and in the community. *Journal of Clinical Child & Adolescent Psychology*, *45*(5), 564-578. https://doi.org/10.1080/15374416.2014.979934

Dotterer, H. L., Tomlinson, R. C., Burt, S. A., Weigard, A. S., Klump, K. L., & Hyde, L. W. (2021). Neurocognitive abilities associated with antisocial behavior with and without callous-unemotional traits in a community sample. *Neuropsychology*, *35*(4), 374. https://doi.org/10.1037/neu0000733

Drent, H. M., van den Hoofdakker, B., de Bildt, A., Buitelaar, J. K., Hoekstra, P. J., & Dietrich, A. (2020). Factors related to parental pre-treatment motivation in outpatient child and adolescent mental health care. *European Child & Adolescent Psychiatry*, *29*(7), 947-958. https://doi.org/10.1007/s00787-019-01391-9

Dugré, J. R., & Potvin, S. (2023). Functional Connectivity of the Nucleus Accumbens across Variants of Callous-Unemotional Traits: A Resting-State fMRI Study in Children and Adolescents. *Research on Child and Adolescent Psychopathology, 52*(3), 353–368. https://doi.org/10.1007/s10802-023-01143-z

Eisenbarth, H., Demetriou, C. A., Kyranides, M. N., & Fanti, K. A. (2016). Stability subtypes of callous–unemotional traits and conduct disorder symptoms and their correlates. *Journal of Youth and Adolescence*, *45*, 1889-1901. https://doi.org/10.1007/s10964-016-0520-4

Elizur, Y., Somech, L. Y., & Vinokur, A. D. (2017). Effects of parent training on callous-unemotional traits, effortful control, and conduct problems: Mediation by parenting. *Journal of Abnormal Child Psychology*, *45*, 15-26. https://doi.org/10.1007/s10802-016-0163-7

Elowsky, J., Bajaj, S., Bashford-Largo, J., Zhang, R., Mathur, A., Schwartz, A., Dobbertin, M., Blair, K. S., Leibenluft, E., Pardini, D., & Blair, R. J. R. (2022). Differential associations of conduct disorder, callous-unemotional traits and irritability with outcome expectations and values regarding the consequences of aggression. *Child and Adolescent Psychiatry and Mental Health*, *16*(1), 38. https://doi.org/10.1186/s13034-022-00466-x

Essau, C. A., Sasagawa, S., & Frick, P. J. (2006). Callous-unemotional traits in a community sample of adolescents. *Assessment*, *13*(4), 454-469. https://doi.org/10.1177/1073191106287354

Euler, F., Sterzer, P., & Stadler, C. (2014). Cognitive control under distressing emotional stimulation in adolescents with conduct disorder. *Aggressive Behavior*, *40*(2), 109-119. https://doi.org/10.1002/ab.21508

Everett, V. S., & Drabick, D. A. (2023). Community Violence Exposure and Generalized Anxiety Symptoms: Do Callous-Unemotional Behaviors Moderate this Relation Among Urban Youth?. *Research on Child and Adolescent Psychopathology, 51*(1), 87-102. https://doi.org/10.1007/s10802-022-00973-7

Ezpeleta, L., Granero, R., De La Osa, N., & Domènech, J. M. (2015). Clinical characteristics of preschool children with oppositional defiant disorder and callous-unemotional traits. *PloS one*, *10*(9), e0139346. https://doi.org/10.1371/journal.pone.0139346

Ezpeleta, L., Granero, R., de la Osa, N., & Domènech, J. M. (2017). Developmental trajectories of callous-unemotional traits, anxiety and oppositionality in 3–7 year-old children in the general population. *Personality and Individual Differences*, *111*, 124-133. http://dx.doi.org/10.1016/j.paid.2017.02.005

Ezpeleta, L., Osa, N. D. L., Granero, R., Penelo, E., & Domènech, J. M. (2013). Inventory of callous-unemotional traits in a community sample of preschoolers. *Journal of Clinical Child & Adolescent Psychology*, *42*(1), 91-105. https://doi.org/10.1080/15374416.2012.734221

Ezpeleta, L., Penelo, E., de la Osa, N., Navarro, J. B., Fañanás, L., & Fatjó-Vilas, M. (2019). Association of OXTR rs53576 with the developmental trajectories of callous-unemotional traits and stressful life events in 3-to 9-year-old community children. *Journal of Abnormal Child Psychology*, *47*(10), 1651-1662. https://doi.org/10.1007/s10802-019-00548-z

Facci, C., Baroncelli, A., Frick, P. J., & Ciucci, E. (2024). Dimensions of Parenting and Children's Conduct Problems: The Importance of Considering Children's Callous–Unemotional Traits. *International Journal of Environmental Research and Public Health, 21*(6), 779. https://doi.org/10.3390/ijerph21060779

Fagan, S. E., Zhang, W., & Gao, Y. (2017). Social adversity and antisocial behavior: Mediating effects of autonomic nervous system activity. *Journal of Abnormal Child Psychology, 45*, 1553-1564. https://doi.org/10.1007/s10802-017-0262-0

Fairchild, G., Hagan, C. C., Passamonti, L., Walsh, N. D., Goodyer, I. M., & Calder, A. J. (2014). Atypical neural responses during face processing in female adolescents with conduct disorder. *Journal of the American Academy of Child & Adolescent Psychiatry, 53*(6), 677-687. https://doi.org/10.1016/j.jaac.2014.02.009

Fang, J., Wang, W., Gao, L., Yang, J., Wang, X., Wang, P., & Wen, Z. (2022). Childhood maltreatment and adolescent cyberbullying perpetration: a moderated mediation model of callous—unemotional traits and perceived social support. Journal of interpersonal violence, 37(7-8), NP5026-NP5049. https://doi.org/10.1177/0886260520960106

Fang, J., Wang, X., Yuan, K. H., & Wen, Z. (2020). Childhood psychological maltreatment and moral disengagement: A moderated mediation model of callous-unemotional traits and empathy. *Personality and Individual Differences*, *157*, 109814. https://doi.org/10.1016/j.paid.2020.109814

Fanniff, A. M., & Kimonis, E. R. (2014). Juveniles who have committed sexual offenses: A special group?. *Behavioral Sciences & the Law, 32*(2), 240-257. https://doi.org/10.1002/bsl.2111

Fanti, K. A., & Kimonis, E. R. (2012). Bullying and victimization: The role of conduct problems and psychopathic traits. *Journal of Research on Adolescence*, *22*(4), 617-631. https://doi.org/10.1111/j.1532-7795.2012.00809.x

Fanti, K. A., & Kimonis, E. R. (2013). Dimensions of juvenile psychopathy distinguish “bullies,”“bully-victims,” and “victims”. *Psychology of Violence*, *3*(4), 396. https://doi.org/10.1037/a0033951

Fanti, Kostas., Hadjicharalambous, M.-Z., & Katsimicha, E. (2013a). Adolescent Callous-Unemotional Traits Mediates the Longitudinal Association between Conduct Problems and Media Violence Exposure. *Societies*, *3*(3), 298–315. https://doi.org/10.3390/soc3030298

Fanti, K. A., Colins, O. F., Andershed, H., & Sikki, M. (2017). Stability and change in callous-unemotional traits: Longitudinal associations with potential individual and contextual risk and protective factors. *American Journal of Orthopsychiatry*, *87*(1), 62. https://doi.org/ 10.1037/ort0000143

Fanti, K. A., Demetriou, C. A., & Kimonis, E. R. (2013b). Variants of callous-unemotional conduct problems in a community sample of adolescents. *Journal of Youth and Adolescence*, *42*, 964-979. https://doi.org/10.1007/s10964-013-9958-9

Fanti, K. A., Frick, P. J., & Georgiou, S. (2009). Linking callous-unemotional traits to instrumental and non-instrumental forms of aggression. *Journal of Psychopathology and Behavioral Assessment*, *31*, 285-298. https://doi.org/10.1007/s10862-008-9111-3

Fanti, K. A., Kokkinos, C. M., Voulgaridou, I., & Hadjicharalambous, M. Z. (2019). Investigating the association between callous-unemotional traits with relational bullying and victimization: A cross-national study. *Social Development*, *28*(4), 854-872. https://doi.org/10.1111/sode.12381

Fanti, K. A., Kyranides, M. N., Lordos, A., Colins, O. F., & Andershed, H. (2018). Unique and interactive associations of callous-unemotional traits, impulsivity and grandiosity with child and adolescent conduct disorder symptoms. *Journal of Psychopathology and Behavioral Assessment*, *40*, 40-49. https://doi.org/10.1007/s10862-018-9655-9

Feilhauer, J., Cima, M., & Arntz, A. (2012). Assessing callous–unemotional traits across different groups of youths: Further cross-cultural validation of the Inventory of Callous–Unemotional Traits. *International Journal of Law and Psychiatry, 35*(4), 251-262. https://doi.org/10.1016/j.ijlp.2012.04.002

Feilhauer, J., Cima, M., Benjamins, C., & Muris, P. (2013a). Knowing right from wrong, but just not always feeling it: relations among callous-unemotional traits, psychopathological symptoms, and cognitive and affective morality judgments in 8-to 12-year-old boys. *Child Psychiatry & Human Development*, *44*, 709-716. https://doi.org/10.1007/s10578-013-0363-0

Feilhauer, J., Cima, M., Korebrits, A., & Nicolson, N. A. (2013b). Salivary cortisol and psychopathy dimensions in detained antisocial adolescents. *Psychoneuroendocrinology*, *38*(9),1586-1595. https://doi.org/10.1016/j.psyneuen.2013.01.005

Figueiredo, P., Moreira, D., Ramião, E., Barroso, R., & Barbosa, F. (2022). Psychometric properties of the Portuguese teacher-version of the Inventory of Callous-Unemotional Traits. *Clinical Child Psychology and Psychiatry, 27*(3), 852 – 869. https://doi.org/10.1177/13591045211070168

Figueiredo, P., Ramião, E., Moreira, D., Barroso, R., & Barbosa, F. (2023). Psychometric properties of the Portuguese teacher-version of the Inventory of Callous-Unemotional Traits in Preschoolers. *Análise Psicológica, 41*(1), 127-142. https://doi.org/10.14417/ap.1991

Fine, A., Cavanagh, C., Frick, P. J., Steinberg, L., & Cauffman, E. (2017). Can probation officers identify remorse among male adolescent offenders?. *Psychological Assessment*, *29*(6), 754. https://dx.doi.org/10.1037/pas0000391

Fink, B. C., Tant, A. S., Tremba, K., & Kiehl, K. A. (2012). Assessment of psychopathic traits in an incarcerated adolescent sample: A methodological comparison. *Journal of Abnormal Child Psychology*, *40*(6), 971-986. https://doi.org/10.1007/s10802-012-9614-y

Fisher, J. H., & Brown, J. L. (2018). A prospective, longitudinal examination of the influence of childhood home and school contexts on psychopathic characteristics in adolescence. *Journal of Youth and Adolescence*, *47*(10), 2041-2059. https://doi.org/10.1007/s10964-018-0861-2

Fite, P. J., Griffith, R., Tampke, E. C., Stone, K. J., & Srivastava, V. (2020). Factors associated with perceived containment. *Journal of Community Psychology*, *48*(3), 932-944. https://doi.org/10.1002/jcop.22312

Fite, P. J., Pederson, C., & DiPierro, M. (2018). Individual risk factors associated with increased levels of restricted housing among detained youth. *Residential Treatment for Children & Youth*, *35*(2), 139-154. https://doi.org/10.1080/0886571X.2018.1467812

Fite, P. J., Williford, A., Griffith, R. L., & Parker, K. (2020). Peer victimization among detained youth: The impact of callous-unemotional traits. In *Child & Youth Care Forum* (Vol. 50, pp. 569-585). Springer US. https://doi.org/10.1007/s10566-020-09593-y

Fleming, G. E., Neo, B., Briggs, N. E., Kaouar, S., Frick, P. J., & Kimonis, E. R. (2022). Parent training adapted to the needs of children with callous–unemotional traits: A randomized controlled trial. *Behavior Therapy*, *53*(6), 1265-1281. https://doi.org/10.1016/j.beth.2022.07.001

Fragkaki, I., & Cima, M. (2019). The effect of oxytocin administration on empathy and emotion recognition in residential youth: A randomized, within-subjects trial.

Hormones and Behavior, *114*, 104561. https://doi.org/10.1016/j.yhbeh.2019.104561

Fragkaki, I., Cima, M., & Meesters, C. (2016). The association between callous–unemotional traits, externalizing problems, and gender in predicting cognitive and affective morality judgments in adolescence. *Journal of Youth and Adolescence*, *45*, 1917-1930. https://doi.org/10.1007/s10964-016-0527-x

Fragkaki, I., Verhagen, M., van Herwaarden, A. E., & Cima, M. (2019a). Daily oxytocin patterns in relation to psychopathy and childhood trauma in residential youth. *Psychoneuroendocrinology*, *102*,105-113. https://doi.org/10.1016/j.psyneuen.2018.11.040

Fragkaki, I., Weijman, E. L., & Cima, M. (2019b). Dissociation and psychopathology in residential youth: a brief report. *Journal of Trauma & Dissociation, 20*(5), 594-602. https://doi.org/10.1080/15299732.2019.1597816

Gao, Y., Huang, Y., & Li, X. (2017). Interaction between prenatal maternal stress and autonomic arousal in predicting conduct problems and psychopathic traits in children. *Journal of Psychopathology and Behavioral Assessment*, *39*(1), 1-14. https://doi.org/10.1007/s10862-016-9556-8

Gao, Y., & Raine, A. (2024). Primary and Secondary Variants of Callous-Unemotional Traits in Community Youths: Differences in Anticipatory Fear. *Children, 11*(3), 359. https://doi.org/10.3390/children11030359

Gao, Y., & Zhang, W. (2016). Confirmatory factor analyses of self-and parent-report inventory of callous-unemotional traits in 8-to 10-year-olds. *Journal of Psychopathology and Behavioral Assessment, 38*, 331-340. https://doi.org/10.1007/s10862-015-9527-5

Gao, Y., & Zhang, W. (2021). Reward processing and psychopathic traits in children. *Personality Disorders: Theory, Research, and Treatment*, *12*(4), 339. https://doi.org/10.1037/per0000430

Gaule, A., Bevilacqua, L., Molleman, L., van den Bos, W., van Duijvenvoorde, A. C., Roberts, R., Pease, C. R., McCrory, E., & Viding, E. (2024a). Social learning and preferences in adolescents with conduct problems and varying levels of callous-unemotional traits. *JAACAP Open*, *2*(2), 79–89. https://doi.org/10.1016/j.jaacop.2023.12.008

Gaule, A., Martin, P., Lockwood, P. L., Cutler, J., Apps, M., Roberts, R., Phillips, H., Brown, K., McCrory, E. J., & Viding, E. (2024b). Reduced prosocial motivation and effort in adolescents with conduct problems and callous-unemotional traits. *Journal of Child Psychology and Psychiatry*, *65*(8), 1061–1071. Portico. https://doi.org/10.1111/jcpp.13945

Georgiou, G., Demetriou, C. A., & Fanti, K. A. (2019). Distinct empathy profiles in callous unemotional and autistic traits: Investigating unique and interactive associations with affective and cognitive empathy. *Journal of Abnormal Child Psychology*, *47*(11), 1863-1873. https://doi.org/10.1007/s10802-019-00562-1

Gillen, C. T., MacDougall, E. A., Forth, A. E., Barry, C. T., & Salekin, R. T. (2019). Validity of the youth psychopathic traits inventory–short version in justice-involved and at-risk adolescents. *Assessment*, *26*(3), 479-491. https://doi.org/10.1177/1073191117700723

Gluckman, N. S., Hawes, D. J., & Russell, A. M. (2016). Are callous-unemotional traits associated with conflict adaptation in childhood?. *Child Psychiatry & Human Development*, *47*, 583-592. https://doi.org/10.1007/s10578-015-0593-4

Goagoses, N., & Koglin, U. (2021). Attachment and externalizing behavior: mediation through dysfunctional emotion regulation and callous-unemotional traits. *International Journal of Developmental Science*, *15*(1-2), 9-17. https://doi.org/10.3233/DEV-200291

Goagoses, N., Schipper, N., & Koglin, U. (2022). Callous-unemotional traits, social goal orientations, and bullying perpetration: exploring concurrent associations during adolescence. *International Journal of Adolescence and Youth*, *27*(1), 135-148. https://doi.org/10.1080/02673843.2022.2037441

Goffin, K. C., Boldt, L. J., Kim, S., & Kochanska, G. (2018). A unique path to callous-unemotional traits for children who are temperamentally fearless and unconcerned about transgressions: a longitudinal study of typically developing children from age 2 to 12. *Journal of Abnormal Child Psychology*, *46*, 769-780. https://doi.org/10.1007/s10802-017-0317-2

Gong, J., Wang, M. C., Zhang, X., Zeng, H., & Yang, W. (2022). The Emotion Regulation Questionnaire for Children and Adolescents (ERQ-CA): Factor structure and measurement invariance in a Chinese student samples. *Journal of Personality Assessment*, *104*(6), 774-783. https://doi.org/10.1080/00223891.2021.2014506

González-Madruga, K., Rogers, J., Toschi, N., Riccelli, R., Smaragdi, A., Puzzo, I., Roberta, C., Andersson, J., Baumann, S., Kohls, G., Raschle, N., Fehlbaum, L., Menks, W., Stadler, C., Konrad, K., Freitag, C. M., De Brito, S. A., Sonuga-Barke, E., & Fairchild, G. (2020). White matter microstructure of the extended limbic system in male and female youth with conduct disorder. *Psychological Medicine*, *50*(1), 58-67. https://doi.org/10.1017/S0033291718003951

Grassetti, S. N., Tennity, C. L., Charles, N. E., Turner, E. A., & Barry, C. T. (2023). Parenting practices and callous unemotional traits predict behavioral infractions at military-style youth challenge academies. *Current Psychology*, *42*(32), 28517-28526. https://doi.org/10.1007/s12144-022-03840-2

Graziano, P. A., Garic, D., & Dick, A. S. (2022a). Individual differences in white matter of the uncinate fasciculus and inferior fronto-occipital fasciculus: Possible early biomarkers for callous-unemotional behaviors in young children with disruptive behavior problems. *Journal of Child Psychology and Psychiatry*, *63*(1), 19-33. https://doi.org/10.1111/jcpp.13444

Graziano, P. A., Landis, T., Maharaj, A., Ros-Demarize, R., Hart, K. C., & Garcia, A. (2022b). Differentiating preschool children with conduct problems and callous-unemotional behaviors through emotion regulation and executive functioning. *Journal of Clinical Child & Adolescent Psychology*, *51*(2), 170-182. https://doi.org/10.1080/15374416.2019.1666399

Grotzinger, A. D., Mann, F. D., Patterson, M. W., Tackett, J. L., Tucker-Drob, E. M., & Harden, K. P. (2018). Hair and salivary testosterone, hair cortisol, and externalizing behaviors in adolescents. *Psychological Science*, *29*(5), 688-699. https://doi.org/10.1177/0956797617742981

Guelker, M. D., Barry, C. T., Barry, T. D., & Malkin, M. L. (2014). Perceived positive outcomes as a mediator between adolescent callous-unemotional traits and antisocial behavior. *Personality and Individual Differences*, *69*, 129-134. https://doi.org/10.1016/j.paid.2014.05.029

Harenski, C. L., Harenski, K. A., & Kiehl, K. A. (2014). Neural processing of moral violations among incarcerated adolescents with psychopathic traits.

Developmental Cognitive Neuroscience, *10*, 181-189. https://doi.org/10.1016/j.dcn.2014.09.002

Hawes, S. W., Byrd, A. L., Henderson, C. E., Gazda, R. L., Burke, J. D., Loeber, R., & Pardini, D. A. (2014). Refining the parent-reported Inventory of Callous–Unemotional Traits in boys with conduct problems. *Psychological Assessment, 26*(1), 256–266. https://doi.org/10.1037/a0034718

He, J., Gao, Y., Deng, J., & Wang, M.-C. (2023). Resting heart rate moderates the relationship between parental emotion socialization and callous-unemotional traits in children. *European Child & Adolescent Psychiatry*, *33*(7), 2353–2363. https://doi.org/10.1007/s00787-023-02338-x

Herpers, P. C., Bakker-Huvenaars, M. J., Greven, C. U., Wiegers, E. C., Nijhof, K. S., Baanders, A. N., Buitelaar, J. K., & Rommelse, N. N. J. (2019). Emotional valence detection in adolescents with oppositional defiant disorder/conduct disorder or autism spectrum disorder. *European Child & Adolescent Psychiatry*, *28*, 1011-1022. https://doi.org/10.1007/s00787-019-01282-z

Herpers, P. C., Klip, H., Rommelse, N. N., Greven, C. U., & Buitelaar, J. K. (2016). Associations between high callous–unemotional traits and quality of life across youths with non-conduct disorder diagnoses. *European Child & Adolescent Psychiatry*, *25*(5), 547-555. https://doi.org/10.1007/s00787-015-0766-5

Heynen, E., van der Helm, P., Cima, M., Stams, G. J., & Korebrits, A. (2017). The relation between living group climate, aggression, and callous-unemotional traits in delinquent boys in detention. *International Journal of Offender Therapy and Comparative Criminology*, *61*(15), 1701-1718. https://doi.org/10.1177/0306624X16630543

Hitti, S. A., McDonald, S. E., Green, K. E., Bouchard, L. M., & Sullivan, T. N. (2021). Evaluation of the self-report Inventory of Callous-Unemotional Traits in a sample of urban middle school students: A multidimensional Rasch analysis. *Journal of Psychopathology and Behavioral Assessment*, *43*, 332-342. https://doi.org/10.1007/s10862-020-09811-w

Hodsoll, S., Lavie, N., & Viding, E. (2014). Emotional attentional capture in children with conduct problems: The role of callous-unemotional traits. *Frontiers in Human Neuroscience*, *8,* 570. https://doi.org/10.3389/fnhum.2014.00570

Hostetler, N., Tavares, T. P., Ritchie, M. B., Oliver, L. D., Chen, V. V., Greening, S., Finger, E. C., & Mitchell, D. G. V. (2024). Prefrontal cortex structural and developmental associations with callous-unemotional traits and aggression. *Scientific Reports, 14*(1). https://doi.org/10.1038/s41598-024-54481-3

Howard, A. L., Kimonis, E. R., Muñoz, L. C., & Frick, P. J. (2012). Violence exposure mediates the relation between callous-unemotional traits and offending patterns in adolescents. Journal of Abnormal Child Psychology, 40, 1237-1247. https://doi.org/10.1007/s10802-012-9647-2

Hoyniak, C. P., Bates, J. E., Petersen, I. T., Yang, C. L., Darcy, I., & Fontaine, N. M. (2018). Reduced neural responses to vocal fear: a potential biomarker for callous-uncaring traits in early childhood. Developmental Science, 21(4), e12608. https://doi.org/10.1111/desc.12608

Hulvershorn, L. A., Finn, P., Hummer, T. A., Leibenluft, E., Ball, B., Gichina, V., & Anand, A. (2013). Cortical activation deficits during facial emotion processing in youth at high risk for the development of substance use disorders. *Drug and Alcohol Dependence, 131*(3), 230-237. https://doi.org/10.1016/j.drugalcdep.2013.05.015

Hwang, S., Allen, J. L., Kokosi, T., & Bird, E. (2021). To what extent does punishment insensitivity explain the relationship between callous-unemotional traits and academic performance in secondary school students?. *British Journal of Educational Psychology*, *91*(3), 811-826. https://doi.org/10.1111/bjep.12394

Hwang, S., Nolan, Z. T., White, S. F., Williams, W. C., Sinclair, S., & Blair, R. J. R. (2016). Dual neurocircuitry dysfunctions in disruptive behavior disorders: emotional responding and response inhibition. *Psychological Medicine*, *46*(7), 1485-1496. https://doi.org/10.1017/S0033291716000118

Johander, E., Trach, J., Turunen, T., Garandeau, C. F., & Salmivalli, C. (2022). Intention to stop bullying following a condemning, empathy-raising, or combined message from a teacher–do students' empathy and callous-unemotional traits matter?. *Journal of Youth and Adolescence*, *51*(8), 1568-1580. https://doi.org/10.1007/s10964-022-01613-5

Johnson, M. M., Dismukes, A. R., Vitacco, M. J., Breiman, C., Fleury, D., & Shirtcliff, E. A. (2014). Psychopathy's influence on the coupling between hypothalamic–pituitary–adrenal and-gonadal axes among incarcerated adolescents. *Developmental Psychobiology*, *56*(3), 448-458. https://doi.org/10.1002/dev.21111

Kahn, R. E., Deater-Deckard, K., King-Casas, B., & Kim-Spoon, J. (2016). Intergenerational similarity in callous-unemotional traits: Contributions of hostile parenting and household chaos during adolescence. *Psychiatry Research*, *246*, 815-820. https://doi.org/10.1016/j.psychres.2016.10.023

Kahn, R. E., Frick, P. J., Golmaryami, F. N., & Marsee, M. A. (2017). The moderating role of anxiety in the associations of callous-unemotional traits with self-report and laboratory measures of affective and cognitive empathy. *Journal of Abnormal Child Psychology*, *45*, 583-596. https://doi.org/10.1007/s10802-016-0179-z

Kaouar, S., Fleming, G. E., Neo, B., Hawes, D. J., Eapen, V., & Kimonis, E. R. (2023). Dimensions of Warm Parenting Attributions Differentiate Conduct Problem Subtypes in Young Children. *Research on Child and Adolescent Psychopathology, 52*(2), 223–236. https://doi.org/10.1007/s10802-023-01111-7

Kauten, R. L., Lui, J. H., Doucette, H., & Barry, C. T. (2015). Perceived family conflict moderates the relations of adolescent narcissism and CU traits with aggression. *Journal of Child and Family Studies*, *24*, 2914-2922. https://doi.org/10.1007/s10826-014-0095-1

Kavish, N., Vaughn, M. G., Cho, E., Barth, A., Boutwell, B., Vaughn, S., Capin, P., Stillman, S., & Martinez, L. (2017). Physiological arousal and juvenile psychopathy: Is low resting heart rate associated with affective dimensions?. *Psychiatric Quarterly*, *88*, 103-114. https://doi.org/10.1007/s11126-016-9437-z

Kemp, E. C., Frick, P. J., Matlasz, T. M., Clark, J. E., Robertson, E. L., Ray, J. V., Thornton, L. C., Wall Myers, T. D., Steinverg, L., & Cauffman, E. (2023). Developing cutoff scores for the inventory of callous-unemotional traits (ICU) in justice-involved and community samples. *Journal of Clinical Child & Adolescent Psychology*, *52*(4), 519-532. https://doi.org/10.1080/15374416.2021.1955371

Kemp, E. C., Ray, J. V., Frick, P. J., Robertson, E. L., Fanti, K. A., Essau, C. A., Baroncelli, A., Ciucci, E., & Bijttebier, P. (2024). Inventory of Callous-Unemotional Traits (ICU) factor structure and measurement invariance in an adolescent multinational sample. *Journal of Clinical Child & Adolescent Psychology*, 1-12. https://doi.org/10.1080/15374416.2022.2148531

Kemp, E. C., Frick, P. J., Matlasz, T. M., Clark, J. E., Robertson, E. L., Ray, J. V., Thornton, L. C., Myers, T. D. W., Steinberg, L., & Cauffman, E. (2022). Establishing cut-off scores for the Inventory of Callous-Unemotional Traits

(ICU). *Journal of Clinical Child and Adolescent Psychology*, 1–14. https://doi.org/10.1080/15374416.2021.1955371

Kerig, P. K., Bennett, D. C., Thompson, M., & Becker, S. P. (2012). “Nothing really matters”: Emotional numbing as a link between trauma exposure and callousness in delinquent youth. *Journal of Traumatic Stress*, *25*(3), 272-279. https://doi.org/10.1002/jts.21700

Kerig, P. K., & Modrowski, C. A. (2020). Testing gender-differentiated models of the mechanisms linking polyvictimization and youth offending: Numbing and callousness versus dissociation and borderline traits. *Journal of Trauma & Dissociation*, *19*, 347-361. https://doi.org/10.1080/15299732.2018.1441355

Kilshaw, R. E., Kerig, P. K., & Baucom, B. R. W. (2022). Vocally-encoded emotional arousal as a marker of callous-unemotional traits in a sample of justice-involved adolescents. *Child Psychiatry & Human Development, 55*(5), 1238–1249. https://doi.org/10.1007/s10578-022-01486-6

Kimonis, E. R., Cooper, F., Neo, B., Fleming, G. E., Chan, M. E., McDonogh, C., & Dit Bressel, P. J.-R. (2025). Affective and behavioral responses to time-out in preschool children with conduct problems and varying levels of callous-unemotional traits. *Behavior Therapy, 56*(2), 422–437. https://doi.org/10.1016/j.beth.2024.07.005

Kimonis, E. R., Fanti, K. A., Isoma, Z., & Donoghue, K. (2013). Maltreatment profiles among incarcerated boys with callous-unemotional traits. *Child Maltreatment*, *18*(2), 108-121. https://doi.org/10.1177/1077559513483002

Kimonis, E. R., Fleming, G. E., Wilbur, R. R., Groer, M. W., & Granger, D. A. (2019a). Dehydroepiandrosterone (DHEA) and its ratio to cortisol moderate associations between maltreatment and psychopathology in male juvenile offenders. *Psychoneuroendocrinology*, *101*, 263-271. https://doi.org/10.1016/j.psyneuen.2018.12.228

Kimonis, E. R., Fleming, G., Briggs, N., Brouwer-French, L., Frick, P. J., Hawes, D. J., Bagner, D. M., Thomas, R., & Dadds, M. (2019b). Parent-child interaction therapy adapted for preschoolers with callous-unemotional traits: An open trial pilot study. *Journal of Clinical Child & Adolescent Psychology*, *48*(sup1), S347-S361. https://doi.org/10.1080/15374416.2018.1479966

Kimonis, E. R., Frick, P. J., Munoz, L. C., & Aucoin, K. J. (2007). Can a laboratory measure of emotional processing enhance the statistical prediction of aggression and delinquency in detained adolescents with callous-unemotional traits?. *Journal of Abnormal Child Psychology*, *35*, 773-785. https://doi.org/10.1007/s10802-007-9136-1

Kimonis, E. R., Goulter, N., Hawes, D. J., Wilbur, R. R., & Groer, M. W. (2017). Neuroendocrine factors distinguish juvenile psychopathy variants. *Developmental Psychobiology*, *59*(2), 161-173. https://doi.org/10.1002/dev.21473

Kimonis, E. R., Graham, N., & Cauffman, E. (2018). Aggressive male juvenile offenders with callous-unemotional traits show aberrant attentional orienting to distress cues. *Journal of Abnormal Child Psychology*, *46*, 519-527. https://doi.org/10.1007/s10802-017-0295-4

Kimonis, E. R., Kennealy, P. J., & Goulter, N. (2016). Does the self-report inventory of callous-unemotional traits predict recidivism?. *Psychological Assessment*, *28*(12), 1616. https://doi.org/10.1037/pas0000292

Kimonis, E. R., Le, B., Fleming, G. E., Kyranides, M. N., Demetriou, C. A., Fanti, K. A., Neo, B., Prasad, A. H., Chan, A., Hawes, D. J., & Eapen, V. (2023). Facial reactions to emotional films in young children with conduct problems and varying levels of callous-unemotional traits. *Journal of Child Psychology and Psychiatry*, *64*(3), 357-366. https://doi.org/10.1111/jcpp.13701

Kimonis, E. R., Ray, J. V., Branch, J. R., & Cauffman, E. (2011). Anger mediates the relation between violence exposure and violence perpetration in incarcerated boys. In *Child & Youth Care Forum* (Vol. 40, pp. 381-400). Springer US. https://doi.org/10.1007/s10566-010-9121-7

Kleberg, J. L., Frick, M. A., & Brocki, K. C. (2021). Increased pupil dilation to happy faces in children with hyperactive/impulsive symptoms of ADHD. *Development and Psychopathology*, *33*(3), 767-777. https://doi.org/10.1017/S0954579420000036

Kochanska, G., Kim, S., Boldt, L. J., & Yoon, J. E. (2013). Children's callous-unemotional traits moderate links between their positive relationships with parents at preschool age and externalizing behavior problems at early school age. *Journal of Child Psychology and Psychiatry*, *54*(11), 1251-1260. https://doi.org/10.1111/jcpp.12084

Kofler, L., Zhang, W., & Gao, Y. (2022). Psychopathic Traits and Conduct Problems in Children: Effects of Collective Efficacy, Heart Rate, and Sex. *Journal of Psychopathology and Behavioral Assessment*, *44*(1), 98-114. https://doi.org/10.1007/s10862-021-09944-6

Koh, L., Neo, B., Fleming, G. E., Kaouar, S., Henery, J., Briggs, N., & Kimonis, E. R. (2024). Development and validation of the Parental Affection/Warmth Scale (PAWS) in a sample of parents of 2- to 8-year-olds. *Psychological Assessment, 36*(12), 722–734. https://doi.org/10.1037/pas0001343

Kongerslev, M. T., Bo, S., Forth, A. E., & Simonsen, E. (2015). Assessment of the affective dimensions of psychopathy with the Danish version of the Inventory of Callous-Unemotional Traits among incarcerated boys: A study of reliability, criterion validity, and construct validity. *Scandinavian Journal of Child and*

Adolescent Psychiatry and Psychology, *3*(1), 80-96. https://doi.org/10.21307/sjcapp-2015-008

Kumsta, R., Sonuga-Barke, E., & Rutter, M. (2012). Adolescent callous–unemotional traits and conduct disorder in adoptees exposed to severe early deprivation. *The British Journal of Psychiatry*, *200*(3), 197-201. https://doi.org/10.1192/bjp.bp.110.089441

Kurek, A., Jose, P. E., & Stuart, J. (2019). 'I did it for the LULZ': How the dark personality predicts online disinhibition and aggressive online behavior in adolescence. *Computers in Human Behavior*, *98*, 31-40. https://doi.org/10.1016/j.chb.2019.03.027

Kyranides, M. N., Fanti, K. A., Katsimicha, E., & Georgiou, G. (2018). Preventing conduct disorder and callous unemotional traits: preliminary results of a school based pilot training program. *Journal of Abnormal Child Psychology*, *46*, 291-303. https://doi.org/10.1007/s10802-017-0273-x

Lam, B. Y. H., Huang, Y., & Gao, Y. (2021). Gray matter asymmetry in the orbitofrontal cortex concerning psychopathic traits in adolescents. *Journal of Psychiatric Research*, *132*, 84-96. https://doi.org/10.1016/j.jpsychires.2020.10.003

Latzman, R. D., Malikina, M. V., Hecht, L. K., Lilienfeld, S. O., & Chan, W. Y. (2016). The contribution of personality and refugee camp experience to callous and unemotional traits among immigrant adolescents in the United States: implications for the DSM-5 "Limited Prosocial Emotions" Specifier. *Child Psychiatry & Human Development*, *47*, 215-225. https://doi.org/10.1007/s10578-015-0558-7

Lau, K. S., & Marsee, M. A. (2013). Exploring narcissism, psychopathy, and Machiavellianism in youth: Examination of associations with antisocial behavior and aggression. *Journal of Child and Family Studies*, *22*, 355-367. https://doi.org/10.1007/s10826-012-9586-0

Lee-Rowland, L. M., Lui, J. H., Bortfeld, D., Barry, C. T., & Reiter, S. (2020). Internalizing problems and their impact on the relation between callous-unemotional traits, narcissism, and aggression. *Aggressive Behavior, 46*(3), 278-286. https://doi.org/10.1002/ab.21888

Levy, T., Peskin, M., Kohn, Y., Sheinhorn, S., Schoen, G., Weizman, A., & Golubchik, P. (2022). Callous-unemotional traits and face-emotion recognition as mediators in conduct problems of children with ADHD. *Clinical Child Psychology and Psychiatry, 27*(4), 978-990. https://doi.org/10.1177/13591045221093876

Liu, W., & Guo, Z. (2024). Influence of campus exclusion on bullying behavior of junior high school students: role of callous-unemotional traits and family caring. *BMC Psychology, 12*(1). https://doi.org/10.1186/s40359-024-02124-7

López-Romero, L., Gómez-Fraguela, J. A., & Romero, E. (2015a). Assessing callous-unemotional traits in a Spanish sample of institutionalized youths: The Inventory of Callous-Unemotional Traits. *Journal of Psychopathology and Behavioral Assessment*, *37*, 392-406. https://doi.org/10.1007/s10862-014-9469-3

López-Romero, L., Romero, E., & Gómez-Fraguela, J. A. (2015b). Delving into callous–unemotional traits in a Spanish sample of adolescents: Concurrent correlates and early parenting precursors. *Journal of Child and Family Studies*, *24*, 1451-1468. https://doi.org/10.1007/s10826-014-9951-2

Lorber, C. M., Hughes, T. L., Miller, J. A., Crothers, L. M., & Martin, E. (2011). Callous and unemotional traits and social cognitive processes in a sample of

community-based aggressive youth. *International Journal of Offender Therapy and Comparative Criminology*, *55*(8), 1291-1307. https://doi.org/10.1177/0306624X11386012

Lozier, L. M., Cardinale, E. M., VanMeter, J. W., & Marsh, A. A. (2014). Mediation of the relationship between callous-unemotional traits and proactive aggression by amygdala response to fear among children with conduct problems. *JAMA Psychiatry*, *71*(6), 627-636. https://doi.org/10.1001/jamapsychiatry.2013.4540

Lui, J. H., Barry, C. T., & Sacco, D. F. (2016). Callous-unemotional traits and empathy deficits: Mediating effects of affective perspective-taking and facial emotion recognition. *Cognition and Emotion*, *30*(6), 1049-1062. https://doi.org/10.1080/02699931.2015.1047327

Lui, J. H., Barry, C. T., & Schoessler, M. (2017). The indirect effects of adolescent psychopathic traits on aggression through social-cognitive factors. *Journal of child and family studies*, *26*, 1298-1309. https://doi.org/10.1007/s10826-017-0667-y

Lynch, S. F., Perlstein, S., Ordway, C., Jones, C., Lembcke, H., Waller, R., & Wagner, N. J. (2024). Parasympathetic nervous system functioning moderates the associations between callous-unemotional traits and emotion understanding difficulties in late childhood. *Children, 11*(2), 184. https://doi.org/10.3390/children11020184

Ma, Y., Xing, X., & Zhang, M. (2022). Parental rejection and school-aged children’s externalizing behavior problems in China: The roles of executive function and callous-unemotional traits. *Child Psychiatry & Human Development, 55*(1), 152–163. https://doi.org/10.1007/s10578-022-01397-6

Maneiro, L., Gómez-Fraguela, J. A., López-Romero, L., Cutrín, O., & Sobral, J. (2019). Risk profiles for antisocial behavior in adolescents placed in residential care. *Children and Youth Services Review*, *103*, 278-286. https://doi.org/10.1016/j.childyouth.2019.06.012

Mann, F. D., Tackett, J. L., Tucker-Drob, E. M., & Harden, K. P. (2018). Callous-unemotional traits moderate genetic and environmental influences on rule-breaking and aggression: Evidence for gene× trait interaction. *Clinical Psychological Science*, *6*(1), 123-133. https://doi.org/10.1177/2167702617730889

Marsee, M. A., Frick, P. J., Barry, C. T., Kimonis, E. R., Centifanti, L. C. M., & Aucoin, K. J. (2014a). Profiles of the forms and functions of self-reported aggression in three adolescent samples. *Development and Psychopathology*, *26*(3), 705-720. https://doi.org/10.1017/S0954579414000339

Marsee, M. A., Lau, K. S., & Lapré, G. E. (2014b). Parent and adolescent report of the forms and functions of aggression: Associations with delinquency, CU traits, and dysregulation. In *Child & Youth Care Forum* (Vol. 43, pp. 27-39). Springer US. https://doi.org/10.1007/s10566-013-9223-0

Martin-Key, N. A., Allison, G., & Fairchild, G. (2020). Empathic accuracy in female adolescents with conduct disorder and sex differences in the relationship between conduct disorder and empathy. *Journal of Abnormal Child Psychology*, *48*, 1155-1167. https://doi.org/10.1007/s10802-020-00659-y

Martin-Key, N. A., Graf, E. W., Adams, W. J., & Fairchild, G. (2021). Investigating emotional body posture recognition in adolescents with conduct disorder using eye-tracking methods. *Research on Child and Adolescent Psychopathology*, *49*, 849-860. https://doi.org/10.1007/s10802-021-00784-2

Masi, G., Manfredi, A., Milone, A., Muratori, P., Polidori, L., Ruglioni, L., & Muratori, F. (2011). Predictors of nonresponse to psychosocial treatment in children and adolescents with disruptive behavior disorders. *Journal of Child and Adolescent Psychopharmacology*, *21*(1), 51-55. https://doi.org/10.1089/cap.2010.0039

Masi, G., Pisano, S., Brovedani, P., Maccaferri, G., Manfredi, A., Milone, A., Nocentini, A., Polidori, L., Ruglioni, L., & Muratori, P. (2018). Trajectories of callous–unemotional traits from childhood to adolescence in referred youth with a disruptive behavior disorder who received intensive multimodal therapy in childhood. *Neuropsychiatric Disease and Treatment*, 2287-2296. https://doi.org/10.2147/NDT.S164032

Matlasz, T. M., Frick, P. J., & Clark, J. E. (2020). Understanding the social relationships of youth with callous-unemotional traits using peer nominations. *Journal of Clinical Child & Adolescent Psychology*, *51*(4), 530-542. https://doi.org/10.1080/15374416.2020.1823847

Matlasz, T. M., Frick, P. J., & Clark, J. E. (2023). A comparison of parent, teacher, and youth ratings on the inventory of callous–unemotional traits. *Assessment*, *30*(1), 210-224. https://doi.org/10.1177/10731911211047893

Mattos, L. A., Schmidt, A. T., Henderson, C. E., & Hogue, A. (2017). Therapeutic alliance and treatment outcome in the outpatient treatment of urban adolescents: The role of callous–unemotional traits. *Psychotherapy*, *54*(2), 136–147. https://doi.org/10.1037/pst0000093

McLoughlin, N., Rucklidge, J. J., Grace, R. C., & McLean, A. P. (2010). Can callous-unemotional traits and aggression identify children at high-risk of anti-social

behavior in a low socioeconomic group?. *Journal of Family Violence*, *25*, 701-712. https://doi.org/10.1007/s10896-010-9320-x

Mendez, L., Mozley, M. M., & Kerig, P. K. (2020). Associations among trauma exposure, callous-unemotionality, race or ethnicity, and gang involvement in justice-involved youth. *Criminal Justice and Behavior*, *47*(4), 457-469. https://doi.org/10.1177/0093854819897940

Milledge, S. V., Cortese, S., Thompson, M., McEwan, F., Rolt, M., Meyer, B., Sonuga-Barke, E., & Eisenbarth, H. (2019). Peer relationships and prosocial behaviour differences across disruptive behaviours. *European Child & Adolescent Psychiatry*, *28*, 781-793. https://doi.org/10.1007/s00787-018-1249-2

Miller, M. A., & Marsee, M. A. (2019). Emotional reactivity and antisocial behavior relative to Posttraumatic stress symptom expression: A latent profile analysis. *Journal of Abnormal Child Psychology*, *47*, 1339-1350. https://doi.org/10.1007/s10802-019-00514-9

Miller, N. V., Haas, S. M., Waschbusch, D. A., Willoughby, M. T., Helseth, S. A., Crum, K. I., Coles, E. K., & Pelham, W. E. (2014). Behavior therapy and callous-unemotional traits: Effects of a pilot study examining modified behavioral contingencies on child behavior. *Behavior Therapy, 45*(5), 606–618. https://doi.org/10.1016/j.beth.2013.10.006

Mozley, M. M., Modrowski, C. A., & Kerig, P. K. (2018). The roles of trauma exposure, rejection sensitivity, and callous-unemotional traits in the aggressive behavior of justice-involved youth: A moderated mediation model. *Aggressive Behavior*, *44*(3), 268-275. https://doi.org/10.1002/ab.21749

Muñoz, L. C. (2009). Callous-unemotional traits are related to combined deficits in recognizing afraid faces and body poses. *Journal of the American Academy of*

Child & Adolescent Psychiatry, *48*(5), 554-562. https://doi.org/10.1097/CHI.0b013e31819c2419

Muñoz, L. C., Frick, P. J., Kimonis, E. R., & Aucoin, K. J. (2008a). Verbal ability and delinquency: Testing the moderating role of psychopathic traits. *Journal of Child Psychology and Psychiatry*, *49*(4), 414-421. https://doi.org/10.1111/j.1469-7610.2007.01847.x

Muñoz, L. C., Frick, P. J., Kimonis, E. R., & Aucoin, K. J. (2008b). Types of aggression, responsiveness to provocation, and callous-unemotional traits in detained adolescents. *Journal of Abnormal Child Psychology*, *36*, 15-28. https://doi.org/10.1007/s10802-007-9137-0

Muñoz, L. C., Qualter, P., & Padgett, G. (2011). Empathy and bullying: Exploring the influence of callous-unemotional traits. *Child Psychiatry & Human Development*, *42*, 183-196. https://doi.org/10.1007/s10578-010-0206-1

Muratori, P., Lochman, J. E., Lai, E., Milone, A., Nocentini, A., Pisano, S., Righini, E., & Masi, G. (2016). Which dimension of parenting predicts the change of callous unemotional traits in children with disruptive behavior disorder?. *Comprehensive Psychiatry*, *69*, 202-210. https://doi.org/10.1016/j.comppsych.2016.06.002

Muratori, P., Milone, A., Levantini, V., Ruglioni, L., Lambruschi, F., Pisano, S., Masi, G., & Lochman, J. E. (2019). Six-year outcome for children with ODD or CD treated with the coping power program. *Psychiatry Research*, *271*, 454-458. https://doi.org/10.1016/j.psychres.2018.12.018

Muratori, P., Paciello, M., Castro, E., Levantini, V., Masi, G., Milone, A., Senese, V. P., Pisano, S., & Catone, G. (2021). At-risk early adolescents profiles in the community: A cluster analysis using the strengths and difficulties questionnaire.

Psychiatry Research, *305*, 114209. https://doi.org/10.1016/j.psychres.2021.114209

Myers, T. D. W., Salcedo, A., Frick, P. J., Ray, J. V., Thornton, L. C., Steinberg, L., & Cauffman, E. (2018). Understanding the link between exposure to violence and aggression in justice-involved adolescents. *Development and Psychopathology*, *30*(2), 593-603. https://doi.org/10.1017/S0954579417001134

Nichols, S. R., Briggs-Gowan, M. J., Estabrook, R., Burns, J. L., Kestler, J., Berman, G., Henry, D. B., & Wakschlag, L. S. (2015). Punishment insensitivity in early childhood: A developmental, dimensional approach. *Journal of Abnormal Child Psychology*, *43*, 1011-1023. https://doi.org/10.1007/s10802-014-9950-1

Normand, S., Miller, N. V., & Mikami, A. Y. (2023). Contributions of friends' problem behaviors to friendship quality in a sample of children with ADHD. *Journal of Clinical Child & Adolescent Psychology*, *52*(2), 244-258. https://doi.org/10.1080/15374416.2021.1941056

Nwafor, C. E., Onyeizugbo, E. U., & Anazonwu, C. O. (2015). Does gender moderate the relationship between callous-unemotional traits and physical aggression? *The Spanish Journal of Psychology, 18*. https://doi.org/10.1017/sjp.2015.86

Paciello, M., Masi, G., Clemente, M. G., Milone, A., & Muratori, P. (2017). Moral disengagement and callous unemotional traits configurations in adolescents with disruptive behavior disorder: a person-oriented approach. *Psychiatry Research*, *258*, 591-593. https://doi.org/10.1016/j.psychres.2017.08.043

Payot, M., Monseur, C., & Stievenart, M. (2023). Primary and secondary variants of callous–unemotional traits in early and middle childhood: Distinction, evaluation and empathic differences. *Child Psychiatry & Human Development*, *56*(2), 543–555. https://doi.org/10.1007/s10578-023-01576-z

Pechorro, P., Braga, T., Hawes, S. W., Gonçalves, R. A., Simões, M. R., & Ray, J. V. (2019). The Portuguese version of the Inventory of Callous-Unemotional Traits self-report and its short form among a normative sample of community youths. *Child Psychiatry & Human Development, 50*, 245-256. https://doi.org/10.1007/s10578-018-0835-3

Pechorro, P., Goncalves, R. A., Hawes, S. W., & Ray, J. V. (2018). Psychometric properties of two short versions of the Inventory of Callous–Unemotional Traits among incarcerated youth. *Journal of Emotional and Behavioral Disorders, 26*(4), 243-256. https://doi.org/10.1177/1063426617717940

Pederson, C. A., Fite, P. J., Weigand, P. D., Myers, H., & Housman, L. (2020). Implementation of a behavioral intervention in a juvenile detention center: do individual characteristics matter?. *International Journal of Offender Therapy and Comparative Criminology*, *64*(1), 83-99. https://doi.org/10.1177/0306624X19872627

Perlstein, S., Waller, R., Wagner, N., Byrd, A., Vine, V., Jennings, J. R., & Stepp, S. (2021). Autonomic nervous system inflexibility during parent–child interactions is related to callous-unemotional traits in youth aged 10–14 years old. *Research on Child and Adolescent Psychopathology*, *49*, 1581-1592. https://doi.org/10.1007/s10802-021-00849-2

Pihet, S., Etter, S., Schmid, M., & Kimonis, E. R. (2015). Assessing callous-unemotional traits in adolescents: Validity of the inventory of callous-unemotional traits across gender, age, and community/institutionalized status. *Journal of Psychopathology and Behavioral Assessment*, *37*, 407-421. https://doi.org/10.1007/s10862-014-9472-8

Piscitello, J., Robertson, E. L., Renaud, M., Leon, D., Hayes, T., & Pelham, W. E. (2025). Impact of an 8-week, intensive summer treatment program on improving coercive caregiver-child interactions among children with and without callous unemotional traits. *Behavior Therapy, 56*(2), 276–289. https://doi.org/10.1016/j.beth.2024.08.008

Platje, E., Huijbregts, S. C., van Goozen, S. H., Popma, A., Cima, M., & Swaab, H. J. (2018). Executive functioning, reward/punishment sensitivity, and conduct problems in boys with callous-unemotional traits. *International Journal of Offender Therapy and Comparative Criminology, 62*(13), 4008-4023. https://doi.org/10.1177/0306624X18758897

Portengen, C. M., Sprooten, E., Zwiers, M. P., Hoekstra, P. J., Dietrich, A., Holz, N. E., Aggensteiner, P. M., Banaschewski, T., Schulze, U. M. E., Saam, M. C., Craig, M. C., Sethi, A., Santosh, P., Ouriaghli, I. S., Castro-Fornieles, J., Rosa, M., Arango, C., Penzol, M. J., Werhahn, J. E., Brandeis, D., Walitza, S., Oldehinkel, M., Franke, B., Buitelaar, J. K., & Naaijen, J. (2021). Reward and punishment sensitivity are associated with cross-disorder traits. *Psychiatry Research, 298*, 113795. https://doi.org/10.1016/j.psychres.2021.113795

Portnoy, J., Cui, N., Raine, A., Frazier, A., Rudo-Hutt, A. S., & Liu, J. (2020). Autonomic nervous system activity and callous-unemotional traits in physically maltreated youth. *Child Abuse & Neglect, 101*, 104308. https://doi.org/10.1016/j.chiabu.2019.104308

Powell, T., Plate, R. C., Miron, C. D., Wagner, N. J., & Waller, R. (2024). Callous-unemotional traits and emotion recognition difficulties: do stimulus characteristics play a role? *Child Psychiatry & Human Development, 55*(6), 1453–1462. https://doi.org/10.1007/s10578-023-01510-3

Preston, O. C., Gillen, C. T., Anestis, J. C., Charles, N. E., & Barry, C. T. (2021). The validity of the Personality Assessment Inventory—Adolescent in assessing callous-unemotional traits in at-risk adolescents. *Journal of Personality Assessment*, *103*(1), 48-56. https://doi.org/10.1080/00223891.2019.1705462

Priddis, L. E., Landy, S., Moroney, D., & Kane, R. (2014). An exploratory study of aggression in school-age children: Underlying factors and implications for treatment. *Journal of Psychologists and Counsellors in Schools*, *24*(1), 18-35. https://doi.org/10.1017/jgc.2013.12

Puzzo, I., Seunarine, K., Sully, K., Darekar, A., Clark, C., Sonuga-Barke, E. J., & Fairchild, G. (2018). Altered white-matter microstructure in conduct disorder is specifically associated with elevated callous-unemotional traits. *Journal of Abnormal Child Psychology*, *46*, 1451-1466. https://doi.org/10.1007/s10802-017-0375-5

Rehder, P. D., Mills-Koonce, W. R., Wagner, N. J., Zvara, B., & Willoughby, M. T. (2021). Attachment quality assessed from children's family drawings links to child conduct problems and callous-unemotional behaviors. *Attachment & Human Development*, *23*(3), 239-256. https://doi.org/10.1080/14616734.2020.1714676

Rehder, P. D., Mills-Koonce, W. R., Willoughby, M. T., Garrett-Peters, P., Wagner, N. J., & Family Life Project Key Investigators. (2017). Emotion recognition deficits among children with conduct problems and callous-unemotional behaviors. *Early Childhood Research Quarterly*, *41*, 174-183. https://doi.org/10.1016/j.ecresq.2017.07.007

Rhoads, S. A., Cardinale, E. M., O'Connell, K., Palmer, A. L., VanMeter, J. W., & Marsh, A. A. (2020). Mapping neural activity patterns to contextualized fearful facial expressions onto callous-unemotional (CU) traits: intersubject

representational similarity analysis reveals less variation among high-CU adolescents. *Personality Neuroscience, 3*. https://doi.org/10.1017/pen.2020.13

Rizeq, J., Toplak, M. E., Ledochowski, J., Basile, A., & Andrade, B. F. (2020). Callous-unemotional traits and executive functions are unique correlates of disruptive behavior in children. *Developmental Neuropsychology*, *45*(3), 154-166. https://doi.org/10.1080/87565641.2020.1737698

Roberts, R., McCrory, E., Bird, G., Sharp, M., Roberts, L., & Viding, E. (2020). Thinking about others' minds: mental state inference in boys with conduct problems and callous-unemotional traits. *Journal of Abnormal Child Psychology*, *48*, 1279-1290. https://doi.org/10.1007/s10802-020-00664-1

Roberts, R., McCrory, E., Joffe, H., De Lima, N., & Viding, E. (2018). Living with conduct problem youth: family functioning and parental perceptions of their child. *European Child & Adolescent Psychiatry*, *27*, 595-604. https://doi.org/10.1007/s00787-017-1088-6

Robertson, E. L., Frick, P. J., Ray, J. V., Thornton, L. C., Wall Myers, T. D., Steinberg, L., & Cauffman, E. (2021). Do callous–unemotional traits moderate the effects of the juvenile justice system on later offending behavior? *Journal of Child Psychology and Psychiatry, 62*(2), 212-222. https://doi.org/10.1111/jcpp.13266

Romero, E., & Alonso, C. (2017). Callous-unemotional traits and the five factor model in adolescents. *Personality and Individual Differences, 106*, 268-274. https://doi.org/10.1016/j.paid.2016.10.056

Roose, A., Bijttebier, P., Van der Oord, S., Claes, L., & Lilienfeld, S. O. (2013). Psychopathic traits in youth and associations with temperamental features. *Journal of Individual Differences*. https://doi.org/10.1027/1614-0001/a000090

Sanchez, C., & Cooley, J. (2024). Peer victimization and callous-unemotional traits: the impact of parents and teachers. *Research on Child and Adolescent Psychopathology, 52*(10), 1551–1564. https://doi.org/10.1007/s10802-024-01213-w

Scavenius, C., Granski, M., Lindberg, M. R., Vardanian, M. M., & Chacko, A. (2020). Adolescent gender and age differences in responsiveness to functional family therapy. *Family Process*, *59*(4), 1465-1482. https://doi.org/10.1111/famp.12512

Schwenck, C., Ciaramidaro, A., Selivanova, M., Tournay, J., Freitag, C. M., & Siniatchkin, M. (2017). Neural correlates of affective empathy and reinforcement learning in boys with conduct problems: fMRI evidence from a gambling task. *Behavioural Brain Research*, *320*, 75-84. https://doi.org/10.1016/j.bbr.2016.11.037

Sebastian, C. L., De Brito, S. A., McCrory, E. J., Hyde, Z. H., Lockwood, P. L., Cecil, C. A., & Viding, E. (2016). Grey matter volumes in children with conduct problems and varying levels of callous-unemotional traits. *Journal of Abnormal Child Psychology*, *44*, 639-649. https://doi.org/10.1007/s10802-015-0073-0

Sebastian, C. L., McCrory, E. J., Dadds, M. R., Cecil, C. A. M., Lockwood, P. L., Hyde, Z. H., De Brito, S. A., & Viding, E. (2014). Neural responses to fearful eyes in children with conduct problems and varying levels of callous–unemotional traits. *Psychological Medicine*, *44*(1), 99-109. https://doi.org/10.1017/S0033291713000482

Sharf, A., Kimonis, E. R., & Howard, A. (2014). Negative life events and posttraumatic stress disorder among incarcerated boys with callous-unemotional traits. *Journal of Psychopathology and Behavioral Assessment*, *36*, 401-414. https://doi.org/10.1007/s10862-013-9404-z

Sharp, C., & Vanwoerden, S. (2014). Social cognition: Empirical contribution: The developmental building blocks of psychopathic traits: Revisiting the role of theory of mind. *Journal of Personality Disorders, 28*(1), 78-95. https://doi.org/10.1521/pedi.2014.28.1.78

Simpson, T. P., Frick, P. J., Kahn, R. E., & Evans, L. J. (2013). Therapeutic alliance in justice-involved adolescents undergoing mental health treatment: The role of callous-unemotional traits. *International Journal of Forensic Mental Health*, *12*(2), 83-92. https://doi.org/10.1080/14999013.2013.787559

Sourander, A., McGrath, P. J., Ristkari, T., Cunningham, C., Huttunen, J., Lingley-Pottie, P., Hinkka-YliSalomaki, S., Kinnunen, M., Vuorio, J., Sinokki, A., Fossum, S., & Unruh, A. (2016). Internet-assisted parent training intervention for disruptive behavior in 4-year-old children: a randomized clinical trial. *JAMA Psychiatry*, *73*(4), 378-387. https://doi.org/10.1001/jamapsychiatry.2015.3411

Speck, J. S., Frick, P. J., Vaughan, E. P., Walker, T. M., Robertson, E. L., Ray, J. V., Myers, T. D. W., Thornton, L. C., Steinberg, L., & Cauffman, E. (2024). Health service utilization in adolescents following a first arrest: the role of antisocial behavior, callous-unemotional traits, and juvenile justice system processing. *Administration and Policy in Mental Health and Mental Health Services Research*, *51*(3), 393–405. https://doi.org/10.1007/s10488-024-01341-x

Stickle, T. R., Kirkpatrick, N. M., & Brush, L. N. (2009). Callous-unemotional traits and social information processing: Multiple risk-factor models for understanding aggressive behavior in antisocial youth. *Law and Human Behavior*, *33*, 515-529. https://doi.org/10.1007/s10979-008-9171-7

Stoppelbein, L., McRae, E., & Smith, S. (2021). Examining student–teacher relationship and callous–unemotional traits in children with adverse childhood

experiences. *School Mental Health*, *13*, 129-142. https://doi.org/10.1007/s12310-020-09397-4

Sukhodolsky, D. G., Ibrahim, K., Kalvin, C. B., Jordan, R. P., Eilbott, J., & Hampson, M. (2022). Increased amygdala and decreased frontolimbic r esting-s tate functional connectivity in children with aggressive behavior. *Social Cognitive and Affective Neuroscience, 17*(7), 634-644. https://doi.org/10.1093/scan/nsab128

Szabó, E., Halasz, J., Morgan, A., Demetrovics, Z., & Kökönyei, G. (2020). Callous-unemotional traits and the attentional bias towards emotional stimuli: Testing the moderating role of emotional and behavioural problems among high-risk adolescents. *Clinical Child Psychology and Psychiatry, 25*(1), 156-173. https://doi.org/10.1177/1359104518822690

Tabares, A. S., Restrepo, J. E., & Montoya, D. A. (2025). The effect of empathy and callous-unemotional traits on externalizing behavior in juvenile offenders: The mediating role of moral disengagement. *International Journal of Social Psychiatry*. https://doi.org/10.1177/00207640251328818

Tan, R., Chen, S., Guo, X., Chen, H., & He, G. (2025). Linking callous–unemotional traits to social withdrawal among young chinese left-behind children: Examining the moderated mediation model. *Behavioral Sciences, 15*(3), 296. https://doi.org/10.3390/bs15030296

Thøgersen, D. M., Bjørnebekk, G., Scavenius, C., & Elmose, M. (2021). Callous-unemotional traits do not predict functional family therapy outcomes for adolescents with behavior problems. *Frontiers in Psychology*, *11*, 537706. https://doi.org/10.3389/fpsyg.2020.537706

Thornton, L. C., Frick, P. J., Ray, J. V., Wall Myers, T. D., Steinberg, L., & Cauffman, E. (2019). Risky sex, drugs, sensation seeking, and callous unemotional traits in

justice-involved male adolescents. *Journal of Clinical Child & Adolescent Psychology*, *48*(1), 68-79. https://doi.org/10.1080/15374416.2017.1399398

Thornberg, R., Wänström, L., Sjögren, B., Pozzoli, T., & Gini, G. (2024). Concurrent associations between callous-unemotional traits, moral disengagement, and bullying perpetration in adolescence. *Journal of Interpersonal Violence*, *40*(5–6), 1459–1483. https://doi.org/10.1177/08862605241260007

Truedsson, E., Fawcett, C., Wesevich, V., Gredebäck, G., & Wåhlstedt, C. (2019). The role of callous-unemotional traits on adolescent positive and negative emotional reactivity: a longitudinal community-based study. *Frontiers in Psychology*, *10*, 573. https://doi.org/10.3389/fpsyg.2019.00573

Urben, S., Habersaat, S., Pihet, S., Suter, M., de Ridder, J., & Stéphan, P. (2018). Specific contributions of age of onset, callous-unemotional traits and impulsivity to reactive and proactive aggression in youths with conduct disorders. *Psychiatric Quarterly*, *89*, 1-10. https://doi.org/10.1007/s11126-017-9506-y

Vaughn, M. G., DeLisi, M., Beaver, K. M., Wexler, J., Barth, A., & Fletcher, J. (2011). Juvenile psychopathic personality traits are associated with poor reading achievement. *Psychiatric Quarterly*, *82*, 177-190. https://doi.org/10.1007/s11126-010-9162-y

Vaughan, E. P., Frick, P. J., Ray, J. V., Thornton, L. C., Myers, T. D. W., Robertson, E. L., Walker, T. M., Steinberg, L., & Cauffman, E. (2025). Predictive and incremental validity of adolescent callous-unemotional traits: longitudinal prediction of antisocial and social outcomes in early adulthood. *Journal of Child Psychology and Psychiatry*. https://doi.org/10.1111/jcpp.14181

Veroude, K., von Rhein, D., Chauvin, R. J., van Dongen, E. V., Mennes, M. J., Franke, B., Heslenfeld, D. J., Oosterlaan, J., Hartman, C. A., Hoekstra, P. J., Glennon, J.

C., & Buitelaar, J. K. (2016). The link between callous-unemotional traits and neural mechanisms of reward processing: An fMRI study. *Psychiatry Research: Neuroimaging*, *255*, 75-80. https://doi.org/10.1016/j.pscychresns.2016.08.005

Viding, E., Sebastian, C. L., Dadds, M. R., Lockwood, P. L., Cecil, C. A., De Brito, S. A., & McCrory, E. J. (2012). Amygdala response to preattentive masked fear in children with conduct problems: the role of callous-unemotional traits. *American Journal of Psychiatry*, *169*(10), 1109-1116. https://doi.org/10.1176/appi.ajp.2012.12020191

Wade, M., Carroll, D., Fox, N. A., Zeanah, C. H., & Nelson, C. A. (2022). Associations between early psychosocial deprivation, cognitive and psychiatric morbidity, and risk-taking behavior in adolescence. *Journal of Clinical Child & Adolescent Psychology*, *51*(6), 850-863. https://doi.org/10.1080/15374416.2020.1864737

Wagner, N. J., Mills-Koonce, W. R., Willoughby, M. T., Zvara, B., & Cox, M. J. (2015). Parenting and children's representations of family predict disruptive and callous-unemotional behaviors. *Developmental Psychology*, *51*(7), 935. https://doi.org/10.1037/a0039353

Walker, T. M., Robertson, E. L., Frick, P. J., Ray, J. V., Thornton, L. C., Myers, T. D. W., Steinberg, L., & Cauffman, E. (2020). Relationships among callous-unemotional traits, future orientation, optimism, and self-esteem in justice-involved adolescents. *Journal of Child and Family Studies*, *29*, 2434-2442. https://doi.org/10.1007/s10826-020-01770-w

Wallace, G. L., White, S. F., Robustelli, B., Sinclair, S., Hwang, S., Martin, A., & Blair, R. J. R. (2014). Cortical and subcortical abnormalities in youths with conduct disorder and elevated callous-unemotional traits. *Journal of the American*

Academy of Child & Adolescent Psychiatry, *53*(4), 456-465. https://doi.org/10.1016/j.jaac.2013.12.008

Waller, R., Dishion, T. J., Shaw, D. S., Gardner, F., Wilson, M. N., & Hyde, L. W. (2016). Does early childhood callous-unemotional behavior uniquely predict behavior problems or callous-unemotional behavior in late childhood?. *Developmental Psychology*, *52*(11), 1805. https://doi.org/10.1037/dev0000165

Walsh, H., Myers, T. D. W., Ray, J. V., Frick, P. J., Thornton, L. C., Steinberg, L., & Cauffman, E. (2019). Perceptions of police-juvenile contact predicts self-reported offending in adolescent males. *Psychology, Crime & Law*, *25*(10), 963-976. https://doi.org/10.1080/1068316X.2019.1597094

Wang, L. A., & Zhou, J. (2023). Violent video game exposure and cyberbullying in early adolescents: a latent moderated mediation model. *Cyberpsychology, Behavior, and Social Networking*, *26*(6), 417-424. https://doi.org/10.1089/cyber.2022.0335

Waschbusch, D. A., Cao, V. T., Shroff, D. M., Bansal, P. S., & Willoughby, M. T. (2024a). Measuring children's reward and punishment sensitivity: an initial psychometric evaluation of the contingency response rating scale. *Assessment, 32*(4), 561–576. https://doi.org/10.1177/10731911241256536

Waschbusch, D. A., Mayes, S. D., Waxmonsky, J. G., Babinski, D. E., & Baweja, R. (2024b). Reactive, proactive, relational, and slow dissipation of aggression in children. *JAACAP Open*, *2*(2), 90–99. https://doi.org/10.1016/j.jaacop.2023.12.007

Waschbusch, D. A., Willoughby, M. T., Haas, S. M., Ridenour, T., Helseth, S., Crum, K. I., Altszuler, A. R., Ross, J. M., Coles, E. K., & Pelham, W. E. (2019). Effects of behavioral treatment modified to fit children with conduct problems and

callous-unemotional (CU) traits. *Journal of Clinical Child & Adolescent Psychology*, *49*(5), 639–650. https://doi.org/10.1080/15374416.2019.1614000

Waxmonsky, J., Fosco, W., Waschbusch, D., Babinski, D., Baweja, R., Pegg, S., Cao, V., Shroff, D., & Kujawa, A. (2022). The impact of irritability and callous unemotional traits on reward positivity in youth with ADHD and conduct problems. *Research on Child and Adolescent Psychopathology*, *50*(8), 1027-1040. https://doi.org/10.1007/s10802-022-00901-9

Werhahn, J. E., Mohl, S., Willinger, D., Smigielski, L., Roth, A., Hofstetter, C., Stämpfli, P., Naaijen, J., Mulder, L. M., Glennon, J. C., Hoekstra, P. J., Dietrich, A., Kleine Deters, R., Aggensteiner, P. M., Holz, N. E., Baumeister, S., Banaschewski, T., Saam, M. C., Schulze, U. M. E., … Brandeis, D. (2020). Aggression subtypes relate to distinct resting state functional connectivity in children and adolescents with disruptive behavior. European Child & Adolescent Psychiatry, 30(8), 1237–1249. https://doi.org/10.1007/s00787-020-01601-9

White, S. F., Brislin, S., Sinclair, S., Fowler, K. A., Pope, K., & Blair, R. J. R. (2013a). The relationship between large cavum septum pellucidum and antisocial behavior, callous-unemotional traits and psychopathy in adolescents. *Journal of Child Psychology and Psychiatry*, *54*(5), 575-581. https://doi.org/10.1111/j.1469-7610.2012.02603.x

White, S. F., Fowler, K. A., Sinclair, S., Schechter, J. C., Majestic, C. M., Pine, D. S., & Blair, R. J. (2014). Disrupted expected value signaling in youth with disruptive behavior disorders to environmental reinforcers. *Journal of the American Academy of Child & Adolescent Psychiatry*, *53*(5), 579-588. https://doi.org/10.1016/j.jaac.2013.12.023

White, S. F., Frick, P. J., Lawing, K., & Bauer, D. (2013b). Callous–unemotional traits and response to Functional Family Therapy in adolescent offenders. *Behavioral Sciences & The Law*, *31*(2), 271-285. https://doi.org/10.1002/bsl.2041

Wilke, J., & Goagoses, N. (2023). Morality in middle childhood: the role of callous-unemotional traits and emotion regulation skills. *BMC Psychology*, *11*(1), 283. https://doi.org/10.1186/s40359-023-01328-7

Winebrake, D., Gueron-Sela, N., Propper, C., Mills-Koonce, R., & Wagner, N. J. (2021). An examination of the relations between effortful control in early childhood and risk for later externalizing psychopathology: A bi-factor structural equation modeling approach. https://doi.org/10.31234/osf.io/5d4sw

Winstanley, M., Webb, R. T., & Conti-Ramsden, G. (2021). Developmental language disorders and risk of recidivism among young offenders. *Journal of Child Psychology and Psychiatry*, *62*(4), 396-403. https://doi.org/10.1111/jcpp.13299

Winters, D. E., & Sakai, J. T. (2023). Affective theory of mind impairments underlying callous-unemotional traits and the role of cognitive control. *Cognition and Emotion*, *37*(4), 696-713. https://doi.org/10.1080/02699931.2023.2195154

Winters, D. E., Pettine, W. W., & Sakai, J. T. (2023). Cognitive mechanisms underlying prosocial decision making in callous-unemotional traits. *Journal of psychopathology and Behavioral Assessment*, *45*(2), 308-321. https://doi.org/10.1007/s10862-023-10043-x

WINTERS ET AL 2025

Wright, M. F., Harper, B. D., & Wachs, S. (2019). The associations between cyberbullying and callous-unemotional traits among adolescents: The moderating effect of online disinhibition. *Personality and Individual Differences, 140,* 41-45. https://doi.org/10.1016/j.paid.2018.04.001

Xie, Q., Bi, T., Du, Y., Kou, H., & Yang, B. (2020). Childhood maltreatment is associated with aggression among male juvenile delinquents in China: The mediating effects of callous-unemotional traits and self-control. *Frontiers in psychology*, *11*, 1373. https://doi.org/10.3389/fpsyg.2020.01373

Xu, W., Wang, M. C., Zhang, X., & Liu, Y. (2022a). The Brief Problem Monitor (BPM-Y/BPM-P) among Chinese youth: psychometric properties and measurement invariance. *Journal of Psychopathology and Behavioral Assessment*, *44*(2), 496-510. https://doi.org/10.1007/s10862-021-09927-7

Xu, W., Wang, M. C., Zhang, X., Zeng, H., & Yang, W. (2022b). Longitudinal pathways between marital relationships and children's CU traits: Parenting style mediation and gender moderation. *Children and Youth Services Review*, *137,* 106456. https://doi.org/10.1016/j.childyouth.2022.106456

Yang, J., Li, W., Wang, W., Gao, L., & Wang, X. (2021). Anger rumination and adolescents' cyberbullying perpetration: Moral disengagement and callous-unemotional traits as moderators. *Journal of Affective Disorders*, *278*, 397-404. https://doi.org/10.1016/j.jad.2020.08.090

Zhang, J., Li, W., Zhang, H., Wilson, A., Shuai, L., Xia, W., Wang, Z., Qiu, M., & Wang, Y. (2021a). Callous-unemotional traits in Chinese preschool children with attention-deficit/hyperactivity disorder. *Child and Adolescent Psychiatry and Mental Health*, *15*, 1-9. https://doi.org/10.1186/s13034-021-00388-0

Zhang, X., Wang, R., Gao, Y., & Wang, M. C. (2021b). Resting heart rate mediates the relationship between parenting style and callous-unemotional traits in Chinese children. *Research on Child and Adolescent Psychopathology*, *49*(11), 1419-1430. https://doi.org/10.1007/s10802-021-00834-9

Zhong, C., Wang, M. C., Shou, Y., Zhang, X., & Deng, J. (2020). Maternal parenting mediate the relationship between maternal psychopathic traits and child callous-unemotional traits: A longitudinal multiple mediation model. *Journal of Child and Family Studies*, *29*, 3142-3152. https://doi.org/10.1007/s10826-020-01814-1

Zumbach, J., Rademacher, A., & Koglin, U. (2021). Conceptualizing callous-unemotional traits in preschoolers: Associations with social-emotional competencies and aggressive behavior. *Child and Adolescent Psychiatry and Mental Health*, *15*(1), 24. https://doi.org/10.1186/s13034-021-00376-4
